# Cost-effectiveness of public-health policy options in the presence of pretreatment NNRTI drug resistance in sub-Saharan Africa: a modelling study

**DOI:** 10.1016/S2352-3018(17)30190-X

**Published:** 2017-11-22

**Authors:** Andrew N Phillips, Valentina Cambiano, Fumiyo Nakagawa, Paul Revill, Michael R Jordan, Timothy B Hallett, Meg Doherty, Andrea De Luca, Jens D Lundgren, Mutsa Mhangara, Tsitsi Apollo, John Mellors, Brooke Nichols, Urvi Parikh, Deenan Pillay, Tobias Rinke de Wit, Kim Sigaloff, Diane Havlir, Daniel R Kuritzkes, Anton Pozniak, David van de Vijver, Marco Vitoria, Mark A Wainberg, Elliot Raizes, Silvia Bertagnolio, Working Group on Modelling Potential Responses to High Levels of Pre-ART Drug Resistance in Sub-Saharan Africa, Andrew N Phillips, Valentina Cambiano, Fumiyo Nakagawa, Paul Revill, Michael R Jordan, Timothy B Hallett, Meg Doherty, Andrea De Luca, Jens D Lundgren, Mutsa Mhangara, Tsitsi Apollo, John Mellors, Brooke Nichols, Urvi Parikh, Deenan Pillay, Tobias Rinke de Wit, Kim Sigaloff, Diane Havlir, Daniel R Kuritzkes, Anton Pozniak, David van de Vijver, Marco Vitoria, Mark A Wainberg, Elliot Raizes, Silvia Bertagnolio

**Affiliations:** a Institute for Global Health, University College London, London, UK; b University of York, York, UK; c Tufts University, Boston, MA, USA; d Imperial College London, London, UK; e WHO, Geneva, Switzerland; f University of Siena, Siena, Italy; g Rigshospitalet, University of Copenhagen, Copenhagen, Denmark; h Ministry of Health & Child Care, Harare, Zimbabwe; i University of Pittsburgh, Pittsburgh, PA, USA; j Department of Global Health, Boston University School of Public Health, Boston, MA, USA; k Africa Health Research Institute, KwaZulu Natal, South Africa; l Division of Infection and Immunity, University College London, London, UK; m Amsterdam Institute for Global Health & Development, San Francisco, CA, USA; n University of California San Francisco, San Francisco, CA, USA; o Brigham and Women's Hospital, Harvard Medical School, Boston, MA, USA; p Chelsea & Westminster Hospital NHS Trust, London, UK; q Erasmus Medical Centre, Rotterdam, Netherlands; r Montreal Jewish General Hospital, Montreal, QC, Canada; s Centers for Disease Control and Prevention, Atlanta, GA, USA

## Abstract

**Background:**

There is concern over increasing prevalence of non-nucleoside reverse-transcriptase inhibitor (NNRTI) resistance in people initiating antiretroviral therapy (ART) in low-income and middle-income countries. We assessed the effectiveness and cost-effectiveness of alternative public health responses in countries in sub-Saharan Africa where the prevalence of pretreatment drug resistance to NNRTIs is high.

**Methods:**

The HIV Synthesis Model is an individual-based simulation model of sexual HIV transmission, progression, and the effect of ART in adults, which is based on extensive published data sources and considers specific drugs and resistance mutations. We used this model to generate multiple setting scenarios mimicking those in sub-Saharan Africa and considered the prevalence of pretreatment NNRTI drug resistance in 2017. We then compared effectiveness and cost-effectiveness of alternative policy options. We took a 20 year time horizon, used a cost effectiveness threshold of US$500 per DALY averted, and discounted DALYs and costs at 3% per year.

**Findings:**

A transition to use of a dolutegravir as a first-line regimen in all new ART initiators is the option predicted to produce the most health benefits, resulting in a reduction of about 1 death per year per 100 people on ART over the next 20 years in a situation in which more than 10% of ART initiators have NNRTI resistance. The negative effect on population health of postponing the transition to dolutegravir increases substantially with higher prevalence of HIV drug resistance to NNRTI in ART initiators. Because of the reduced risk of resistance acquisition with dolutegravir-based regimens and reduced use of expensive second-line boosted protease inhibitor regimens, this policy option is also predicted to lead to a reduction of overall programme cost.

**Interpretation:**

A future transition from first-line regimens containing efavirenz to regimens containing dolutegravir formulations in adult ART initiators is predicted to be effective and cost-effective in low-income settings in sub-Saharan Africa at any prevalence of pre-ART NNRTI resistance. The urgency of the transition will depend largely on the country-specific prevalence of NNRTI resistance.

**Funding:**

Bill & Melinda Gates Foundation, World Health Organization.

## Introduction

More than 18 million people in sub-Saharan Africa are now on antiretroviral therapy (ART). The standard WHO-recommended first-line regimen is the non-nucleoside reverse transcriptase inhibitor (NNRTI) efavirenz plus two nucleoside analogue reverse transcriptase inhibitors (usually lamivudine [3TC] or emtricitabine [FTC]) plus tenofovir disoproxil fumarate (TDF).^1^ Survey results and programme data suggest that more than 85% of people receiving treatment in several countries in southern Africa have viral loads of less than 1000 copies per mL.^2^, ^3^ However, HIV drug resistance, particularly to efavirenz, in people initiating ART has been increasing, with prevalence of more than 10% reported in several countries.^4^, ^5^, ^6^ Individuals with NNRTI-resistant HIV have two to three times greater risk of the ART regimen not achieving and maintaining virus suppression.^7^, ^8^, ^9^ In response to this concern, WHO recommends the routine implementation of pretreatment drug resistance surveys in ART initiators to guide ART programmatic responses in countries with increased prevalence of pretreatment drug resistance.^10^, ^11^, ^12^ ART initiators include those who have never been exposed to antiretroviral drugs and those with previous antiretroviral drug exposure, either because they have previously initiated ART but since interrupted or because they have previously been exposed to antiretroviral drugs for prevention of mother-to-child transmission of HIV. Pretreatment drug resistance is an operational definition and can result from transmitted resistance and acquired resistance developed during previous use of antiretroviral drugs.

**Research in context**

**Evidence before this study**

HIV drug resistance, particularly to efavirenz, in people initiating antiretroviral therapy (ART) is becoming more common, with a prevalence of more than 10% in several countries in sub-Saharan Africa. Individuals with HIV resistant to non-nucleoside reverse transcriptase inhibitor (NNRTI) are at 2–3-fold greater risk of the ART regimen not achieving and maintaining virus suppression. The appropriate public health response to the increased prevalence of NNRTI drug resistance in sub-Saharan Africa is unclear. We searched Web of Knowledge for reports in English and published until Aug 8, 2017, using the following search terms: hiv* AND resistan* AND (efavirenz OR non-nucleoside OR NNRTI) AND cost*. Few studies have addressed cost-effectiveness in the context of sub-Saharan Africa. We did not identify any cost-effectiveness study that included consideration of transition to a dolutegravir-containing first-line ART regimen as an option for responding to high prevalence of NNRTI pretreatment HIV drug resistance.

**Added value of this study**

A transition from efavirenz-based first-line regimens to regimens based on dolutegravir generic formulations in ART initiators is predicted to be effective and cost-effective in low-income settings in sub-Saharan Africa. Benefits are expected to be particularly substantial in populations with high prevalence of HIV drug resistance to NNRTI.

**Implications of all the available evidence**

Countries in sub-Saharan Africa with substantial prevalence of NNRTI drug resistance in ART initiators should begin to plan a managed transition from efavirenz to dolutegravir generic formulations in first-line ART regimens. The urgency of the transition depends on the country-specific prevalence of NNRTI pretreatment HIV drug resistance.

There are various possible policy options in response to increased NNRTI pretreatment drug resistance. One option is to test for HIV drug resistance at treatment initiation, with use of dolutegravir, the WHO-recommended alternative first-line regimen instead of efavirenz if NNRTI resistance is detected. A second option is to transition the standard first-line ART regimen to dolutegravir in all ART initiators, obviating the need for drug resistance testing in new initiators. Both policies could also be considered for implementation only among ART initiators with self-reported antiretroviral drug exposure, rather than in all ART initiators. Each option has different effects on viral suppression, future transmission of HIV drug resistance, and, ultimately, clinical disease, death, and disability-adjusted life-years (DALYs, a measure of overall disease burden that is expressed as the number of healthy years of life lost because of illness, disability, or early death) incurred in the entire adult population. However, responses need to be guided not only by what is predicted to be the most effective option for averting DALYs in the population but also by cost-effectiveness. If the cost per DALY averted is too high, then the resources could probably be used elsewhere in the health-care system to avert more DALYs.

Here we address the policy problem of how low-income countries in sub-Saharan Africa can respond to increasing prevalence of NNRTI pretreatment drug resistance. The findings of this analysis were considered by a WHO Guidelines Development Group tasked to develop guidelines for the public health response to pretreatment HIV drug resistance.

## Methods

### Modelling

We use the HIV Synthesis Model, an individual-based simulation model of HIV transmission, progression, and response to ART that considers specific drugs and resistance mutations and has been used to address several questions in relation to HIV and ART programmes.^13^, ^14^, ^15^ In brief, the model generates a population of adults who are each tracked throughout their lives, at time intervals of 3 months, for HIV testing, condomless sex, and risk of HIV acquisition. Those people who acquire HIV are tracked in terms of their viral load, CD4 cell count, occurrence of WHO stage 3 and 4 HIV disease conditions, clinic attendance and drop-out, use of specific antiretroviral drugs, presence of specific resistance mutations, adherence to ART, and toxic effects of ART. Full details are provided in the appendix. We assumed that viral load monitoring was introduced from 2016, with differentiation of care, using the WHO criteria of a confirmed viral load of more than 1000 copies per mL to define treatment failure.^1^ Individuals meeting the treatment failure criteria switch to a second-line regimen at a rate consistent with the low proportion of people on second-line ART. We initially based the population's demographics and features of the HIV epidemic and programmatic response around those for Malawi, but we ran the model 5000 times, each time sampling a value for a range of parameters (appendix, p 53) to reflect the diversity across populations in sub-Saharan Africa (table). Each of these model runs reflects a different potential programmatic situation, which we refer to as a setting scenario. Within the model, we considered rates of interruption of ART with an associated risk of being lost to follow-up and subsequent chance of returning to care, a chance that is highest if a person becomes sick with a WHO stage 4 condition. For the purposes of this work, we considered those returning to care (ie, when they return to the same or a new clinic) as ART initiators. Additionally, we consider previous use of antiretroviral drugs for prevention of mother-to-child transmission of HIV and associated risk of NNRTI resistance.

**Table:** HIV epidemic and programmatic characteristics of setting scenarios in 2017

		**Median (90% range) across setting scenarios**	**Examples of data from settings**
HIV prevalence (age 15–49 years)		11% (6–22)	Zimbabwe (2015) 14%; Zimbabwe (2016) 14%; Tanzania (2011) 5%; Uganda (2011) 9%; Lesotho (2014) 25%
HIV incidence age 15–49 years per 100 person-years		0·72 (0·15–1·95)	MPHIA (0·37%); ZAMPHIA (0·66%); ZIMPHIA (0·45%); Justman (2·4%); Huerga (0·39%)
Proportion of HIV-positive people diagnosed		79% (60–90)	MPHIA (73%); ZAMPHIA (67%); ZIMPHIA (74%); Huerga (75%); Maman (77%)*
Proportion of all HIV-positive people on ART		63% (44–80)	ZAMPHIA (57%); MPHIA (64%); ZIMPHIA (64%); Maman (68%); Huerga (52%)
Proportion of ART-experienced people who have started second-line (boosted protease inhibitors) ART		4% (0·5–13)	Malawi Ministry of Health (1·5%); Haas (sub-Saharan Africa; 3%)
Proportion of people on ART with viral load less than 1000 copies per mL		83% (71–88)	South Africa (60–88% across districts); ZAMPHIA (89%); MPHIA (91%); ZIMPHIA (87%); Maman (91%); Huerga (90%)
Percentage of ART-naive ART initiators with NNRTI pretreatment drug resistance			Angola (14%); Botswana (8%); South Africa (14%)
	In majority virus	10% (1–34)	
	In minority or majority virus	12% (2–38)	
Percentage of ART initiators with previous exposure to antiretrovirals		18% (8–35)	Proportion of ART initiators is likely to depend on context of discontinuation and re-initiation, which is rarely recorded
	Percentage of ART initiators with previous exposure to antiretrovirals who have NNRTI resistance in majority virus	12% (4–26)	

MPHIA=Malawi population-based HIV impact assessment. ZAMPHIA=Zambia population-based HIV impact assessment. ZIMPHIA=Zimbabwe population-based HIV impact assessment. ART=antiretroviral therapy. NNRTI=non-nucleoside reverse transcriptase inhibitor. A version of this table with references to example data is available in the appendix.

* See also Kim and colleagues,^16^ who suggests undisclosed diagnosed HIV.

For each setting scenario we set 2018 as the baseline year with results from a pretreatment drug resistance survey available to us. We then projected outcomes and costs were the country to implement each of the following alternative policy options, which were formulated as part of the WHO Guidelines Development Group process: (1) no change in policy; (2) for ART initiators with prior antiretroviral exposure, introduce resistance test at treatment initiation and use of dolutegravir if NNRTI resistance is detected; (3) for all ART initiators, introduce resistance test at treatment initiation and use dolutegravir if NNRTI resistance is detected; (4) for ART initiators with previous antiretroviral drug exposure, introduce dolutegravir as first-line regimen; and (5) for all ART initiators, introduce dolutegravir as first-line regimen. We compared predicted outcomes of these policies, including the effectiveness (measured as DALYs) and the cost-effectiveness in the context of different prevalences of NNRTI pretreatment drug resistance.

Properties of dolutegravir were informed by data from many studies (eg, Walmsley and colleagues^17^), and full references for statements in the remainder of this section are given in the appendix (p 3), which also describes our modelling of drug resistance. Illustration of what our assumptions lead to in terms of predicted outcomes by 1 year, 3 years, and 10 years after ART initiation when using a dolutegravir-based or an efavirenz-based regimen are shown in the appendix (p 9). Specific assumptions for dolutegravir were that there is a rate of HIV drug resistance acquisition at a similar level to that of the boosted protease inhibitor atazanavir/r, which is inferred to be 27 times lower than the rate for efavirenz, informed by data on the risk of resistance mutations arising. Dolutegravir monotherapy leads to resistance, albeit at a much lower rate than with efavirenz monotherapy. Dolutegravir has also been generally found to be associated with lower risk of toxicity than both efavirenz and protease inhibitors although it is associated with sleep disturbance in some people. We assume that the risk of neurological toxicity is half that of efavirenz (although we note that the nature of the toxicity is different for the two drugs) and that, unlike efavirenz, dolutegravir is not associated with risk of rash. This reduced risk of toxicity results in a less discontinuation. With respect to potency, we made the conservative assumption that dolutegravir has 1·5-times the potency of efavirenz (lower than boosted protease inhibitors, which are assumed to have potency twice that of efavirenz). Very small studies suggested dolutegravir could be effective as monotherapy but leads to integrase inhibitor resistance over time. When used in combination with lamivudine, dolutegravir is also effective in inducing viral suppression in people who have never experienced virological failure of an ART regimen.

Although these formed our base assumptions, we considered for our main results the possibility of different assumptions for dolutegravir in a small proportion of runs (10% of runs with each assumption): an increased risk of viral load rebound beyond that for efavirenz (not necessarily with HIV drug resistance because of, for example, reduced drug concentration when used together with rifampicin); that potency of dolutegravir could be the same potency or two times the potency of efavirenz; and that the risk of neurological toxic effects of dolutegravir could be equal to that of efavirenz. These small probabilities were selected on the basis that we considered such assumptions unlikely to hold.

In a further sensitivity analysis, we also considered a worst plausible case scenario for dolutegravir. In this case, we assumed a higher risk of viral load rebound than in our base case (ie, we assumed this for 100% of runs rather than for 10% of runs as in our main results). Some reports suggest that risk of immune reconstitution inflammatory syndrome (IRIS) in people with low CD4 cell count at start of ART is greater with dolutegravir than with efavirenz. We thus assumed in our worst plausible case for dolutegravir a 20% risk of IRIS in the first 3 months of dolutegravir-based ART in individuals with a CD4 cell count of less than 100 cells per μL, compared with 5% risk of IRIS for efavirenz. We also assumed that IRIS incurs a $100 hospitalisation cost and is associated with a 5% mortality risk. Although no safety concerns were raised, there is a lack of safety data for dolutegravir in pregnancy. We assumed a 1% risk of drug-related birth defect (compared with no assumed risk for efavirenz), which is assumed to lead to a 0·2 disability weight incurred for 5 years for the mother. Finally, since transition of the first-line regimen could lead to disruption and drug stock-outs, we assumed an increase of five times in the stock-outs in the first year of dolutegravir introduction.

### Cost-effectiveness analysis

Programme costs resulting from the policy options were considered as part of a full economic evaluation. The purpose of a cost-effectiveness analysis is to inform how population health can be improved from within available health-care resources. A health-sector perspective has therefore been adopted, so direct and indirect costs incurred by the patients are not included. We consider a 20 year time perspective from 2018 to 2038. Both costs and health benefits were discounted to present value with a 3% per annum discount rate in our base case. Absolute costs and DALYs are relevant for a country with a population size of about 10 million adults in 2016 (similar to Malawi).

For some analyses, we used net DALYs to compare policy options. This compares the health benefits from a policy with the associated health opportunity costs, resulting from the use of limited resources consequentially being unavailable for other interventions in the public health-care system.^18^ Health opportunity costs are captured by converting the costs to the health-care system into health losses using the cost-effectiveness threshold, which reflects the cost per DALY averted of forgone interventions that can no longer be provided. Net DALYs are calculated as the sum of DALYs plus the ratio of costs to the cost-effectiveness threshold (ie, the health opportunity costs). The cost-effectiveness threshold for a country is not readily apparent, but in most sub-Saharan African settings, $500 per DALY averted is probably at the upper end of the threshold in view of the magnitude of benefit for alternative HIV interventions, but the threshold is likely to be even lower for other health-care activities.^19^, ^20^ The difference in net DALYs between adoption of a strategy and no policy change (incremental net DALYs) shows the net effect of the strategy on population burden of disease. The strategy with the lowest net DALYs incurred overall is deemed cost-effective.

Disability weights to calculate DALYs were derived from a comprehensive dataset (appendix p 63).^21^ Costs of generic antiretroviral formulations used are as follows: $100 per year for efavirenz plus lamivudine plus tenofovir disoproxil fumarate ($38 per year for efavirenz alone); $106 per year for dolutegravir plus lamivudine plus tenofovir disoproxil fumarate ($44 for dolutegravir); and $286 per year for atazanavir/r plus lamivudine plus zidovudine ($213 for atazanavir/r).^22^, ^23^ HIV drug resistance genotyping tests cost $100, as specified by the WHO HIVResNet group. We assume a country transition of first-line regimen of $100 000, which is conceived as the one-off cost incurred in the country for organising and making the transition of regimen, including the training of clinic and pharmacy staff. This cost is uncertain, but programmes have previously transitioned regimens, including from nevirapine to efavirenz and from stavudine to tenofovir disoproxil fumarate. Other unit costs are detailed in the appendix. Modelling and cost-effectiveness analysis was implemented with SAS version 9.4.

### Role of the funding source

The funder of the study had no role in study design, data collection, data analysis, data interpretation, or writing of the report. The corresponding author had full access to all the data in the study and had final responsibility for the decision to submit for publication.

## Results

**Figure 1:**
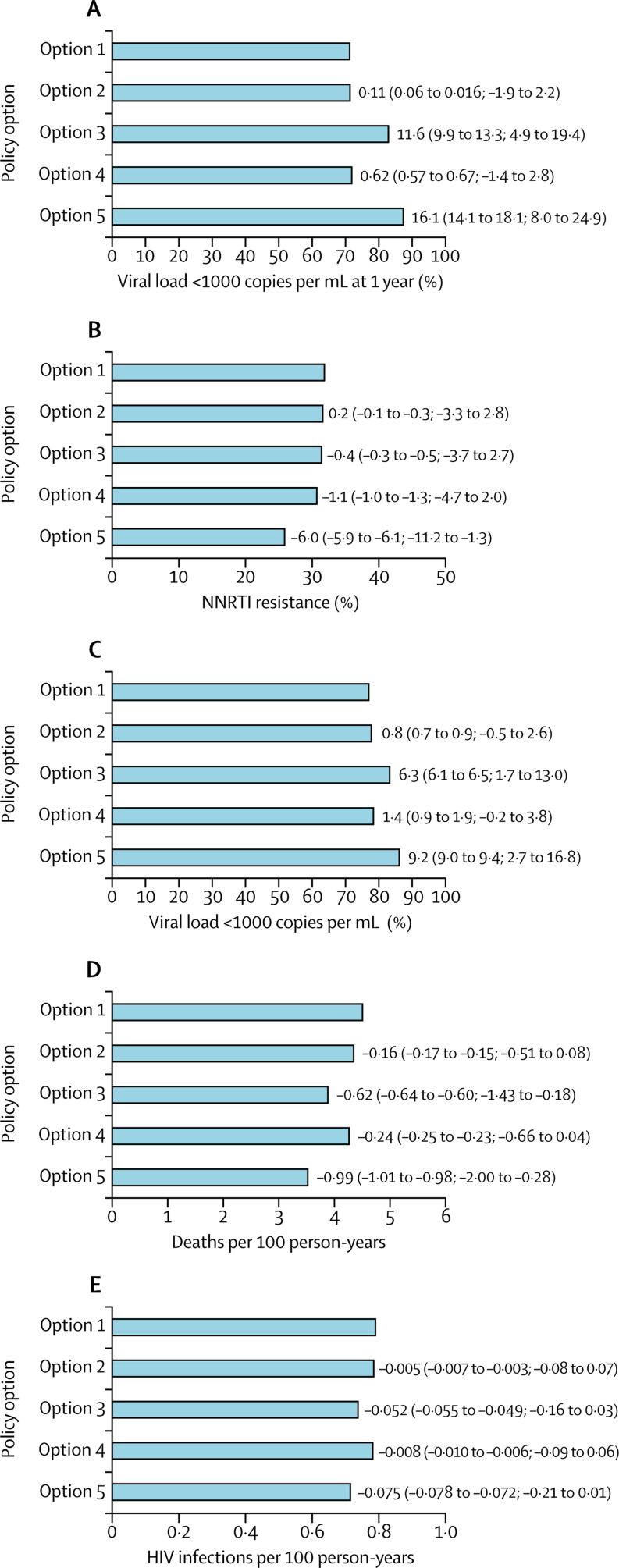
Predicted mean outcomes for 2018–38 according to policy option for setting scenarios where more than 10% of initiators have NNRTI resistance in 2017 (mean over all 3 month periods in 20 years; n=2915) Data are mean differences in percentage relative to no change in policy (95% CI; 90% range). Option 1: no change. Option 2: drug resistance tests for ART initiators with previous antiretroviral exposure. Option 3: drug resistance tests for all ART initiators. Option 4: first-line dolutegravir for people with previous ART exposure. Option 5: first-line dolutegravir for all ART initiators. (A) Mean percent with viral load <1000 copies per mL 1 year from ART initiation. (B) Mean percent of ART initiators with NNRTI resistance (NNRTI pretreatment drug resistance). (C) Mean percent of people with viral load less than 1000 copies per mL at any time after start of ART. (D) Mean mortality in people on ART. (E) Mean incidence of HIV infection. ART=antiretroviral therapy. NNRTI=non-nucleoside reverse transcriptase inhibitor.

The range of HIV epidemic and programmatic characteristics of setting scenarios in 2017 generally reflected those observed in the region (table). We predicted outcomes for the next 20 years according to policy for setting scenarios in which more than 10% (median 19%) of all ART initiators have NNRTI pretreatment drug resistance in 2017 (figure 1). We calculated the mean percentage of ART initiators with viral load less than 1000 copies per mL 1 year from ART initiation over the 20 years and found a substantial predicted positive effect of using dolutegravir in all ART initiators and some positive effect of HIV drug resistance testing, with choice of dolutegravir in those with detected NNRTI pretreatment drug resistance (figure 1A). We also calculated the mean percentage of ART initiators with NNRTI pretreatment drug resistance and found that with no change in policy, the prevalence of NNRTI pretreatment drug resistance is predicted to be more than 30% on average over 2018–38, and only the policy of using dolutegravir in all ART initiators is predicted to substantially reduce NNRTI pretreatment drug resistance (figure 1B). We also found a predicted beneficial effect of dolutegravir for all ART initiators on the mean percentage of all people on ART with viral load of less than 1000 copies per mL and some benefit of pretreatment HIV drug resistance testing, with choice of dolutegravir in individuals with NNRTI resistance (figure 1C). Mortality in people on ART is predicted to follow a similar pattern, with use of dolutegravir in ART initiators leading to a reduction of 1 death per year per 100 people on ART and the use of pretreatment HIV drug resistance testing reducing mortality by 0·6 deaths per year per 100 people on ART (figure 1D). There is also a predicted reduction in HIV infection incidence of about 10% with adoption of the policy of using dolutegravir in all ART initiators (figure 1E).

**Figure 2:**
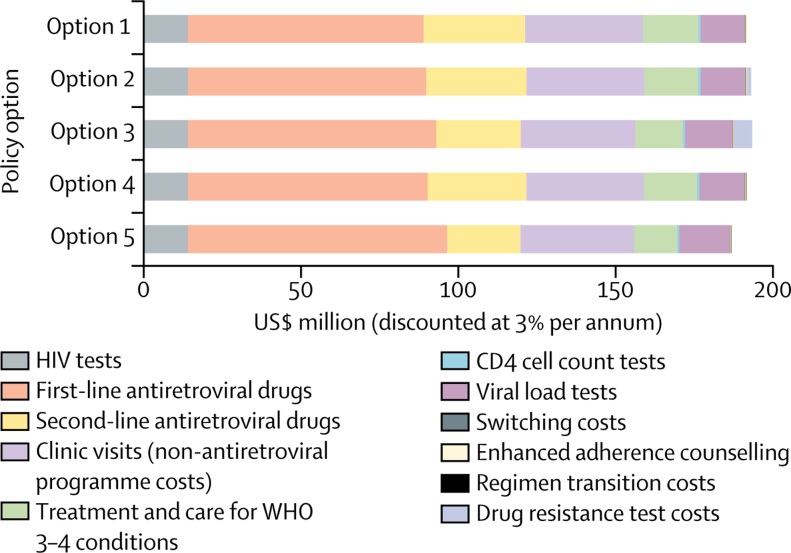
Mean annual cost for 2018–38 according to policy option for setting scenarios where more than 10% of ART initiators have NNRTI resistance in 2017 Option 1: no change. Option 2: drug resistance tests for ART initiators with previous antiretroviral exposure. Option 3: drug resistance tests for all ART initiators. Option 4: first-line dolutegravir for people with previous ART exposure. Option 5: first-line dolutegravir for all ART initiators. 95% CIs are generally narrow (appendix, p 13). ART=antiretroviral therapy. NNRTI=non-nucleoside reverse transcriptase inhibitor.

**Figure 3:**
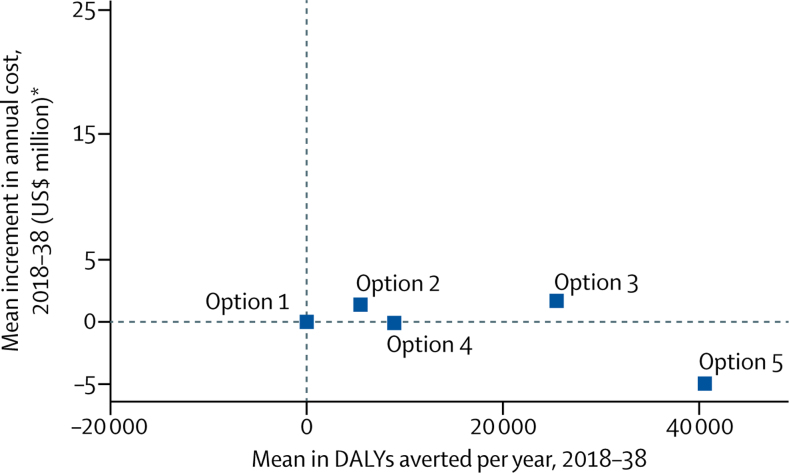
Increment in discounted cost and discounted DALYs averted for each policy option relative to no change (option 1) in setting scenarios where more than 10% of all ART initiators have NNRTI pretreatment drug resistance in 2017 DALYs are for the whole adult population, so this takes into account reductions in new HIV infections associated with increased levels of viral suppression. First-line dolutegravir in all ART initiators is the most cost-effective policy in 90% of the 2815 setting scenarios, using a cost-effectiveness threshold of $500 per DALY averted where more than 10% of ART initiators have NNRTI pretreatment drug resistance in 2017. Incremental net DALYs shows the reduction in population burden of disease, measured in DALYs, per year of each policy compared with no change. The policy that reduces population burden of disease the most (ie, all ART initiators receive first-line dolutegravir) is cost-effective. Option 1: no change. Option 2: drug resistance tests for ART initiators with previous antiretroviral exposure (2857 net DALYs averted). Option 3: drug resistance tests for all ART initiators (22 249). Option 4: first-line dolutegravir for people with previous ART exposure (9190). Option 5: first-line dolutegravir for all ART initiators (50 669). DALYs=disability-adjusted life-years. ART=antiretroviral therapy. NNRTI=non-nucleoside reverse transcriptase inhibitor. *Discounted at 3% per annum.

We analysed the costs of each policy in the context of setting scenarios where more than 10% of ART initiators have NNRTI pretreatment drug resistance in 2017 (figure 2). Despite the slightly higher cost of dolutegravir assumed compared with efavirenz, the policy of using dolutegravir in ART initiators is the lowest-cost policy, primarily because less of the costly second-line boosted protease inhibitor regimen is used. The policy of HIV drug resistance testing with choice of dolutegravir in those with detected NNRTI pretreatment drug resistance also leads to no increase in costs compared with no change in policy, for the same reason. The policy of using dolutegravir in all ART initiators is predicted to avert the most DALYs and has the lowest cost. The association of this policy with the lowest incremental net DALYs suggests it is the policy of choice (figure 3).

So far, all results have focused on setting scenarios in which more than 10% of ART initiators have NNRTI pretreatment drug resistance in 2017. We also considered the cost-effectiveness of the policy alternatives according to the prevalence of NNRTI pretreatment drug resistance (appendix, p 10). The difference in net DALYs compared with no change in policy indicates that the policy of using dolutegravir in ART initiators is the most cost-effective at any prevalence of NNRTI pretreatment drug resistance. The higher the prevalence of NNRTI pretreatment drug resistance, the greater the extent of the benefit in cost-effectiveness by moving to a policy of using dolutegravir in ART initiators.

So far we have considered the overall proportion of all ART initiators with NNRTI pretreatment drug resistance. We also assessed the most cost-effective policy according to both the percentage of ART initiators with prior antiretroviral resistance with NNRTI pretreatment drug resistance and the percentage of ART initiators without prior antiretroviral drug exposure with NNRTI pretreatment drug resistance, each in 2017 (appendix, p 11). In each case, the policy of using dolutegravir in ART initiators is the most cost-effective.

**Figure 4:**
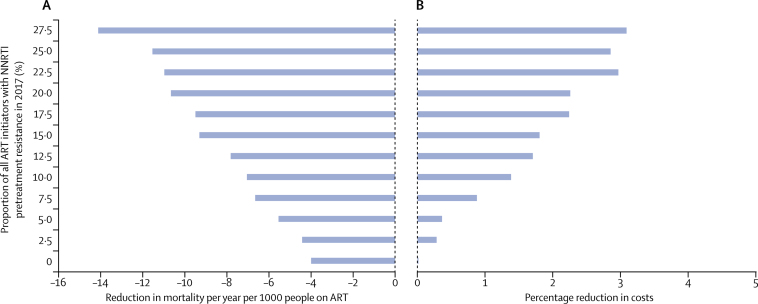
Reductions in mortality and cost associated with use of dolutegravir in ART initiators rather than efavirenz (A) Difference in mortality (per 1000 people on ART per year) for 2018–38 when using dolutegravir in ART initiators versus continuing with efavirenz-based ART, according to proportion of all ART initiators with NNRTI resistance in 2017. 95% CIs are narrower than +/– 0·1. (B) Percentage reduction in annual costs for the policy of using dolutegravir in ART initiators versus continuing with efavirenz-based ART, according to proportion of all ART initiators with NNRTI resistance in 2017. 95% CIs are narrower than +/– 0·4. ART=antiretroviral therapy. NNRTI=non-nucleoside reverse transcriptase inhibitor.

We then considered the absolute difference in mortality (number of deaths per 1000 people on ART per year) and the percentage difference in cost for the policy of using dolutegravir instead of efavirenz in all ART initiators, according to the proportion of all ART initiators with NNRTI pretreatment drug resistance in 2017 (figure 4). We found a mortality benefit of the policy of using dolutegravir in all ART initiators even in the category with lowest NNRTI pretreatment drug resistance, reflecting the benefit of dolutegravir observed in randomised trials (appendix p 4) of people without prior ART resistance. However, the extent of benefit from transition to such a policy improves substantially with increasing NNRTI pretreatment drug resistance. For setting scenarios with a prevalence of NNRTI pretreatment drug resistance of 10–12·5% in 2017, no change and failure to adopt a policy of using dolutegravir in all ART initiators translates into a median of 5500 additional deaths per year (90% range 1050–16 750) on average in the next 20 years. For setting scenarios with a prevalence of NNRTI pretreatment drug resistance of 17·5–20%, 9000 additional deaths per year (2600–20 650) are expected in the next 20 years if there is no change in policy and a policy of using dolutegravir in all ART initiators is not adopted.

In the sensitivity analysis, we found that a worst plausible case scenario for the properties of generic dolutegravir (while maintaining an annual cost of dolutegravir of $44) has little effect on our overall conclusions, with the change in policy to use dolutegravir in all ART initiators predicted to be the most effective policy and to be cost-saving (appendix, p 11).

## Discussion

The results of our modelling-based analysis predict that transition to use of dolutegravir instead of efavirenz in regimens for all ART initiators is likely to bring health benefits irrespective of the level of NNRTI pretreatment drug resistance because of its higher potency, lower risk of selection for resistance, and lower incidence of discontinuation because of toxicity. However, the extent of benefits is predicted to be greatest in settings with high levels of NNRTI pretreatment drug resistance. Programmes in settings with even moderately increased NNRTI pretreatment drug resistance should plan to transition to use dolutegravir as a first-line drug; as well as being cost-effective, this is likely to also be cost saving. The appropriate timescale for such plans will depend on several factors, of which the actual current level of NNRTI pretreatment drug resistance is an important one. A generic, fixed-dose combination of dolutegravir plus lamivudine plus tenofovir disoproxil fumarate is expected to be available in 2018. A recent announcement^24^ suggests that the cost of this regimen has become lower than that assumed in our modelling ($75 per year compared with $106), suggesting an even greater cost saving than that we projected. As a result of the WHO Guidelines Development Group process, at which these results were presented, a consensus statement was made that countries in which the prevalence of pretreatment HIV drug resistance to NNRTIs among people initiating first-line ART, irrespective of previous ARV drug exposure, is 10% or more should urgently consider an alternative first-line ART regimen that does not contain NNRTIs.^9^ The WHO alternative first-line drug to an NNRTI in the 2016 guidelines is dolutegravir.

Although recommended as part of first-line therapy in high income settings,^25^ some uncertainties remain over dolutegravir use. Data on safety in pregnancy are only just accumulating.^26^ There is also a concern that an interaction between dolutegravir and the tuberculosis drug rifampicin could reduce drug levels when used concomitantly, leading to excess risk of virological failure if the dose of dolutegravir is not increased.^27^ It remains unclear whether the extent of the reduction is such that that increasing the dolutegravir dose (from once to twice a day) is required when coadministered with rifampicin, given that dolutegravir is known to be efficacious at a dose one fifth of the standard dose (appendix, p 6). Lastly, some reports suggest that dolutegravir initiated in people with low CD4 cell count is associated with an increased risk of IRIS, which would result in increased health-care costs and a small excess mortality risk (appendix, p 8). We factored these risks into our worst plausible case for dolutegravir, but they did not change our conclusion. Venter and colleagues^28^ discussed prospects for transitioning to new regimens in sub-Saharan Africa, and transition has already started in Botswana and Brazil.

Although we considered a policy of using dolutegravir in all new ART initiators, we could also have considered a wider introduction of the drug for all people on first-line efavirenz-based ART. This policy is predicted to bring further health benefits,^29^ and ART programmes might find it easier to make a wholesale transition in all people on first-line ART, but we do not present results on this potential policy here. Likewise, here we have not considered the possibility of use of dolutegravir in second-line regimens, as considered elsewhere.^30^ We have focused on changes in policy to deal with the fact that, due to existence of NNRTI pretreatment drug resistance, the first-line ART regimen will not be fully effective in some people. However, the identification of high levels of pretreatment drug resistance should also prompt consideration of other programmatic improvements, such as strengthening of adherence, supporting retention, and increasing switching to second-line regimens in people with virological failure.

We are not aware of other studies of cost-effectiveness of transition to dolutegravir in sub-Saharan Africa. In a recent study of the cost-effectiveness of pretreatment drug resistance testing in Kenya,^31^ a low-cost point mutation assay ($30) before ART initiation was predicted to be very cost-effective. Such assays (adapted for integrase mutations) might eventually have a role in testing for resistance in people with increased viral load on first-line dolutegravir to identify if there is a need to switch regimen.

Modelling studies of projected mortality differences between available policy options become necessary when data on direct benefits of dolutegravir are only available for potential intermediate outcomes of viral load, resistance, and toxicity. The fact that differences in the ability of drug regimens to result in viral suppression does ultimately translate into differences in risk of AIDS and death is the basis of the reason why the FDA in 1997 moved from approving antiretroviral drugs on the basis of clinical endpoint data to approving them on the basis of viral load endpoint data.^32^

In our model, we capture the fact that some people discontinue ART and become disengaged from care and that this is more likely in people who are poorly adherent or for whom ART is failing, or both. These people are therefore likely to have NNRTI drug resistance.^33^, ^34^, ^35^ Nevertheless, model outputs for the proportion of ART initiators with NNRTI pretreatment drug resistance who have previous antiretroviral drug exposure were not substantially higher than for those who have no previous antiretroviral drug exposure, which is in contrast with survey findings of pretreatment drug resistance that show markedly higher levels. This implies an even greater tendency for interruption of ART to be more likely in those with poor adherence than was assumed, which might imply that the policies just targeted at those with previous antiretroviral drug exposure could be more effective than we have shown.

Our work has several limitations. As is inevitable for a cost-effectiveness analysis with an appropriately long-time horizon, which is required to consider the future consequences of current decisions, we rely on a model to give predictions of the long-term effect of the alternative policies. Our model is particularly detailed and relies on many assumptions, although these have generally been well informed by a wide array of observed data, albeit not always directly from the region itself. The benefits of dolutegravir compared with efavirenz are also well supported by data. We note that, since our model includes adults only, the potential beneficial effects of dolutegravir for people younger than 15 years have not been included, and we did not consider effects on transmission to children. Furthermore, our focus is on low-income settings in sub-Saharan Africa. Generic dolutegravir is unavailable elsewhere, and cost-effectiveness of the transition is likely to be country-specific and dependent on the cost of dolutegravir and to be affected by costs of other drugs and availability of HIV drug resistance testing capacity. Although drug prices can decrease over time, there is generally a floor price determined by the production cost, so generic drug prices might well not now decrease markedly over time in the future.

In conclusion, a transition from efavirenz-containing first-line regimens to generic dolutegravir-containing regimens is predicted to be effective and cost-effective in low-income settings in sub-Saharan Africa at any level of NNRTI pretreatment drug resistance. However, with high prevalence of NNRTI pretreatment drug resistance, the negative effect on the population health of postponing the transition to dolutegravir increases substantially and thus increases the urgency of this intervention.
