# Supplementary material for: Cost-effectiveness of public-health policy options in the presence of pretreatment NNRTI drug resistance in sub-Saharan Africa: a modelling study
Source: Lancet HIV. 2017 Nov 22;5(3):e146–54. doi: 10.1016/S2352-3018(17)30190-X (PMC5843989; doi:10.1016/S2352-3018(17)30190-X)
Supplement: Supplementary appendix [file mmc1.pdf]

THE LANCET
HIV

Supplementary appendix

This appendix formed part of the original submission and has been peer reviewed. We post it as supplied by the authors.

Supplement to: Phillips A N, Cambiano V, Nakagawa F, et al. Cost-effectiveness of public-health policy options in the presence of pretreatment NNRTI drug resistance in sub-Saharan Africa: a modelling study. *Lancet HIV* 2017; published online Nov 22. http://dx.doi.org/10.1016/S2352-3018(17)30190-X.

APPENDIX

Cost-effectiveness of public health policy options in the presence of pre-treatment NNRTI drug resistance in sub-Saharan Africa: a modelling study

Contents

1. **Supplementary description of methods for current analysis, supplementary tables and figures**
2. **Modelling details**

1. Supplementary description of methods for current analysis, supplementary tables and figures

Modelling approach

The following is a fuller description of the modelling approach used in the paper. Full details of the model are provided separately below in section 2.

We use the HIV Synthesis Model, an individual-based simulation model of HIV transmission, progression and the effect of ART, considering specific drugs and resistance mutations, and which has been used to address several questions in relation to HIV and ART programs (e.g. [1-3]). The model generates a population of adults who are each tracked for HIV testing, condomless sex and risk of HIV acquisition, and then those who acquire HIV are tracked in terms of their viral load, CD4 count, occurrence of WHO stage 3 and 4 conditions, clinic attendance and drop-out, current use of specific ARVs, presence of specific resistance mutations, adherence to ART and toxicities from ART. These are updated every 3 months. We assume that from 2016 viral load monitoring, with differentiation of care[3], is introduced, using the WHO criteria of a confirmed value > 1000 copies/mL to define failure.

Individuals meeting the failure criteria switch to a 2nd-line regimen at a rate consistent with the low proportion of people on 2nd-line ART. The modelled structure of the relationship between ART adherence, viral load, development of resistance, CD4 count and risk of AIDS and death is illustrated in Supplementary Figure 1. The adherence level influences the risk of acquisition of new mutations as well as having a direct effect on the viral load and CD4 count. Full details of the model are provided in the section 2.

We initially based the demographics of the population studied and HIV epidemic and ART program features around those for Malawi, but we ran the model 5000 times, each time sampling a value for a range of parameters to reflect the diversity across populations in sub-Saharan Africa. These parameters included those determining condomless sex with short- and long-term partners, the relative level of HIV testing, the rate of pregnancy, the probability of immediate loss at HIV diagnosis, the rate of loss to follow-up for people off ART, the date of ART introduction, the probability of ART initiation given eligibility, the use of different PMTCT regimens over the years, the rate of ART interruption and being lost to the clinic, and the ART adherence pattern distribution. Each of these model runs reflects a different potential programmatic situation which we call a *setting scenario*.

Within the model we consider rates of interruption of ART with an associated risk of being lost to follow-up and subsequent chance of returning to care, a chance which is highest if a person becomes sick with a WHO stage 4 condition. For the purposes of this work, we consider those returning to care (i.e. when they return to the same or a new clinic) as ART initiators. In addition, we consider previous use of ARVs for PMTCT and associated risk of NNRTI resistance. For each setting scenario we track all relevant outputs over time, including the level of baseline NNRTI PDR in ART initiators.

For each setting scenario we then place ourselves in the position of being in 2018 with results from WHO PDR surveys available to us. We consider this our baseline year. We then project the outcomes that would be predicted to occur, and the costs that would be incurred, were the country to implement each of the alternative policy options. The policy options considered were: (i) no change in policy, (ii) for ART initiators with prior ARV exposure: resistance test at treatment initiation – use dolutegravir if NNRTI resistance detected, (iii) for all ART initiators: resistance test at treatment initiation – use dolutegravir if NNRTI resistance detected, (iv) for ART initiators with prior ARV exposure: dolutegravir as 1st line regimen, and (v) for all ART initiators: dolutegravir as 1st line regimen.

The outcomes over 20 years that are generated for each policy option include the proportion of ART initiators with viral suppression by 12 months, the level of PDR in all ART initiators, the death rate in those on ART, and the overall disability-adjusted life years (DALYs) in the adult population. We compare predicted outcomes of

these policies, including the effectiveness (measured as DALYs averted compared with no change in policy), together with the cost-effectiveness in the context of different levels of NNRTI PDR. DALYs are calculated within the model itself – DALYs due to premature death are accumulated after a person's death, until they would have reached age 65 had they been alive. Since ours is a model of adults only we did not consider effects on transmission to children or DALYs in children.

Modelling of ART and HIVDR

HIVDR is modelled in terms of the presence or absence of mutations specific to ARVs in use. Distinction is made for each mutation as to whether it is present only in low abundance, and thus assumed non-transmissible, or if it is present in majority virus, and hence transmissible. The probability of selection of drug-resistant virus among people on ART is determined by the number of active drugs in the regimen (determined by presence of relevant HIVDR mutations), viral load, and the individual's current ART adherence. Mutations acquired while on ART are lost from majority virus (at a mutation-specific rate) when there is no longer a drug being taken that selects for it, although these mutations remain in minority virus. Mutations present in minority virus re-emerge in majority virus when one of the corresponding drugs is started. The probability of transmission of HIV from a condomless sex partner depends on the viral load in the source partner. The presence of HIVDR in the partner does not directly influence the risk of transmission, only via the effect on viral load if the partner is on ART. For a newly-infected person, the probability that the source partner has resistant virus in the majority circulating virus is determined by the prevalence of HIVDR among those with HIV currently having condomless sex. Not all HIVDR mutations present in majority virus in the source partner are established in the circulating virus of the newly infected person. The probability of transmission of HIVDR mutations is mutation-specific. Once virus with a mutation is transmitted and established in the new host, there is a tendency for a loss of drug-resistant mutation from majority virus over time, again mutation-specific.

In calculating the current number of active drugs, we assume by default that each drug has potency (i.e. the intrinsic ability of a drug to suppress replication of non-resistant virus) such that it counts as one active drug when no HIVDR mutations for that drug are present. However, since ritonavir-boosted protease inhibitors (PI) have shown efficacy in inducing a high level of viral suppression when used as a single drug[18] we assume that such drugs have a potency which is 2-fold higher.

Assumptions on properties of dolutegravir compared with efavirenz

Properties of dolutegravir were informed by data from a number of randomized trials and observational studies[5-23]. Illustration of what our assumptions lead to in terms of predicted outcomes by 1, 3, and 10 years of start of ART when using a dolutegravir and an efavirenz-based regimen are shown in Supplementary Figure 2. Specific assumptions for dolutegravir include that there is a rate of HIVDR acquisition of a similar level to that of the boosted PI atazanavir/r, which is inferred to be 27 times lower than the rate for efavirenz, informed by data on the risk of resistance mutations arising[5-9, 16-24]. Dolutegravir monotherapy has been shown to lead to resistance, albeit at a much lower rate than would be the case with efavirenz monotherapy[22-23]. Dolutegravir has been generally found to be associated with lower risk of toxicity than either efavirenz[5,6] or Pis[7] although it is associated with sleep disturbance in some people[13-15]. We assume that the risk of neurologic toxicity is half that of efavirenz (although noting that the nature of the toxicity is different for the two drugs), and that, unlike efavirenz, it is not associated with risk of rash. This lower rate of toxicity results in a lower rate of discontinuation[6,9]. Regarding potency, we made the conservative assumption that dolutegravir has 1.5-fold higher potency than efavirenz (i.e. lower than a boosted PI). Dolutegravir has been shown in very small studies to be initially effective as monotherapy[19,20], although leading to integrase inhibitor resistance over time[22,23], and to be effective in inducing viral suppression in ART-naïve people when used with just lamivudine, with little evidence thus far of integrase inhibitor resistance in this latter situation[25-27].

Although these formed our base assumptions, we considered for our main results the possibility of different assumptions for dolutegravir in a small proportion of runs, i.e. a raised risk of viral load rebound beyond that for efavirenz (not necessarily with HIVDR, e.g. due to lowering of drug levels due to co-use with rifampicin) (10% of runs), that potency of dolutegravir could be instead 1-fold or 2- fold (10% of runs in each case); and that the risk of neurologic toxicity using dolutegravir could be equal to that of efavirenz (10% of runs). These small probabilities were selected on the basis that we considered such assumptions unlikely to hold.

Cost-effectiveness analysis

Programme costs resulting from the policy options are also considered to allow a full economic evaluation. The purpose of performing cost-effectiveness analysis is to inform how we can improve population health from within available health care resources. A health sector perspective has therefore been adopted, so direct and indirect costs incurred by the patients are not included. Health benefits associated with the policies are estimated using the metric *DALYs averted* in the entire adult population. We consider a 20 year time perspective from 2018-2038. Both costs and health benefits were discounted to present value using a 3% per annum discount rate in our base case. Absolute costs and DALYs are relevant for a country of population size of approx 10 million adults in 2016 (similar to Malawi).

We use a measure to compare policy options called net DALYs. This compares the health benefits from a policy with the associated health opportunity costs, resulting from the use of limited resources consequentially being unavailable for other interventions in the public health care system[28]. Health opportunity costs are captured by converting the costs falling on the health care system into health losses using the cost-effectiveness threshold, which reflects the cost-per-DALY-averted of forgone interventions that can no longer be provided. Net DALYs are calculated as the ratio of costs to the cost effectiveness threshold (i.e. the health opportunity costs) added to the DALYs-incurred (so net DALYs = DALYS + cost / cost-effectiveness threshold). The threshold for a country is not readily apparent, but in most sub-Saharan Africa settings $500 per DALY averted is likely to be at the upper end based on the magnitude of benefit for alternative HIV interventions, but may be even lower for other health care activities.[19,30] The difference in net DALYs from a strategy compared to no policy change (incremental net DALYs) shows its net impact on population burden of disease. The strategy with the lowest net DALYs incurred overall is deemed cost-effective.

Disability weights to calculate DALYs were derived from a comprehensive study[31] and are detailed in Table S18. Costs of generic ARV formulations used are as follows: efavirenz + lamivudine + tenofovir $100 per year ($38 for efavirenz); dolutegravir + lamivudine + tenofovir $106 ($44 dolutegravir); atazanavir/r + lamivudine + zidovudine $286 (atazanavir/r $213) [32-34]. HIVDR genotype test cost $100, specified by the WHO HIVResNet group. We assume a country transition of 1st-line regimen of $100,000, which is conceived of as the one-off cost incurred in the country for organising and making the transition of regimen, including the training of clinic and pharmacy staff. This cost is uncertain but programmes have previously transitioned regimens, including from nevirapine to efavirenz and stavudine to tenofovir so there is experience in this. Other unit costs are detailed in Section 2.10.

Sensitivity analyses

In sensitivity analysis we considered also a worst plausible case scenario for dolutegravir. In this case we assumed a higher risk of viral load rebound than in our base case (i.e. we assumed this for 100% of runs rather than 10% for our main results). There are reports suggesting that risk of immune response inflammatory syndrome (IRIS) in people with low CD4 count at start of ART is greater with dolutegravir than efavirenz[,35-37]. We thus assumed in our worst plausible case for dolutegravir a 20% risk of IRIS in the first 3 months of dolutegravir-based ART in those with a CD4 count below 100 cells /mm^3, compared with 5% for efavirenz, and that IRIS incurs

a $100 hospitalisation cost and is associated with a 5% mortality risk. Although there have been no safety concerns flagged, there is a lack of safety data for dolutegravir in pregnancy. We assumed a 1% risk of drug-related birth defect risk (assumed zero for efavirenz), which is assumed to lead to a 0.2 disability weight incurred for 5 years for the mother. Lastly, since transition of the 1st-line regimen could lead to disruption and drug stock-outs we assumed a 5-fold increase in the rate of stock-outs in the first year of dolutegravir introduction.

References for Modelling Approach

1. Cambiano V, Bertagnolio S, Jordan M, Pillay D, Perriens J, Venter F, et al. Predicted levels of HIV drug resistance: potential impact of expanding diagnosis, retention, and eligibility criteria for antiretroviral therapy initiation. AIDS 2014, 28 (Suppl 1):S15–S23.
2. Phillips AN, Cambiano V, Miners A, Revill P, Pillay D, Lundgren JD, et al. Effectiveness and cost-effectiveness of potential responses to future high levels of transmitted HIV drug resistance in antiretroviral drug-naive populations beginning treatment: modelling study and economic analysis. Lancet HIV 2014 October 14, 2014
3. Working Group on Modelling of Antiretroviral Therapy Monitoring Strategies in Sub-Saharan Africa. Sustainable HIV treatment in Africa through viral-load-informed differentiated care. Nature 2015; 528:S68–76.
4. Perez-Valero I, Arribas JR. Protease inhibitor monotherapy. Cur Opinion in Infect Dis 2011; 24: 7-11 DOI: 10.1097/QCO.0b013e3283422cdf
5. Walmsley SL, Antela A, Clumeck N, Duiculescu D, Eberhard A, Gutierrez F, et al. Dolutegravir plus abacavir-lamivudine for the treatment of HIV-1 infection. N Engl J Med. 2013; 369(19):1807–18. doi: 10.1056/NEJMoa1215541 PMID: 24195548
6. Walmsley S, Baumgarten A, Berenguer J, Felizarta F, Florence E, Khuong-Josses MA, et al. Brief Report: Dolutegravir Plus Abacavir/lamivudine for the Treatment of HIV-1 Infection in Antiretroviral Therapy-Naive Patients: Week 96 and Week 144 Results From the SINGLE Randomized Clinical Trial. J Acquir Immune Defic Syndr. 2015;70(5):515–9. doi: 10.1097/QAI.0000000000000790. pmid:26262777
7. Clotet B, Feinberg J, van Lunzen J, Khuong-Josses MA, Antinori A, Dumitru I, et al. Once-daily dolutegravir versus darunavir plus ritonavir in antiretroviral-naive adults with HIV-1 infection (FLAMINGO): 48 week results from the randomised open-label phase 3b study. Lancet. 2014; 383(9936):2222–31. doi: 10.1016/S0140-6736(14)60084-2 PMID: 24698485
8. van Lunzen J, Maggiolo F, Arribas JR, Rakhmanova A, Yeni P, Young B, et al. Once daily dolutegravir (S/GSK1349572) in combination therapy in antiretroviral-naive adults with HIV: planned interim 48 week results from SPRING-1, a dose-ranging, randomised, phase 2b trial. Lancet Infect Dis 2012; 12: 111–18
9. Stellbrink H, Reynes J, Lazzarin A, Voronin E, Pulido F, Felizarta F, et al. Dolutegravir in antiretroviral-naive adults with HIV-1: 96-week results from a randomized dose-ranging study. AIDS 2013; 27:1771–1778
10. Sax PE, DeJesus E, Crofoot G, Ward D, Benson P, Dretler R. Bictegravir versus dolutegravir, each with emtricitabine and TDF alafenamide, for initial treatment of HIV-1 infection: a randomised, double-blind, phase 2 trial. Lancet HIV 2017; 4: e154–60
11. Rutherford GW, Horvath H (2016) Dolutegravir Plus Two Nucleoside Reverse Transcriptase Inhibitors versus Efavirenz Plus Two Nucleoside Reverse Transcriptase Inhibitors As Initial Antiretroviral Therapy for People with HIV: A Systematic Review. PLoS ONE 11(10): e0162775. doi:10.1371/journal.pone.0162775
12. Patel DA, Snedecor SJ, Tang WY, Sudharshan L, Lim JW, et al. 48-Week Efficacy and Safety of Dolutegravir Relative to Commonly Used Third Agents in Treatment-Naive HIV-1–Infected Patients: A Systematic Review and Network Meta-Analysis. PLoS ONE 2014 9(9): e105653. doi:10.1371/journal.pone.0105653.

13. de Boer M, van den Berk GEL, van Holten N, Oryszcyn JE, Dorama W, ait Mohab D, et al. Intolerance of dolutegravir-containing combination antiretroviral therapy regimens in real-life clinical practice. AIDS 2016, 30:2831–2834.
14. Hoffmann C, Welz T, Sabranski M, Kolb M, Wolf E, Stellbrink1 H-J et al. Higher rates of neuropsychiatric adverse events leading to dolutegravir discontinuation in women and older patients. HIV Medicine 2017; 18, 56—63.
15. Menard A, Montagnac C, Solas C, Meddeb L, Dhiver D, Tomeia C, et al. Neuropsychiatric adverse effects on dolutegravir: an emerging concern in Europe AIDS 2017, Vol 31 No 8
16. Taha H, Das A, Das S. Clinical effectiveness of dolutegravir in the treatment of HIV/AIDS. Infect Drug Resist. 2015;8:339–52. doi: 10.2147/IDR.S68396. pmid:26491363
17. Llibre JM, Pulido F, Garcia F, Garcia Deltoro M, Blanco JL, Delgado R. Genetic barrier to resistance for dolutegravir. AIDS Rev. 2015; 17(1):56–64. PMID: 25472016 7.
18. Marcllin A, Grude G, Charpentier C, Bellecave P, Rodallec A, Pallier C, et al. French survey, resistance to integrs inhib shows differences of resistance selection in virological failure in care. Abst 332. HIV Glasgow Octover 2016.
19. Nicolè S, Lanzafame M, Lattuada E, Mazzi R, Rigo F, Cucchetto G, et al. Dolutegravir montherapy in HIV-infected naïve patients with <100,000 copies/ML HIV RNA load, an update of a little cohort in Verona. Infect Dis Trop Med 2016; 2 (2): e295.
20. Katlama C, Soulie C, Caby F, Denis A, Blanc C, Schneider L, et al. Dolutegravir as monotherapy in HIV-1-infected individuals with suppressed HIV viraemia. JOURNAL OF ANTIMICROBIAL CHEMOTHERAPY 2016; 71: 2646-2650 DOI: 10.1093/jac/dkw186.
21. Wainberg MA, Han YS. Will drug resistance against dolutegravir in initial therapy ever occur? Front Pharmacol. 2015; 6:90. doi: 10.3389/fphar.2015.00090 PMID: 25972810
22. Blanco J et al. Pathways of resistance in subjects failing dolutegravir monotherapy. CROI 2017, 13-16 February 2017, Seattle. Oral abstract 42. http://www.croiconference.org/sessions/pathways-resistance-subjects-failing-dolutegravir-monotherapy (abstract) http://www.croiwebcasts.org/console/player/33379 (webcast)
23. Wijting I et al. Dolutegravir as maintenance monotherapy for HIV-1: a randomized clinical trial . (NCT02401828). CROI 2017, 13-16 February 2017, Seattle. Late breaker poster abstract 451LB.http://www.croiconference.org/sessions/dolutegravir-maintenance-monotherapy-hiv-1-randomized-clinical-trial (abstract and poster)
24. Cozzi-Lepri, UK HIV Drug Resistance Database. Long-Term Probability of Detecting Drug-Resistant HIV in Treatment-Naive Patients Initiating Combination Antiretroviral Therapy. Clin Infect Dis 2010; 50(9):1275–1285
25. Maggiolo F, Gulminetti R, Pagnucco L, Digaetano M, Benatti S, Valenti D, et al. Lamivudine/dolutegravir dual therapy in HIV-infected, virologically suppressed patients. BMC Inf Dis 2017; 17 DOI: 10.1186/s12879-017-2311-2
26. Gubavu C, Prazuck T, Niang M, Buret J, Mille C, Guinard J, Avettand-Fenoel V, Hocqueloux L et al. Dolutegravir-based monotherapy or dual therapy maintains a high proportion of viral suppression even in highly experienced HIV-1-infected patients. J Antimicr Chem 2016; 71: 1046-1050. DOI: 10.1093/jac/dkv430
27. Borghetti A, Baldin G, Ciccullo A, Gagliardini R, Davino A, Mondi A, et al. Virological control and metabolic improvement in HIV-infected, virologically suppressed patients switching to lamivudine/dolutegravir dual therapy. J Antimicrob Chem 2016; 71: 2359-2361. DOI: 10.1093/jac/dkw147
28. Claxton K, Walker S, Palmer S, Sculpher M. ‘Appropriate Perspectives for Health Care Decisions’ Centre for Health Economics Research Paper 54 University of York 2010
29. Woods E, Revill P, Sculpher M, Claxton K. Country-Level Cost- Effectiveness Thresholds: Initial Estimates and the Need for Further Research.

https://www.york.ac.uk/media/che/documents/papers/researchpapers/CHERP109_cost-effectiveness_threshold_LMICs.pdf

30. Ochalek, JM, Claxton, KP, Revill, P, Sculpher, MJ & Rollinger, A 2016 'Supporting the development of an essential health package: principles and initial assessment for Malawi' CHE Research Paper, no. 136, Centre for Health Economics, University of York, York, UK, pp. 1-90.
31. Salomon JA, Vos T, Hogan DR, Gagnon M, Naghavi M, Mokdad A et al. Common values in assessing health outcomes from disease and injury: disability weights measurement study for the Global Burden of Disease Study 2010. Lancet 2012; 380: 2129–43.
32. MSF. Untangling the web of antiretroviral price reductions. 18th Edition – July 2016
33. CHAI. ARV Market Report: The State of the Antiretroviral Drug Market in Low- and Middle-Income Countries, 2015-2020 Issue 7, October 2016.
34. http://www.unaids.org/en/resources/presscentre/pressreleaseandstatementarchive/2015/november/20151130_PR_CHAI_UNITAID
35. Dutertre M, Cuzin L, Puglièse P, Joly V, Valantin M, Cotte L, et al. Initiation of ART based on integrase inhibitors increases the risk of IRIS. CROI 2017, 13-16 February 2017, Seattle Abstract Number: 732
36. Wijting I, Rokx C, Wit F, Postma A, Hoepelman A, van der Ende I, et al. Integrase inhibitors are an independent risk factor for IRIS: an ATHENA cohort study. CROI 2017, 13-16 February 2017, Seattle Abstract Number: 731
37. Duterte M, Cuzin L, Demonchy E, Pugliese P, Joly V, Valantin M-A. Initiation of antiretroviral therapy containing integrase inhibitors increases the risk of IRIS requiring hospitalization. JAIDS 2017 DOI: 10.1097/QAI.0000000000001397

Supplementary Figure 1. Illustration of the model the effect of ART.

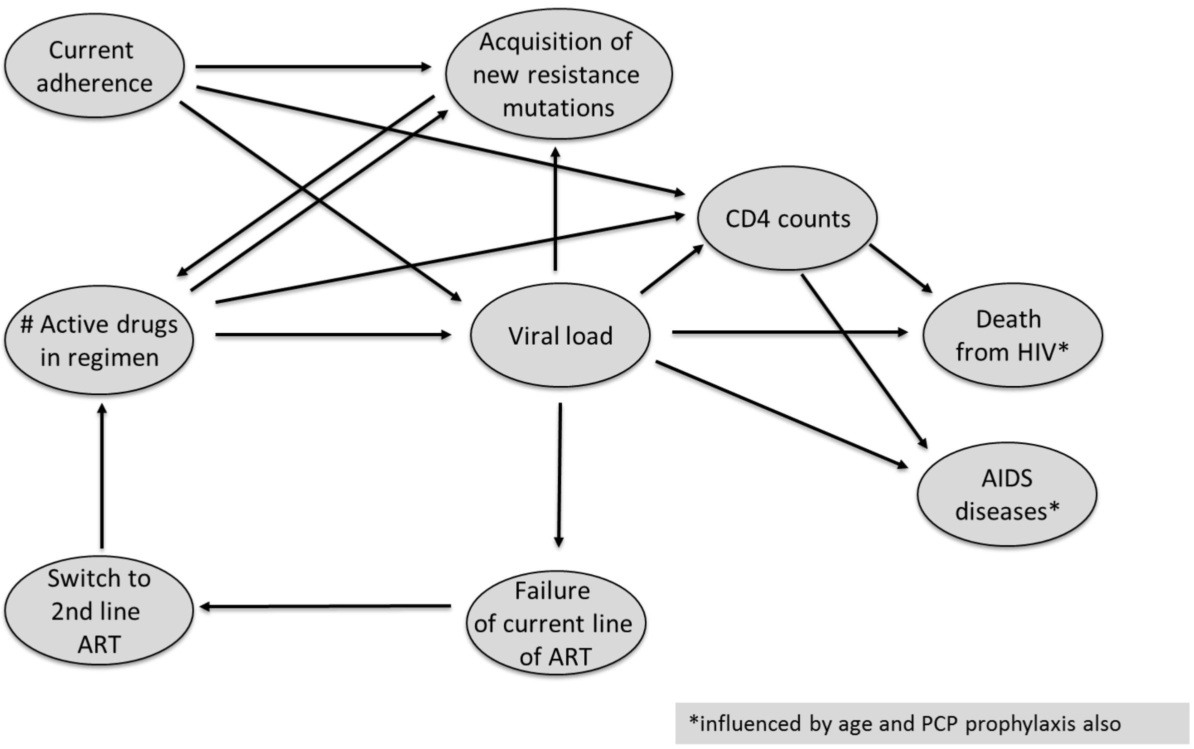

Supplementary Figure 2. Illustration of assumptions on effectiveness of dolutegravir compared with efavirenz (in absence of switching to 2nd line)

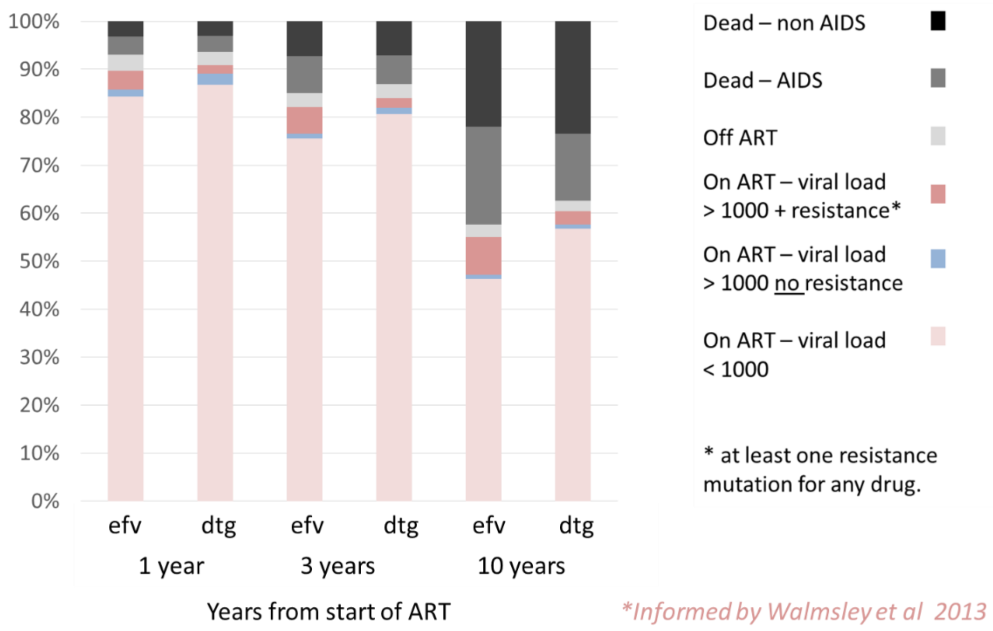

Supplementary Figure 3. Difference in net DALYs compared with no change in policy, according to % of ART initiators in 2017 have NNRTI resistance (mean difference in net DALYs over setting scenarios). Net DALYS take into account DALYs and costs simultaneously (net DALYs = DALYS + cost / cost-effectiveness threshold). The strategy with the lowest net DALYs is the most cost-effective.

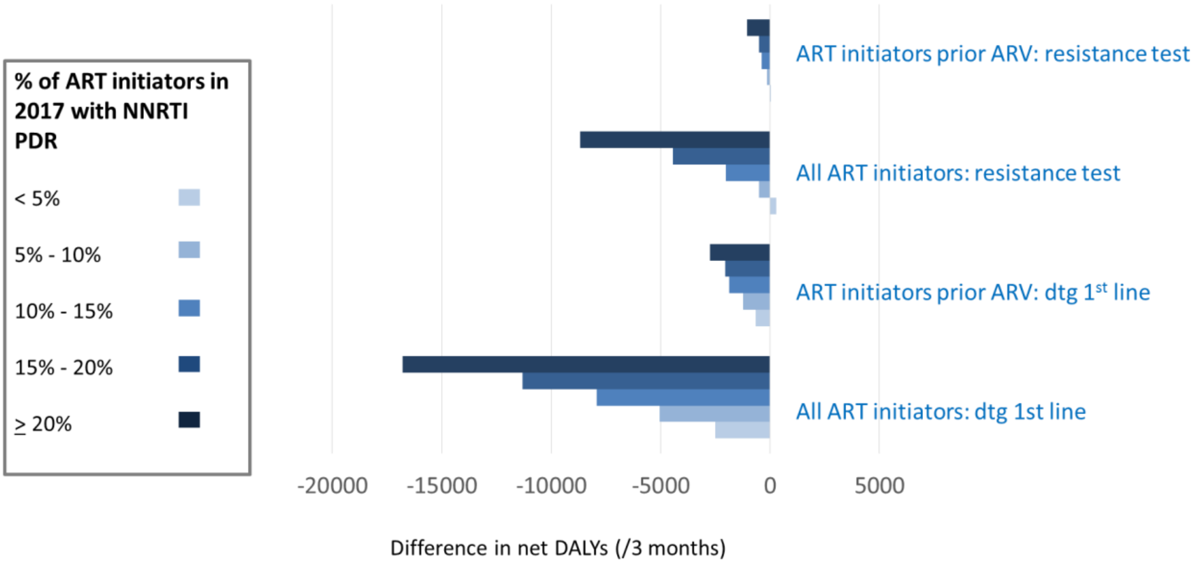

Supplementary Figure 4. Most cost-effective policy according to results from pre-treatment drug resistance surveys

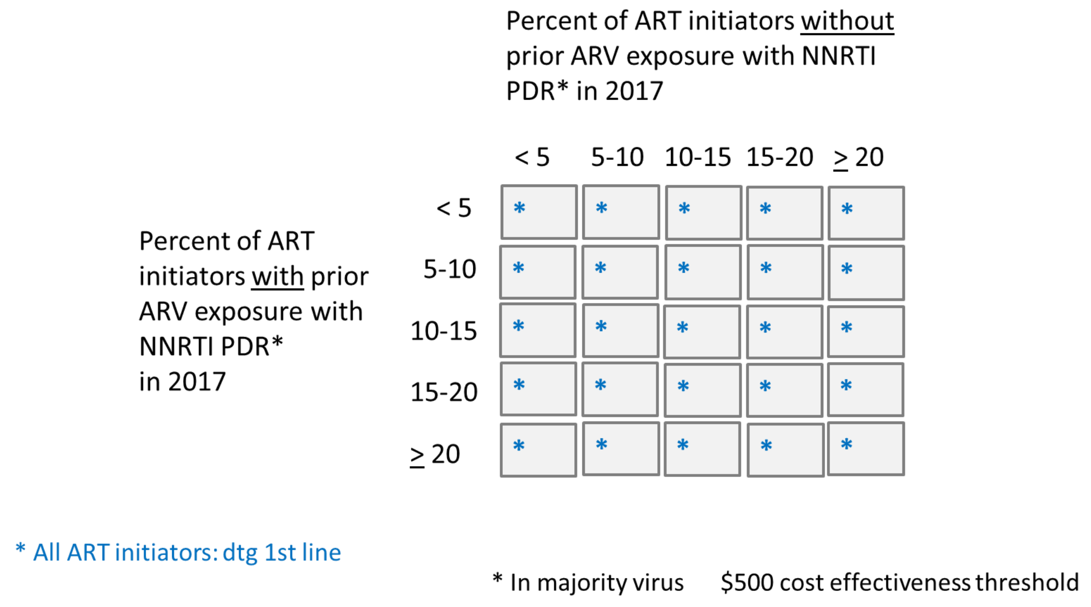

Supplementary Figure 5. Sensitivity analysis with worst plausible case for dolutegravir. Increment in cost and DALYs averted for each policy option, relative to no change in policy. Setting scenarios where > 10% of ART initiators have NNRTI resistance in 2017.

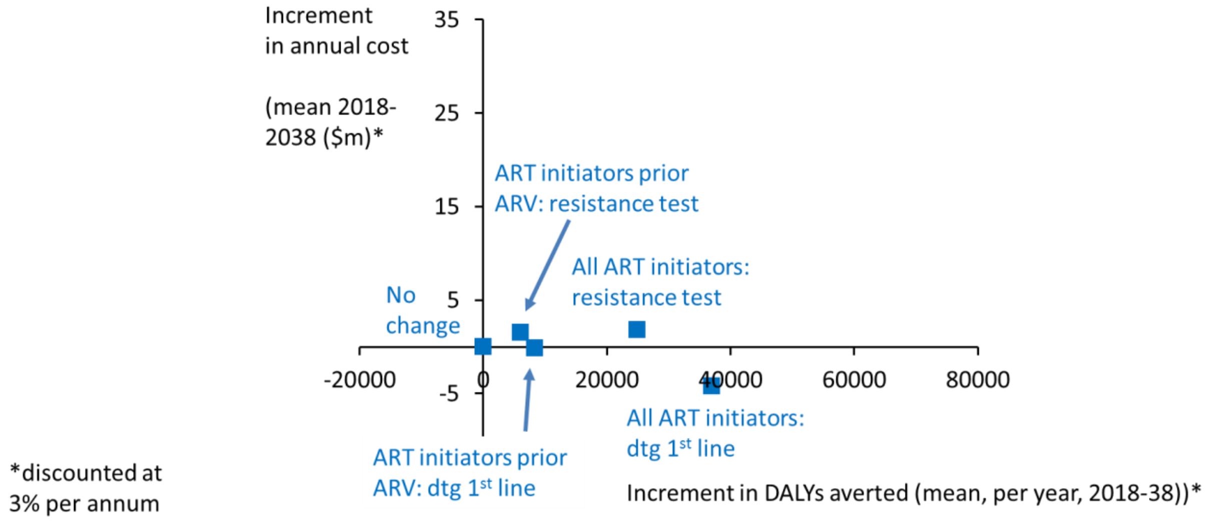

Supplementary Table 1. HIV epidemic and programmatic characteristics of setting scenarios in 2017

	**Median; 90% range over setting scenarios**	**Examples of data from settings**
HIV prevalence (age 15-49)	11% (6% - 22%)	Zimbabwe (2015) 14% (2016) 14%, Tanzania (2011) 5%, Uganda (2011) 9%, Lesotho (2014)25% [1-5]
HIV incidence age 15-49 (/100 person years)	0.72 (0.15 – 1.95)	MPHIA (0.37%), ZAMPHIA (0.66%), ZIMPHIA (0.45%), Justman (2.4%), Huerga (0.39%) [1,6,7]
Proportion of HIV-positive people diagnosed	79% (60% - 90%)	MPHIA (73%), ZAMPHIA (67%), ZIMPHIA (74%), Huerga (75%), Maman (77%) (see also Kim et al, which suggests undisclosed diagnosed HIV) [1, 6, 7, 8]
Proportion of all HIV-positive people on ART	63% (44% - 80%)	ZAMPHIA (57%), MPHIA (64%), ZIMPHIA (64%), Maman (68%), Huerga (52%) [1, 7, 8]
Proportion of ART-experienced people who have started 2nd line (boosted PI) ART	4% (0.5% - 13%)	MoH Malawi (1.5%), 3% [10]
Of people on ART, proportion with VL < 1000	83% (71% - 88%)	South Africa (60%-88% over districts), ZAMPHIA (89%), MPHIA (91%), ZIMPHIA (87%), Maman (91%), Huerga (90%), [1, 7, 8, 11]
Of ART-naïve ART initiators % with NNRTI PDR<br>- in majority virus<br>- in minority or majority virus	10% (1% - 34%)<br>12% (2% - 38%)	Angola (14%), Botswana (8%), South Africa (14%) [12-14]
% of ART initiators with prior ARV exposure<br>Of ART initiators with prior ARV exposure, % with NNRTI resistance in majority virus	18% (8% - 35%)<br>12% (4% - 26%)	Likely to depends on context of discontinuation and re-initiation, which is rarely recorded.

References

1. Justman JE, Hoos D, Kalton G, Nyirenda R, Moyo C, Mugurungi O, Voetsch A, Patel H, Porter L. Real progress in the HIV epidemic: PHIA findings from Zimbabwe, Malawi and Zambia. Conference on Retroviruses and Opportunistic Infections. Feb 2017. Abstract Number: 114LB. http://www.croiwebcasts.org/console/player/33590?mediaType=audio&
2. 2015 Demographic and Health Survey HIV Fact Sheet. Zimbabwe http://dhsprogram.com/pubs/pdf/HF58/HF58.pdf
3. 2011 Demographic and Health Survey Porgram. AIS Tanzania. http://dhsprogram.com/pubs/pdf/AIS11/AIS11.pd
4. 2014. DHS Lesotho http://dhsprogram.com/publications/publication-HF57-HIV-Fact-Sheets.cfm
5. 2011. Uganda AIDS impact survery. http://dhsprogram.com/pubs/pdf/HF41/HF41.pdf
6. Justman J, Reed JB, Bicego G, Donnell D, Li K, Bock N, Koler A, et al. Swaziland HIV Incidence Measurement Survey (SHIMS): a prospective national cohort study. Lancet HIV 2016 http://dx.doi.org/10.1016/S2352-3018(16)30190-4
7. Huerga H. MSF. Mbongolwane and Eshowe HIV Impact in Population Survey http://www.epicentre.msf.org/en/mbongolwane-and-eshowe-hiv-impact-population-survey-0
8. Maman D, Chilima B, Masiku C, Ayouba A, Masson S, Szumilin E, et al. Closer to 90:90:90. The cascade of care after 10 years of ART scale-up in rural Malawi: a population study. JIAS 2016 19:20673 http://dx.doi.org/10.7448/IAS.19.1.20673
9. Kim AA , Mukui I, Young PW, Mirjahangir J, Mwanyumbad S, Wamicwe J, et al. Undisclosed HIV infection and antiretroviral therapy use in the Kenya AIDS indicator survey 2012: relevance to national targets for HIV diagnosis and treatment. AIDS 2016, 30:2685–2695.
10. Haas AD, Keiser O, Balestre E, Brown S, Bissagnene E, Chimbetete C, et al. Monitoring and switching of fi rst-line antiretroviral therapy in adult treatment cohorts in sub-Saharan Africa: collaborative analysis. Lancet HIV 2015; 2: e271–78
11. World Bank. 2015. Analysis of Big Data for Better Targeting of ART Adherence Strategies : Spatial Clustering Analysis of Viral Load Suppression by South African Province, District, Sub-District and Facility (April 2014-March

2015). World Bank, Washington, DC. © World Bank. https://openknowledge.worldbank.org/handle/10986/25399 License: CC BY 3.0 IGO.

12. Afonso JM, Bello G, Guimarães ML, Sojka M, Morgado MG. HIV-1 genetic diversity and transmitted drug resistance mutations among patients from the North, Central and South regions of Angola. PLoS ONE. 2012;7(8):e42996.
13. Rowley CF, MacLeod IJ, Maruapula D, Lekoko B, Gaseitsiwe S, Mine M et al. Sharp increase in rates of HIV transmitted drug resistance at antenatal clinics in Botswana demonstrates the need for routine surveillance. J Antimicrob Chemother. 2016;71(5):1361–6.
14. National Institute for Communicable Diseases, Division of the National Health Laboratory Service. Prospective sentinel surveillance of human immunodeficiency virus related drug resistance. Communicable Disease Communiqué. 2016 March; 15:10-11. http://nicd.ac.za/assets/files/NICD%20Communicable%20Diseases%20Communique_Mar2016_final.pdf

Supplementary Table 2. 95% confidence intervals for mean annual cost over 2018-2038 according to policy option. This relates to Figure 2 in main paper.

	**95% confidence intervals**									
	**No change**		ART initiators prior ARV: resistance test		All ART initiators: resistance test		ART initiators prior ARV: dtg 1st line		All ART initiators: dtg 1st line	
	**13.85**	14.22	13.84	14.22	13.84	14.21	13.85	14.22	13.84	14.21
	**73.56**	76.02	74.4	76.88	77.6	80.19	74.85	77.34	80.92	83.6
	**31.18**	33.38	30.79	32.96	25.87	27.66	30.31	32.45	22.57	24.11
	**36.75**	37.98	36.8	38.04	35.81	37.00	36.77	38.00	35.51	36.67
	**17.32**	17.93	16.82	17.40	14.82	15.32	16.54	17.12	13.45	13.90
	**0.71**	0.75	0.71	0.74	0.69	0.72	0.71	0.74	0.67	0.71
	**13.85**	14.3	14.01	14.47	14.91	15.4	14.16	14.62	15.83	16.36
	**0.22**	0.23	0.21	0.23	0.16	0.17	0.21	0.22	0.12	0.13
	**0.36**	0.37	0.35	0.36	0.26	0.27	0.34	0.36	0.22	0.23
	**0**	0	0	0	0	0	0.005	0.005	0.005	0.005
	**0**	0	1.2	1.28	5.6	5.82	0	0	0	0

HIV tests
1st ine ARVs
2nd line ARVs
clinic visits (non-ARV program costs)
treatment and care for WHO stage 3 and 4 conditions
CD4 tests
VL tests
switching costs
enhanced adherence counselling
regimen transition cost
resistance test costs

Supplementary Table 3. Total cost ($m per year), Total DALYs per year, increment in cost and DALYs compared with no change in policy. Means over 2018-2038.

	Total Cost ($m per year, mean 2018-2038)	Total DALYs ('000 per year, mean 2018-2038)*	Increment in cost ($m per year, mean)	Increment in DALYs ('000 per year, mean)*	Incremental net DALYs, compared to no change (mean, per year)**
No change	191.5	2,715	0	0	0
ART initiators prior ARV: resistance test	192.9	2,709	+1.4	-6	-2,857
All ART initiators: resistance test	193.2	2,689	+1.7	-26	-22,249
ART initiators prior ARV: dtg 1st line	191.4	2,706	-0.1	-9	-9,190
All ART initiators: dtg 1st line	186.5	2,674	-5.0	-41	-50,669

*Since DALY-weighs are applied to years of life-lived, total DALYs should not be misinterpreted as representing burden of disease, but are instead only a means to calculate incremental DALYs.

** Incremental net DALYs shows the reduction in population burden of disease, measured in DALYs, per year of each policy compared to no change. The policy which reduces population burden of disease the most (i.e. All ART initiators: dtg 1st line) is cost-effective.

2. Modelling details

2.1. Introduction to the approach taken

The HIV Synthesis Transmission model is an individual-based stochastic model of heterosexual transmission, progression and treatment of HIV infection within a southern African context (Phillips et al 2011, Cambiano et al 2013, Cambiano et al 2014). Details of the model are given in subsequent sections below. For this project we based the demographics of the population studied and HIV epidemic features around those for Malawi, although by sampling widely from the parameter distributions we generated diverse setting scenarios in respect of many epidemic aspects, such as sexual behaviour, HIV prevalence, ART uptake and HIV incidence. The parameter distributions are described in section 2.9 below.

2.2. Demographic model

General population death rates and determination of age in 1989

The model runs to from 1989 (the start of the epidemic) to 2039 (although in our results we concentrate on the period to 2038), with variables updated in 3 month periods. Each run of the simulation program creates 100,000 simulated people who will be age 15 or above at some point between 1989 and 2039, of whom approximately 35,000 are alive and age over 15 at any one point in time. In order to scale up from the simulated population to a population size of 10 million we use a scale factor of 260.

The initial age distribution for both males and females is determined on the basis of the distribution in Table S1.

Table S1. Distribution of ages of simulated individuals in 1989

Age group	Probability of being in age group in 1989
-35-14	0.770
15-24	0.083
25-34	0.065
35-44	0.041
45-54	0.025
55-64	0.015

This distribution is chosen such that in the absence of HIV, given the death rates below, the population size increases over time, as is projected in Malawi and other countries in the region. Thus around 77% of simulated people have an age below 15 in 1989 (and most are yet to be born). The only variable that is modelled and updated up to reaching the age of 15 (when becoming potentially sexually active) is age itself. The “youngest” person in 1989 is age -35 (i.e. will be born in 2024 and reach age 15 in 2039, when the modelled period ends.

Age specific death rates for uninfected people are based on death rates in South Africa in 1997 (Table S2) – before the significant impact of HIV-related deaths.

Table S2. Age specific death rates (per year)

Age group	Annual death rate
Males	
15-19	0.00200
20-24	0.00320
25-29	0.00580
30-34	0.00750
35-39	0.00800
40-44	0.01000
45-49	0.01200
50-54	0.01900
55-59	0.02500
60-64	0.03500
65-69	0.04500
70-74	0.05500
75-79	0.06500
80-84	0.10000
≥85	0.40000
Females	
15-19	0.00150
20-24	0.00280
25-29	0.00400
30-34	0.00400
35-39	0.00420
40-44	0.00550
45-49	0.00750
50-54	0.01100
55-59	0.02000
60-64	0.02100
65-69	0.04000
70-74	0.03800
75-79	0.05000
80-84	0.07000
≥85	0.15000

For the context of Malawi, these death rate are multiplied by 2 based on fitting to the population pyramid https://www.cia.gov/library/publications/resources/the-world-factbook/geos/mi.html (people and society).

2.3. Sexual behaviour and risk of HIV acquisition

Here we describe the approach to modelling sexual behaviour and HIV acquisition. The basic approach is summarized in Figure S1. The parameter values related to sexual behaviour were chosen such that they lead to a modelled HIV prevalence level over time as observed. Sexual behaviour is characterized by two variables representing, respectively, the number of short term condomless sex partners and whether the person has a current long term condomless *sex* partners in the 3 month period. The status of long term partners is tracked over time (i.e. if they are infected, diagnosed, on ART). Short term partners are not tracked over time, in that if

a person has a short term partner in time period t who is infected with HIV, this is independent of the probability that any short term partner in time t+1 is infected with HIV.

Figure S1. Summary of modelling of sexual behaviour and HIV acquisition

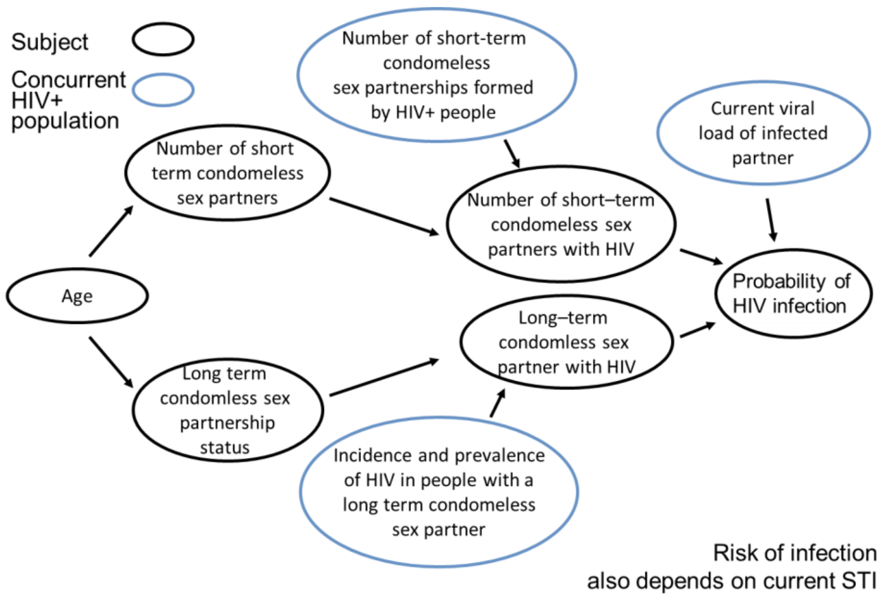

Determination of number of short term (condomless sex) partners at period t

Numbers of short term partners in a given period was generated at random, according to which of four sexual behaviour groups the person was in for this period; (i) no short term condomless partners in 3 month period, (ii) 1 short term partner, (iii) medium number of short term partners, and (iv) high number of short term partners. Changes in the sexual behaviour group from t-1 to t were determined by transition probabilities between the 4 groups. Transition probabilities p_{gija} of moving from partner group i at t-1 to partner group j at t are given by

$$p_{gija} = \frac{f_{gij} \times r_{ga}}{\left(f_{gi1} + \sum_{j=2}^{4}(f_{gij} \cdot r_{ga})\right)}$$

where g = 0,1 for males, females, respectively, and a = 1-10 for age groups 15-, 20-, 25-, 30-, 35-, 40-, 45-, 50-, 55-, 60-, respectively. Values of f_{gij} and r_{ga} are given in Tables S3 and S4, respectively, and if j=1 then r_{ga}=1.

We are not aware of the existence of data on intra-person variability in the number of condomless sex partners. To considered two sets of values of f_{gij} (A and B) as shown in Table S3, characterized by substantially different intra-person variability over time in sexual behaviour subgroup. We considered that version B is more likely to be closer to reality mainly because it leads to a lower proportion of men and women with high numbers of condomless partners, more in keeping with data from the DHS (Malawi Demographic and Health Survey), albeit that the DHS data are not restricted to condomless sex. So, as explained below in Table S16, for 90% of model runs we assumed version B and for the other 10% version A.

Values of r_{ga} are modified at time t by a factor 0.2 if the subject has a current AIDS defining disease and by a factor *ch_risk_diag_newp* (with a value 0.83, informed by (Fonner et al 2012) if the subject is diagnosed with HIV (sqrt(*ch_risk_diag_newp* from 6 months after diagnosis). In addition, there is a person-fixed modification factor. For a proportion *p_rred_p* of men and 1.5.*p_rred_p* of women, values of r_{ga} are modified by a factor 0.1, to reflect the fact that a proportion of people experience only very low sexual risk activity in their life. Similarly for a proportion *p_hsb_p* values of r_{ga} are modified by a factor 3 in women, representing women with a higher chance of becoming sex workers. The value of these parameters is sampled from a distribution at the start of each model run (see below).

Actual transitions between groups were determined by random sampling. For the first two groups the number of partners in the period is given (i.e. no short term partners, 1 short term partner, respectively). When a person was in the medium short term partners group the number of partners was determined by sampling from a Poisson(*highsa*). When in the high short term partners group the number of partners was determined by sampling from a Poisson(2) distribution and multiplied by the parameter *swn*. The value of these parameters *highsa* and *swn* are also sampled from a distribution at the start of each model run (see below).

Table S3. Values of f_{gij} (values determining probability of transitioning between short term partner risk behaviour groups). Sexual behaviour transition matrices A and B are characterized by substantially different intra-person variability over time in sexual behaviour subgroup. We consider that version B is more likely to be closer to reality mainly because it leads to a lower proportion of men and women with high numbers of condomless partners, more in keeping with data from the DHS (Malawi Demographic and Health Survey), albeit that the DHS data are not restricted to condomless sex. As explained below in Table S16, for 90% of model runs we assumed version B and for the other 10% version A.

Sexual behaviour transition matrix A

Short term partners group in period t-1	Short term partners group in period t<br>0	 1	 medium (Poisson mean *highsa)*	 high (Poisson mean 2 x *swn)*
**Males**				
0	0.89	0.08	0.03	0.00
1	0.80	0.15	0.05	0.00
medium	0.35	0.27	0.38	0.00
high	0.20	0.30	0.50	0.00
**Females**				
0	0.93	0.05	0.02	0.00025
1	0.86	0.11	0.03	0.0005
medium	0.54	0.08	0.38	0.001
high	0.05	0.05	0.10	0.800

Sexual behaviour transition matrix B

Short term partners group in period t-1	Short term partners group in period t<br>0	 1	 medium (Poisson mean *highsa)*	 high (Poisson mean 2 x *swn)*
**Males**				
0	0.98	0.01	0.01	0.00
1	0.90	0.05	0.05	0.00
medium	0.35	0.27	0.38	0.00
high	---	---	---	---
**Females**				
0	0.99	0.005	0.005	0.001
1	0.95	0.04	0.01	0.001
medium	0.025	0.02	0.95	0.005
high	0.03	0.01	0.03	0.930

Table S4. Values of r_{ga} (factor determining relative level of sexual risk activity). These values are broadly informed by age specific self-reported number of partners and HIV prevalence.

Age group (a=1,10)	Males (g=1)	females (g=2)
15-	0.60	1.60
20-	0.60	1.60
25-	1.00	1.00
30-	0.80	0.80
35-	0.65	0.50
40-	0.50	0.35
45-	0.40	0.10
50-	0.35	0.05
55-	0.25	0.04
60-	0.15	0.02

Determination of having a long term (condomless sex) partner at period t

Only condomless sex partnerships are modelled. Thus if a person has a long term partner but condoms are used on all occasions of sexual intercourse then this is not counted as having a long term condomless sex partner.

At each period, people with no current long term partner have age-dependent probabilities of having a new long term partner is dependent on parameter *eprate* and given by: age 15-24, p= *eprate*; age 25-34, p= *eprate*; age 35-44, p= *eprate/2*; age 45-54, p= *eprate/3*; age 55-64, p= *eprate/5* (*eprate* = 0.1).

At the time a long term partnership is started, it is classified into 3 duration groups, each with a different tendency to endure. The percent of people in each group is dependent on age and is shown in Table S5.

At time period, t, for people with a long term partner, the probability of the condomless sex partnership continuing is (1-(0.25 / *ch_risk_beh_ep*)) if duration category is 1, is (1-(0.05 / *ch_risk_beh_ep*)) if duration category is 2, and (1-(0.02 / *ch_risk_beh_ep*)) if duration category is 3, where *ch_risk_beh_ep* is a parameter conveying the population level change in sexual behaviour with long term partners that occurs in 1995. Further, this probability is reduced by a factor *ch_risk_diag* in the 3 month period after a partner's diagnosis, if a partner has HIV and is diagnosed. The value of these parameters is sampled from a distribution at the start of each model run (see section 2.9). These values mean that, for example, the mean duration of the most recent period of condomless sex with a long term partner in 2017 is 2.5-3 years.

Note also that levels of sexual behaviour, in terms of numbers of short term partners and the probability of a long term partner are essentially determined by the levels of such sexual behaviour required in order to produce an epidemic as observed, given rates of transmission with condomless sex partners. Sexual behaviour tends to be under-reported, particularly in women, and higher levels of behaviour have to be assumed both to be consistent with levels of risk behaviour reported in men, and to generate an epidemic of the proportions observed (e.g. Gregson 20022, Johnson 2009).

Table S5. Percent of newly formed long term partnerships classified into each of three duration groups, each of which has a different tendency to endure (higher class, more durable). These values mean that, for example, the mean duration of the most recent period of condomless sex with a long term partner in 2017 is 2.5-3.0 years.

Age	1	2	3
15-44	30%	30%	40%
45-54	30%	50%	20%
55-64	30%	70%	0%

Population level change in sexual behaviour

There is assumed to be a general average reduction in condomless sex after 1995, reflecting the reductions observed over the period from around this date (Gregson 2010, Halperin 2011).

Determination of number of short term (condomless sex) partners who are HIV infected at time t

For each short term partner that a subject has at time t, the probability that the partner is infected is calculated. This is dependent on the prevalence of HIV in those of the opposite gender, taking consideration of age mixing. If the subject is of gender g and age group a, then for each short term partner the first step is to determine by sampling at random, the age group of the short term partner, a^{newp} (in fact, for simplicity, all short term partners at time t are assumed to be in this same age group). The gender and age mixing probabilities used are given by values in Table S6.

Table S6. Sexual mixing by age and gender. The proportion of short term partnerships formed by men in age group a_m which are with females of age group a_f and the proportion of short term partnerships formed by females in age group a_f which are with men of age group a_m. These values are informed by knowledge that partnerships involving an older man and younger woman are much more common than partnerships involving an older woman and younger man.

	Females Age group (a_f)				
Males Age group (a_m)	15-24	25-34	35-44	45-54	55-64
15-24	0.865	0.11	0.025	0.00	0.00
25-34	0.47	0.43	0.10	0.00	0.00
35-44	0.30	0.50	0.20	0.00	0.00
45-54	0.43	0.30	0.23	0.03	0.01
55-64	0.18	0.18	0.27	0.27	0.10

	Males Age group (a_m)				
Females Age group (a_f)	15-24	25-34	35-44	45-54	55-64
15-24	0.43	0.34	0.12	0.10	0.01
25-34	0.09	0.49	0.30	0.10	0.02
35-44	0.03	0.25	0.34	0.25	0.13
45-54	0.00	0.00	0.05	0.25	0.70
55-64	0.00	0.00	0.00	0.10	0.90

Then, for the given partner (of gender 1-g and age group a^{newp}), the risk that the partner is infected is then given by

$$h_{gat} = \frac{\sum_{a^{\text{newp}},(1-g)} L_{(t-1)}^{\text{inf}}}{\sum_{a^{\text{newp}},(1-g)} L_{(t-1)}}$$

where $L_{(t-1)}^{\text{inf}}$ is the total number of infected short term partners at time (t-1), and $L_{(t-1)}$ is the total number of short term partners at time t-1. The numerator is therefore the total number of infected short term partnerships of the opposite gender in age group a^{newp}.

Since we assume that all short term partners at time t are in this same age group, the total number of infected short term partners that the subject has at time t, L_t^{inf}, is then given by

$$L_t^{\text{inf}} = \text{Min}(\text{Poisson}(h_t \cdot L_t), L_t)$$

Determination of probability that a long term partner is HIV infected at time t

E_t^{inf} indicates whether the subject has a long term (condomless sex) partner who is infected ($E_t^{\text{inf}} = 1$ if infected, else $E_t^{\text{inf}} = 0$). A long term partner at time t can be infected either because (i) a new long term partnership has been formed and the partner was already infected, (ii) because a long term partner at t-1, which has remained a long term partner at time t, has become infected, or (iii) because an infected long term partner has remained as a long term partner.

For (i):

$E_t^{\text{inf}} = 1$ if $L_{(t-1)}^{\text{inf}} \geq 1$ (If a person starts to have a new long term condomless sex partnership in the same period as they have a new short term condomless sex partner we assume that these two partners are the same and hence if the new short term partner has HIV then this is carried through to the new long term partner).

For (ii):

The probability that a long term partner of a subject of age group a and gender g becomes infected is derived from the HIV incidence at t-1 for age group a (i.e. the same age group) and gender 1-g, $i_{a(1-g)(t-1)}$ among the sexually active population, either with a long term partner or at least one short term partner (which is given by the number of subjects newly infected in age group at time *t-1* divided by the number of HIV-uninfected subjects in age group at *t-1*, who had condom-less relationships, either long or short term)

$$\begin{cases} E_t^{\text{inf}} = 1, & U < i_{a(1-g)(t-1)} \text{ where } U \text{ randomly sampled from } Uniform(0,1) \\ E_t^{\text{inf}} = 0, & \text{otherwise} \end{cases}$$

In order to maintain balance, for each gender, between the number of uninfected people with a long term partner who is infected, and the number of infected people with a long term partner who is uninfected, this incidence $i_{a(1-g)(t-1)}$ is modified at time t dependent on the degree of balance at time t-1.

For (iii):

If $E_{(t-1)}^{\text{inf}} = 1$ and $E_t \geq 1$ then assign $E_t^{\text{inf}} = 1$

Determination of the risk of infection from a short term partner

For each HIV infected short term partner of a subject of gender g and age group a the viral load group, v, of the partner is obtained by sampling from the viral load distribution of those of the opposite gender. Thus we sample from Uniform(0,1), where the probability of the partner having viral load in group v is given by

$$\frac{\sum_v L_{(t-1)}^{\text{inf}}}{\sum L_{(t-1)}^{\text{inf}}}$$

where the numerator is the total number of short-term partnerships had by infected people in viral load group v and the denominator is the total number of short-term partnerships had by infected people (in any viral load group).

Viral load groups are:

(1) < 2.7 log cps/mL
(2) 2.7-3.7 log cps/mL
(3) 3.7-4.7 log cps/mL
(4) 4.7-5.7 log cps/mL
(5) $\geq$ 5.7 log cps/mL
(6) primary infection.

Once the viral load group, v, of the infected partner is determined, the probability, t_v, of the subject being infected by the partner is then given according to: t_1 = Normal (*tr_rate_undetec_vl*,0.000025^2), t_2 = Normal (0.01,0.0025^2), t_3 = Normal (0.03,0.0075^2), t_4 = Normal (0.06,0.015^2), t_5 = Normal (0.1,0.025^2), t_6 = Normal (*tr_rate_primary*,0.075^2). These are based on Hollingsworth et al (2008) and Bellan (2015) and are the rates for a longer term partner. The transmission rate for a short term partner is multipled *by fold_tr_newp* (0.35) due to the assumed lower number of sex acts. These probabilities are increased by *fold_change_w*-fold (= 1.5) for female subjects aged $\geq$ 20, by 2-fold for female subjects aged < 20, and by *fold_change_sti*-fold (= 3.0) if the person has an existing STI (risk of a new STI in any one three month period is given by the number of short term condomless partners / 20 (or 1 if > 20 short term partners)) (Cohen et al 1998, Nicolosia 1994).

We assume that super-infection can occur (i.e. a person can be re-infected with HIV with consequent risk of acquiring new mutations).

Realization of whether the subject is infected by each short term partner is determined by sampling from Uniform(0,1).

Determination of the risk of infection from a long term partner

Infected long term partners at time t are classified by whether they are in primary infection (if infection occurred at t-1), whether they are diagnosed with HIV, whether they are on ART, and whether their current viral load is < 2.7 cps/mL or not. The proportion of long term partners with HIV who have HIV diagnosed at time t, $p_t^{\text{e,diag}}$, is determined with reference to the difference, $d_{(t-1)}^{\text{e,diag}}$, in the proportion of subjects with HIV who are diagnosed, $\frac{T_{(t-1)}^{\text{diag}}}{T_{(t-1)}^{\text{inf}}}$ and $p_{(t-1)}^{\text{e,diag}}$;

i.e. $d_{(t-1)}^{\text{e,diag}} = \frac{T_{(t-1)}^{\text{diag}}}{T_{(t-1)}^{\text{inf}}} - p_{(t-1)}^{\text{e,diag}}$

where $T_{(t-1)}^{\text{diag}}$ is the total number of subjects diagnosed with HIV at time t-1and $T_{(t-1)}^{\text{inf}}$ is the total number of subjects with HIV (diagnosed and undiagnosed) at time t-1.

$$\begin{cases} \text{if } 0 < d^{\text{e,diag}}_{(t-1)} \leq 0.05 \text{ then } p^{\text{e,diag}}_t = 0.4 \\ \text{if } 0.05 < d^{\text{e,diag}}_{(t-1)} \leq 0.10 \text{ then } p^{\text{e,diag}}_t = 0.5 \\ \text{if } 0.10 < d^{\text{e,diag}}_{(t-1)} \leq 0.15 \text{ then } p^{\text{e,diag}}_t = 0.9 \\ \text{if } 0.15 < d^{\text{e,diag}}_{(t-1)} \text{ then } p^{\text{e,diag}}_t = 0.95 \end{cases}$$

The proportion of those diagnosed who are on ART, and the proportion of those on ART who have viral load < 2.7 log cps/mL are determined in a similar manner. In this way the proportions diagnosed with HIV, on ART, and with current viral load is < 2.7 log cps/mL are kept similar for the long term partners as in the simulated subjects themselves.

Risk of infection from a long term infected partner is determined by Normal (*tr_rate_primary*, 0.075^2) if the existing partner is in primary infection (i.e. infected at t-1), Normal (*tr_rate_undetec_vl*, 0.000025^2) if the existing partner has viral load < 2.7 log cps/mL, and Normal (0.05, 0.0125^2) otherwise.

Transmitted resistance

The modelling of transmission of drug resistance is summarized in Figure S2. The presence or not of resistance mutations does not influence the risk of transmission (i.e. virus with resistance mutations present is assumed equally transmissible as virus without such mutations, for a given viral load). Resistance is modelled in terms of the presence or absence of mutations specific to the drugs in use. Distinction is made for each mutation as to whether it is only present in minority virus (if the patient has a mutation present but has stopped drugs that select for that mutation), so the mutation is assumed not transmissible, or if it is present in majority virus, and hence the mutation is assumed transmissible. The probability that resistance mutations present in majority virus of the source partner are transmitted to the newly infected person is dependent on the specific mutation. Once a resistance mutation is transmitted to the new host it is assumed to have a certain probability of being lost from majority virus over time (Jain et al JID 2011; Castro et al JID 2013; Yang et al PLOS Pathogens 2015). Even after being lost from majority virus, it is assumed to remain in minority virus and is selected back as majority virus if an antiretroviral drug selecting for that mutation is initiated. We also consider the possibility of a person who is already infected become super-infected, including with drug resistant HIV (Smith 2005), although there is assumed to be at most a 20% chance that a person super-infected by a person with HIV resistance then has virus with those resistance mutations as a result.

Figure S2. Overview of modelling of transmission of drug resistance.

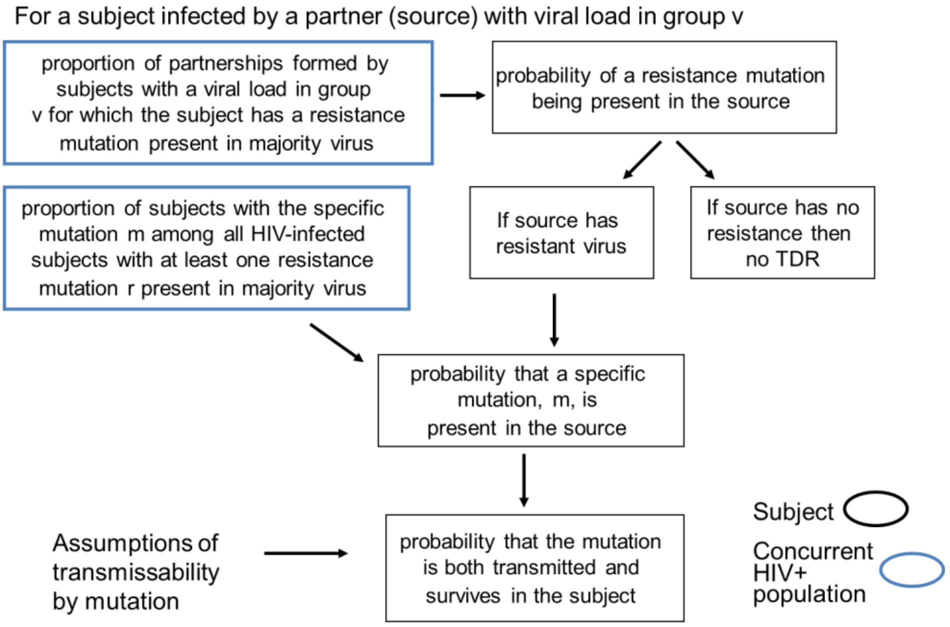

Transmitted resistance: details

The viral load group of the person who infected the subject is known, as indicated above. For a subject infected by a person in viral load group v the probability of a resistance mutation being present in the infected person is given by

$$\frac{\sum_{v,\text{ and mutation present}} L_{(t-1)}^{\text{inf}}}{\sum_{v} L_{(t-1)}^{\text{inf}}}$$

where $\sum_{v,\text{ and mutation present}}$ is the sum over all partnerships had by HIV-infected people in viral load group v for whom a resistance mutation is present in majority virus and $\sum_{v}$ is the sum over all HIV-infected subjects in viral load group v. Again, realization of whether the subject is infected by a person with at least one resistance mutation in majority virus is determined by sampling from Uniform(0,1).

For subjects infected from a source partner with a resistance mutation, the probability that a specific mutation, m, is present in the source is given by

$$\frac{\sum_{\text{mutation } m \text{ present}} L_{(t-1)}^{\text{inf}}}{\sum_{\text{mutation present } v} L_{(t-1)}^{\text{inf}}}$$

Where $\sum_{\text{mutation } m \text{ present}}$ is the sum over all HIV-infected subjects with mutation m present in majority virus and $\sum_{\text{mutation present}}$ is the sum over all HIV-infected subjects with at least one resistance mutation in majority virus.

If a given resistance mutation, m, is present in the source partner, the probability that the mutation is both transmitted and survives in the subject (i.e. that its presence will affect future response to drugs for which the mutation confers reduced sensitivity) is mutation specific.

We consider uncertainty in the extent to which transmitted resistance mutations are effectively immediately lost (even from minority virus) by sampling from a distribution for parameter *res_trans_factor*, which is a parameter sampled at the start of each run.

Loss from majority virus of transmitted mutations

There is a probability per 3 months of loss of persistence of transmitted mutations from majority virus to minority virus (same for each mutation) *rate_loss_persistence,* which is one of the parameters sampled at the start of each model run.

2.4. Natural history of HIV infection

Figure S3 gives an overview of the modelling of HIV natural history. The model of the natural history of HIV and the effect of antiretroviral therapy has been derived previously and compared with a range of observed data (see Phillips et al Lancet 2008, AIDS 2011, Nakagawa et al 2012, 2015 and associated supplementary material). Below we set out the structure of the model and explain what parameters represent.

Figure S3. Overview of modelling of natural history of HIV infection.

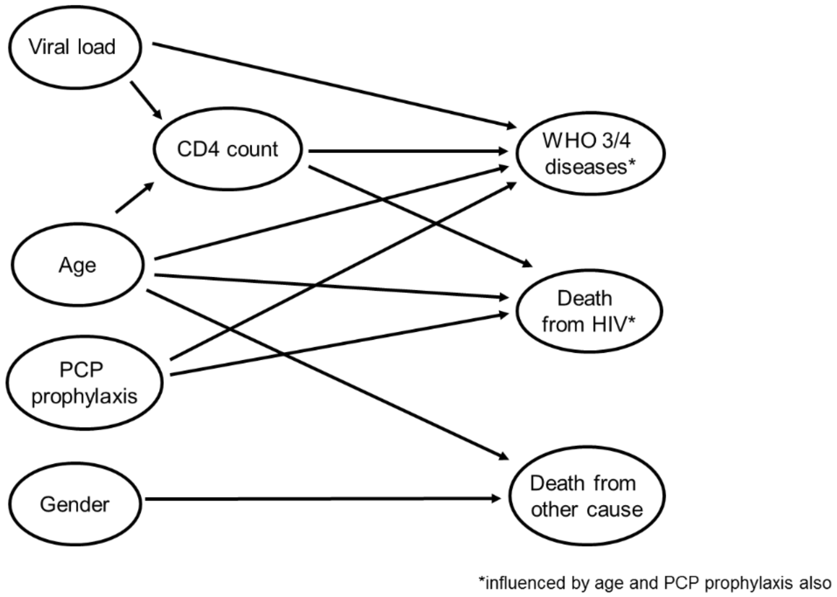

Determination of changes in viral load and CD4 count

Initial $\log_{10}$ viral load (V_{set}) is sampled from Normal(4.0,0.5^2)

This viral load (V_{set}) is assumed to be that reached after primary infection. It is not used to determine the risk of transmission in primary infection itself.

Initial CD4 count, modelled on the square root scale, is partially dependent on initial viral load and given by

Square root CD4 count = *mean_sqrtcd4_inf* (= 27.5) - (1.5 x V_{set}) + Normal(0,2^2) – ((age – 35) x 0.05)

Initial virus is assumed to be R5-tropic. Shift to presence of X4 virus is assumed to depend on viral load. Probability of a shift per 3 months is given by 10^v x 0.0000004, where v is the current $\log_{10}$ viral load.

Viral load change (vc) from period t-1 to period t (i.e. in 3 months) is given by

vc(t-1) = (gx x 0.02275 + Normal(0, 0.05^2) + ((age(t-1) - 35) x 0.00075)

gx=1 viral load at t (v(t)) = v(t-1)+ vc(t-1)

CD4 count changes from period t-1 to t are dependent on the current viral load (i.e. viral load at time t-1) and are given by sampling from a Normal distribution with standard deviation *sd_cd4* and mean *fx* (= 1.0) times the values as follows:

Viral load at t-1	Change in square root CD4 count (per 3 mths)
< 3.0	+0.000
3-0	- 0.022
3.5-	- 0.085
4.0-	- 0.400
4.5-	- 0.400
5.0-	- 0.850
5.5-	- 1.300
6.0-	- 1.750

The change additionally is affected by the current age as follows:

People with X4 virus present experience an additional change in square root CD4 count of -0.25.

These estimates were derived based on consideration of evidence from natural history studies (Pantazis 2005, Sabin JAIDS 2000, Hubert J-B 2000, O'Brien 1998, Henrard 1995, Lyles 2000, Touloumi 2004, Mellors 1997, Koot 1993) and were selected in conjunction with other relevant parameter values to provide a good fit to the incubation period distribution. Differences that have been found in initial viral load by sex, age and risk group are not currently incorporated in the model.

Table S7. Example model outputs of incubation period by age. Kaplan-Meier percent with WHO 4 Event. Compare with Darby et al 1996. This varies by model run due to the sampling of the value of the parameter *fx* Described above.

Age at infection	Years from infection					
	1	3	5	10	15	20
15-	0.6%	4%	14%	50%	75%	89%
25-	1.1%	2%	23%	67%	88%	97%
35-	2.1%	13%	34%	82%	97%	100%
45-	3.7%	21%	54%	93%	100%	100%
55-	1.4%	24%	59%	96%	100%	100%

2.5. HIV testing and diagnosis of HIV infection

HIV testing was assumed first introduced in 1996. At that time we assumed 20% of the population were resistant to be tested for HIV unless symptomatic and this decreased linearly to 5% by the end of 2010. In the model this group has no possibility of getting tested for HIV unless symptomatic. Limited data are available to inform this parameter (proxy variables are the proportion who reported never being tested for HIV and, more precisely, the proportion who refuse HIV testing), nevertheless we considered it important to take this into account, given the evidence that not everyone accepts HIV testing for various reasons. The level of acceptability of provider initiated HIV testing and counselling (PITC) in resource limited settings is extremely variable from levels of 99%, observed in inpatients in Uganda (Wanyenze 2011) to 31% among outpatients in South Africa (Bassett 2007). Among pregnant women the level of acceptability of PITC seems to be higher, varying from 76 to 99.9% (Hensen

2012), while the estimated acceptability of home-based counselling and testing has been estimated in a meta-analysis to be 83% (Sabapathy 2012). This variability seems to be related mainly to the quality of the intervention delivered and calendar time. Acceptability seems to have increased over time due to the reduction in stigma and higher availability of ART, therefore we thought it was reasonable to assume a decline in the proportion resistant to be tested for HIV down to 5% in 2010.

For the remainder of the population (non-resistant to HIV testing), increasing gender and age-specific rates of HIV testing (for the 1st time and for repeat testing) since 1996 were assumed (parameter *an_lin_incr_test* sampled at the start of each model run), to reflect the range of levels of testing observed in Malawi and other countries in the region. This increase in testing is assumed to stop so that testing rates reach a plateau (*date_test_rate_plateau*). We assume some targeting of testing such that those having a condomless sex partner since last test are more likely to test – the degree of such targeting is conveyed by the parameter *test_targeting*. Pregnant women experience an additional probability of being tested in the ANC, which increases over calendar time (*rate_testanc_inc*).

People with acute symptoms (WHO stage 4, 3 or active TB) are assumed to have a higher chance of testing for HIV in that 3 month period and a higher chance of being linked to care once diagnosed and the increase over time in this testing probability *incr_test_rate_sympt*. Like all other parameters mentioned in this section this is sampled at the start of each model run (see section 2.9).

2.6. Modelling the effect of ART

The structure of the relationship between ART adherence, viral load, development of resistance, CD4 count and risk of death is modelled is illustrated in Figure S4 below. The adherence level - the determination of which is described in detail below - influences the risk of acquisition of new mutations as well as having a direct effect on the viral load and CD4 count. Acquisition of resistance mutations impacts on the number of fully active drugs in the current regimen. This, in turn, is a further determinant of the risk of new mutations arising. Failure of the current line of ART is determined by CD4 count or viral load or clinical disease, depending on the monitoring strategy, and this triggers a switch to the next line of ART (if assumed available, and often with a delay), which leads to the number of active drugs again returning to 3 or more if on boosted PIs. The following sections provide further details, including how adherence levels are determined and how they influence the viral load, risk of resistance and the CD4 count. We also explain the modelling of ART interruption and loss to follow-up. We provide references to papers that have been used to inform the approach. It should be noted though that parameter values used in the model are rarely extracted directly from any one paper, they are values that are arrived at based on their ability to generally reproduce outputs that are consistent with observed estimates, as illustrated below.

Figure S4. Overview of the modelling of the effect of ART, highlighting the role of adherence.

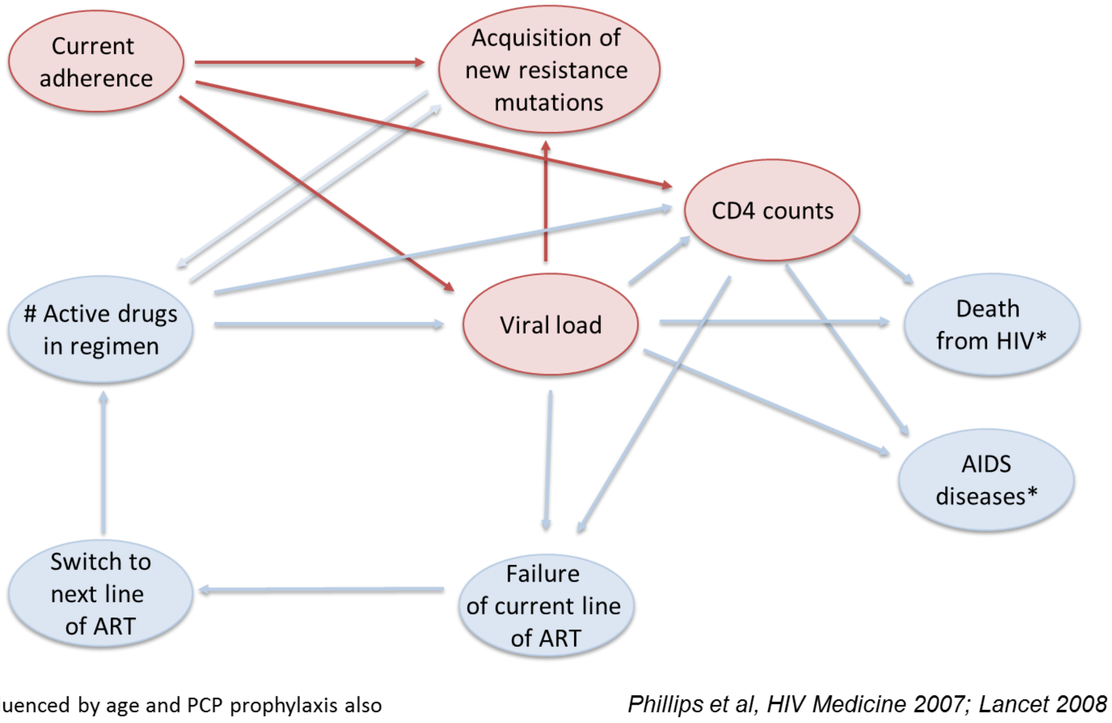

Initiation of ART

ART initiation in diagnosed people before 2010 is determined by a measured CD4 count < 200 or the development of a WHO 4 event. From 2011 this is determined by a CD4 count < 350 and from 2015 by a CD4 count < 500 or pregnancy (option B+). We assume that CD4 counts are monitored at 6 monthly intervals for those who are in pre-ART care.

Switch to second line after failure of first line ART

Whichever the criterion for the need to switch to second line ART is determined, the probability of switching per 3 month period after the criterion is met is *pr_switch*_line . The value of this parameter is sampled from a distribution at the start of each model run (see below). The switch rate is likely to vary substantially by setting (Fox 2012; Johnston 2012). In several settings, including Zimbabwe, the proportion of people who have started second line ART is consistent with a value for *pr_switch*_line of below 0.1 (e.g. Lesotho, Malawi) (personal communications Zimbabwe MoHCC; Government of Malawi Ministry of Health, Integrated HIV Program Report, Oct-Dec 2014).

Adherence pattern

The model specifies a current adherence level (i.e. for the current 3 month period) for people on ART. Given that the model updates in 3 month time periods the adherence level in a given 3 month period has to effectively be considered as the average adherence over the period. The determination of this is described below. Interruption of ART for periods of duration 3 months is considered separately (and explained in subsequent sections below).

Consistent with evidence that people tend to have different tendencies to adhere (Cambiano 2010a;Carrieri 2001;El-Khatib 2011;Genberg 2012;Glass 2010;Kleeberger 2004;Lazo 2007;Levine 2005;Mannheimer 2002;Meresse 2014;Osterberg 2005), adherence is modelled using two components. Each patient has a certain

greater or lesser tendency to adhere (*adhav,* measured on a scale of 0-100%) but their actual adherence in a given period varies over time, both at random and according to the presence of symptoms (with drug toxicity or presence of WHO stage 4 disease leading to a decrease in adherence) and there is an effect of a tendency for increasing adherence with age. Adherence in a given 3 month period (*adh{t}*) is measured on a scale of 0 to 100%. *adhvar* is the standard deviation representing the within-person period-to-period variability over time. Thus, adherence at any one period (*adh{t}*) is determined as follows (although with modifications explained below):- adh(t) = *adhav* + Normal(0,$adhvar^2$). The distribution of the values of *adhav* and *adhvar* is specified as follows and as illustrated in Figure S5:

5% probability	*adhav* = 10% *adhvar* = 0.2
10% probability	*adhav* = 80% *adhvar* = 0.2
65% probability	*adhav* = 90% *adhvar* = 0.05
20% probability	*adhav* = 95% *adhvar* = 0.05

Figure S5. Illustration of adherence pattern assumptions. 5% of the population have the adherence as shown in the top left, 10% as shown in the top right, etc. While adherence is generally high in the majority of people on ART (hence the high proportion of people on ART with viral suppression), most probably experience at least some periods of poorer adherence (e.g. see (Muyingo 2008)).

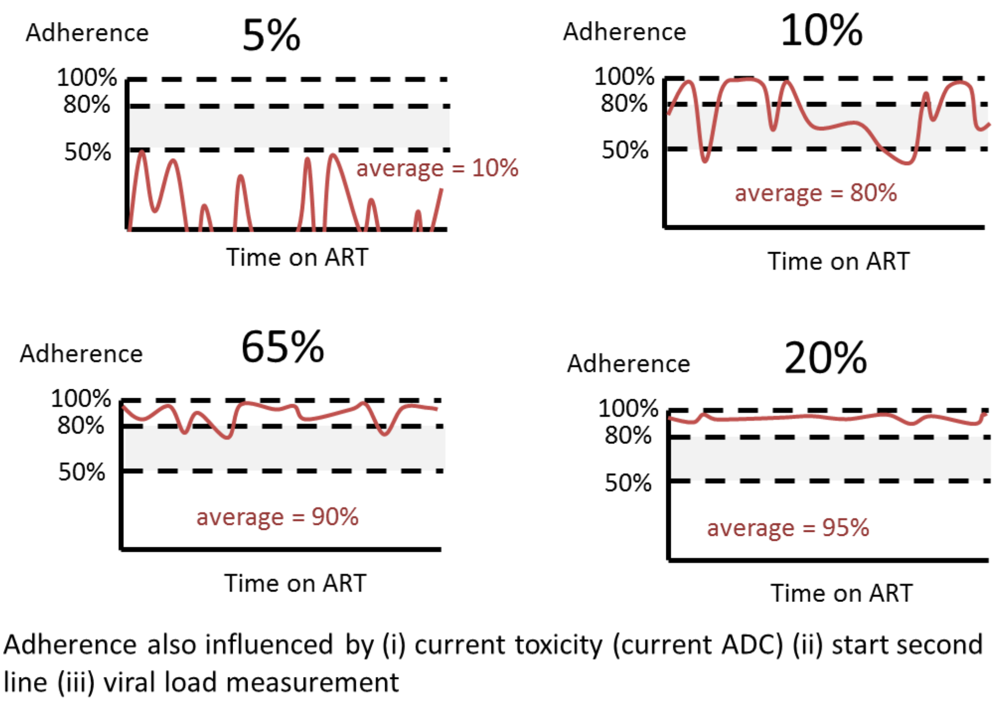

This distribution of adherence is primarily determined by the adherence levels required for the model outputs to mimic observed data. This includes data on rates of resistance development and virologic failure and also data on the proportion of patients at first virologic failure who have no resistance mutations present (Bangsberg 2004;Bangsberg 2006b;Hamers 2011;Hassan 2014;Hoffmann 2014;Kobin 2011;Li 2014;Mackie 2010;Mannheimer 2002;Meresse 2014;Rosenblum 2009;Tran 2014;Usitalo 2014;von, V 2013). It is clear from such data in more recent years that the great majority of patients who started ART with 3 or more drugs are sufficiently adherent that virologic failure rates are low (and so resistance accumulation is also likely to be low) (El-Khatib 2011;Johannessen 2009).

Figure S6: Risk of virologic failure while on ART according to adherence level

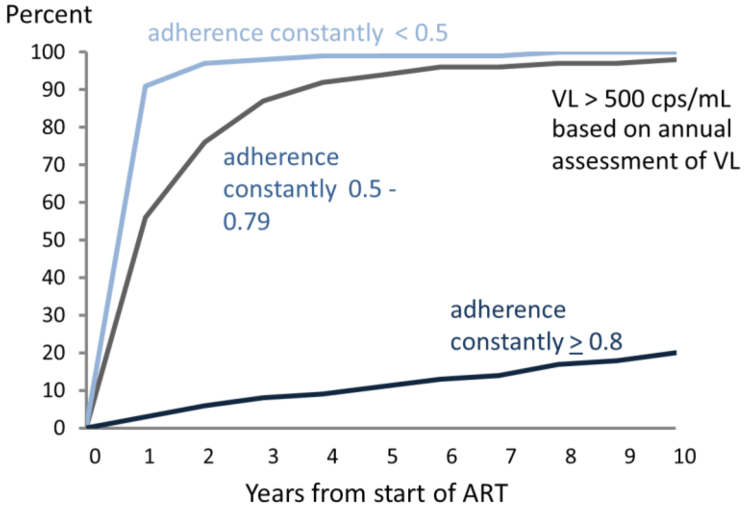

Figure S7: Risk of NNRTI resistance with virologic failure while on ART, according to adherence level

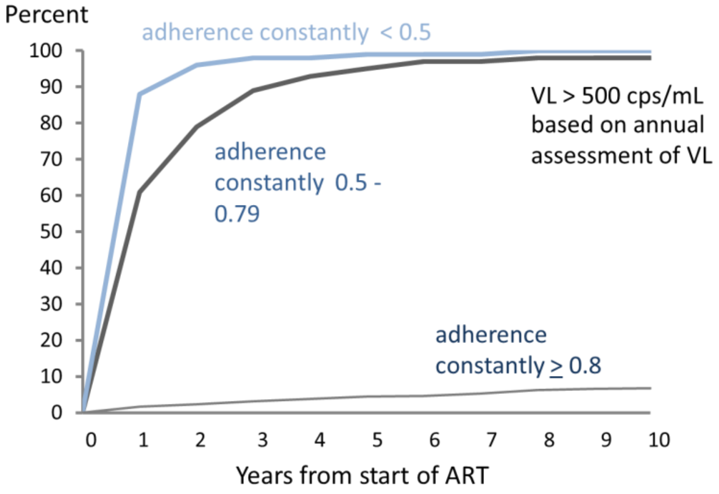

The distribution of adherence over the first year of ART has been compared with data from a large programme in Zambia (see Figure S8; (Chi 2009)). Observed data and model outputs on viral load at one year from start of ART is shown in Figure S9. These are reconstructed outcomes for all people who have initiated ART in Zimbabwe (the overall mean CD4 count at initiation is 145 /mm^3). Figures S10 and S11 compare Kaplan Meier estimates of time to virologic failure and resistance, respectively, between the model and observed data, in the latter case from the UK due to relative the lack of data from sub-Saharan Africa (but note for example Wadonda-Kabondo N et al 2012, Stadeli et al 2013). Figure S12 illustrates the proportion of people with resistance (amongst those on ART with non-suppressed viral load) and corresponds to estimates from the large WHO resistance surveillance.

Figure S8. Distribution of average adherence level over first year of ART (for those on ART at 1 year).

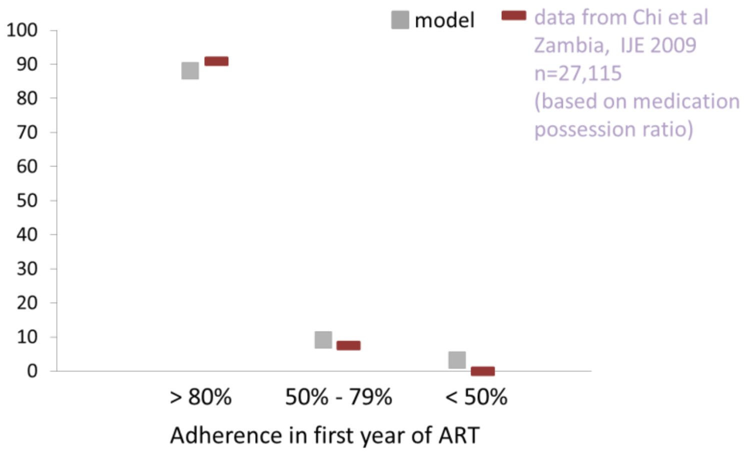

Figure S9. (a) Percent of people alive at given time points from start of ART who have viral load suppression and (b) percent of people alive and on ART at given time points .from start of ART who have viral load suppression (WHO Resistance Surveillance Report 2012(WHO 2012)).

(a)

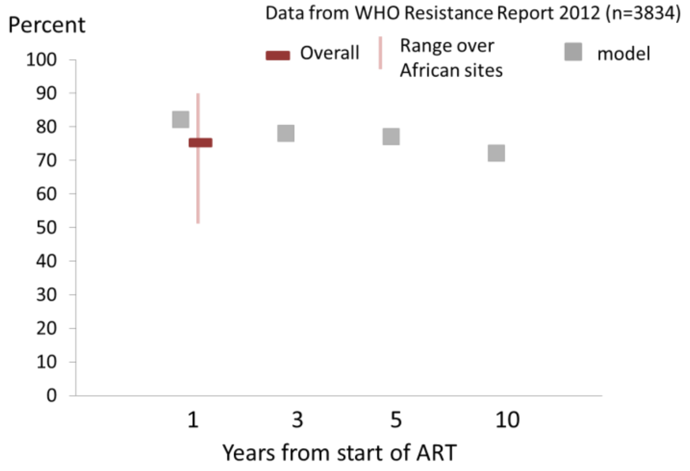

(b)

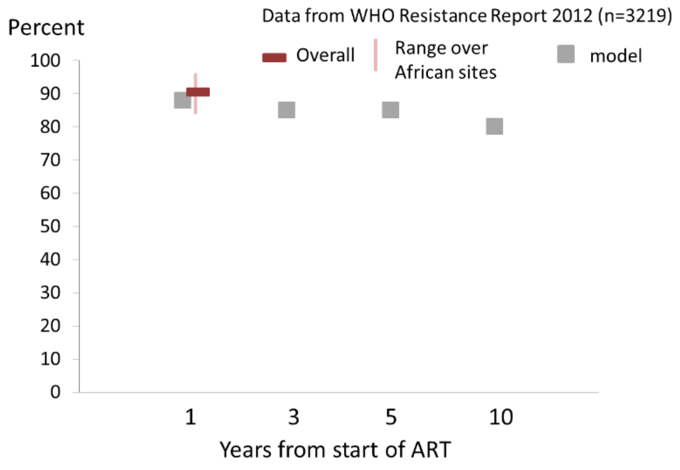

Figure S10. Kaplan Meier estimates of risk of virologic failure while on ART, by time from start of ART.

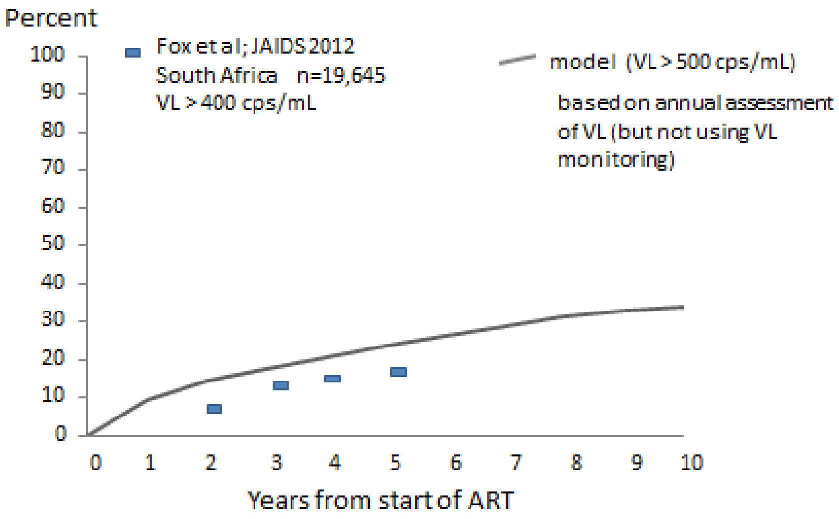

Figure S11. Kaplan Meier estimates of risk of NNRTI resistance with virologic failure while on ART, by time from start of ART (Cozzi-Lepri 2010).

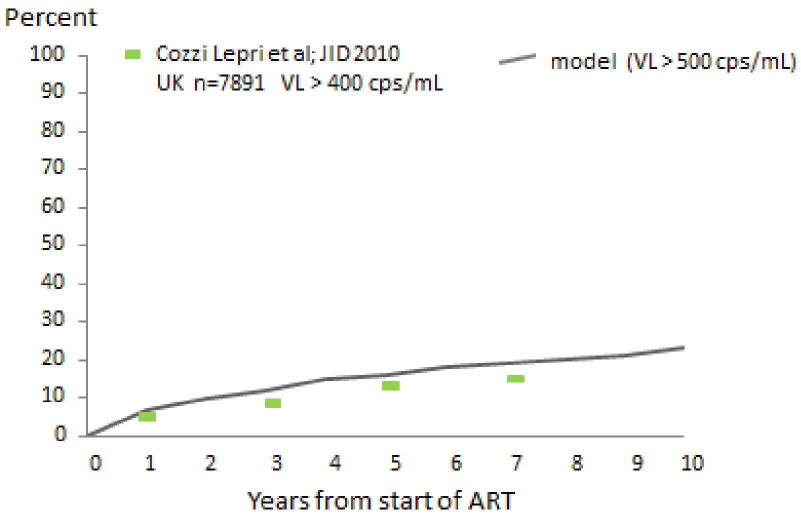

Figure S12. Of people with viral load > 500 at 1 year from start of ART, percent who have NNRTI drug resistance (WHO Resistance Surveillance Report 2012(WHO 2012)).

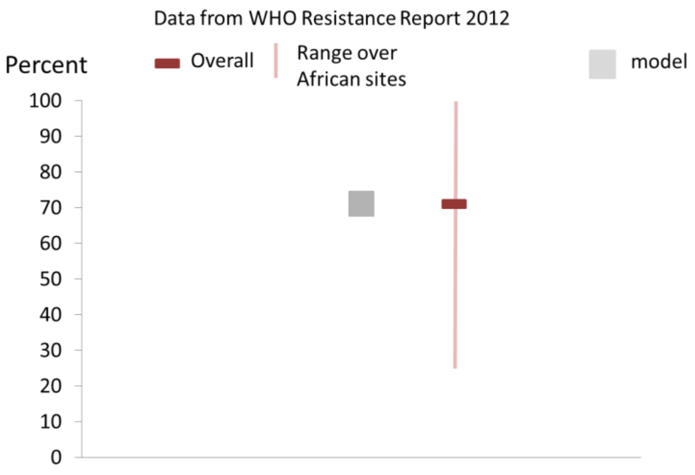

Sampling to determine adherence pattern

Since we are considering a range of plausible scenarios other distributions of adherence values are also considered besides the one described above and we sampled from the following alternative adherence profiles at the start of each model run.

A

1% probability	*adhav* = 10%	*adhvar* = 0.2
1% probability	*adhav* = 80%	*adhvar* = 0.2
18% probability	*adhav* = 95%	*adhvar* = 0.05
80% probability	*adhav* = 95%	*adhvar* = 0.02

B

3% probability	*adhav* = 10%	*adhvar* = 0.2
2% probability	*adhav* = 80%	*adhvar* = 0.2
15% probability	*adhav* = 95%	*adhvar* = 0.05
80% probability	*adhav* = 95%	*adhvar* = 0.02

C

3% probability	*adhav* = 10%	*adhvar* = 0.2
3% probability	*adhav* = 80%	*adhvar* = 0.2
14% probability	*adhav* = 90%	*adhvar* = 0.05
80% probability	*adhav* = 95%	*adhvar* = 0.05

D

5% probability	*adhav* = 10%	*adhvar* = 0.2
7% probability	*adhav* = 80%	*adhvar* = 0.2
8% probability	*adhav* = 90%	*adhvar* = 0.05
80% probability	*adhav* = 95%	*adhvar* = 0.05

E

5% probability	*adhav* = 10%	*adhvar* = 0.2
10% probability	*adhav* = 80%	*adhvar* = 0.2
85% probability	*adhav* = 95%	*adhvar* = 0.05

F

5% probability	*adhav* = 10%	*adhvar* = 0.2
10% probability	*adhav* = 80%	*adhvar* = 0.2
65% probability	*adhav* = 90%	*adhvar* = 0.05
20% probability	*adhav* = 95%	*adhvar* = 0.05

H

15% probability	*adhav* = 10%	*adhvar* = 0.2
15% probability	*adhav* = 80%	*adhvar* = 0.2
50% probability	*adhav* = 90%	*adhvar* = 0.05
20% probability	*adhav* = 95%	*adhvar* = 0.05

I

20% probability	*adhav* = 10%	*adhvar* = 0.2
20% probability	*adhav* = 80%	*adhvar* = 0.2
40% probability	*adhav* = 90%	*adhvar* = 0.05
20% probability	*adhav* = 95%	*adhvar* = 0.05

J

30% probability	*adhav* = 10%	*adhvar* = 0.2
30% probability	*adhav* = 60%	*adhvar* = 0.2
10% probability	*adhav* = 70%	*adhvar* = 0.05
30% probability	*adhav* = 90%	*adhvar* = 0.05

adh_pattern 100 a; 99 b; 1 c; 105 d; 98 e; 2 f; 3 g; 4 h; 5 i

Effective adherence

We also included the concept of *effective* adherence, which reflects predicted adequacy of drug levels, whereby for those on regimens that do not include an NNRTI the effective adherence is as the adherence itself, but for those on NNRTI-containing regimens the effective adherence is the adherence + *add_eff_adh_nnrti* (base value 0.1), reflecting the long half-life of NNRTI drugs (Cheeseman 1993) which is an advantage as it means such regimens are more forgiving of periods of poor adherence (Bangsberg 2004;Bangsberg 2006a;Bangsberg 2006b;Gardner 2009;Gross 2008;Kobin 2011;Meresse 2014;Parienti 2007). Additionally, it is assumed that patients on ART are susceptible to occasional (rate 0.02 per year) severe temporary drops in drug level (i.e. effective adherence level), leaving them susceptible to viral rebound (but with low risk of resistance as the effective adherence drop is so profound). This phenomenon is assumed to be 3 times more frequent among those on protease inhibitor regimens than in those on other regimens. This latter assumption is the only plausible means (at least within our model framework) to explain why virologic failure occurring on boosted protease inhibitor regimens often occurs in the absence of resistance (Hill 2013).

Figure S13. Status at 1 year from start of ART. Example of model output. Data is from WHO Drug Resistance Surveillance Report (2012).

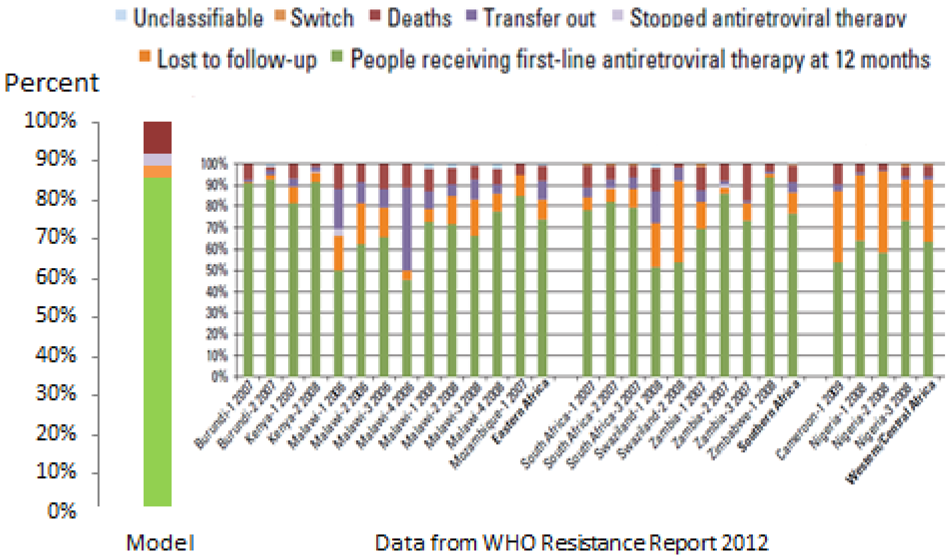

Effect of viral load measurement above 1000 cps/mL on adherence

Various factors can influence adherence, including the initial measurement of viral load > 1000 copies/mL which is assumed to lead to an increase in adherence in 70% of people as a result of targeted adherence intervention; this is consistent with data showing that a high proportion of people with measured viral load > 1000 copies/mL who undergo an adherence intervention subsequently achieve viral suppression without a change in ART (Orrell et al 2007, Hoffman et al 2009, Hoffman et al 2013, Rutstein et al 2015) and broadly consistent with a meta-analysis (Bonner et al 2013). Although the appropriate duration to assume for this effect is uncertain (Hoffman et al 2013), the impact of adherence interventions has often been shown to diminish with time (Bärnighausen et al 2011). Based on this overall body of data, we assume that the adherence intervention is effective only the first time it is performed and that for 40% the effect is permanent (i.e. 70% x 40%= 28% of those with a viral load >1000), but that in the remaining 60% (i.e. 70% x 60% = 42% of those with viral load>1000) it lasts only 6 months.

Interruption of ART

People can interrupt ART, and this may be due to not continuing with clinic visits (disengagement, modelled as simultaneous interruption and loss) but ART can be interrupted also in those still attending clinical visits. The basic rate of interruption due to patient choice is *rate_int_choice* (sampled at the start of each model run, see below for distribution from which sampled) - this rate is greater in people with current toxicity (2-fold) (note that in addition to this increased risk of interruption with current toxicity, there is assumed to be some substitution of drugs causing toxicity with available alternatives and a greater rate of interruption in patients with a greater tendency to be non-adherent (1.5-fold if adherence average *adhav* 50 – 79% and 2-fold if adherence average *adhav* < 50%). In a systematic review, drug toxicity, adverse events and side effects have been found to be the most commonly given reasons for drug discontinuation (Kranzer 2011).

The rate of interruption also reduces with time on ART, decreasing after 2 years. Evidence suggests that rates of discontinuation does decrease over time ((Kranzer 2010;Tassie 2010;Wandeler 2012) although the point at which the risk lowers might be somewhat earlier than 2 years. If adherence average (*adhav*) $\geq$ 80% then there is a 30% chance that interruption coincides with interrupting/stopping visits to the clinic, if 50 <= *adhav* < 80% then 45% chance, if *adhav* < 50% then 60% chance. This is due to an assumption that factors leading to poor adherence are also likely to be associated with interruption. The rate of interruption and disengagement with care is likely to vary by setting. Figure S14 shows a comparison between modelled and observed (from a study by Kranzer et al. (Kranzer 2010)). Kaplan Meier estimates of the percent of people having interrupted or discontinued ART by time from ART initiation.

Figure S14. Percent who have interrupted or discontinued ART by time from initiation.

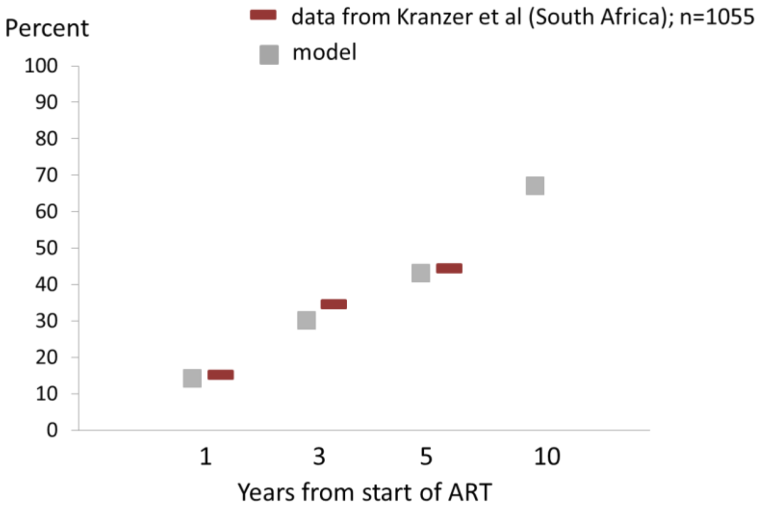

Interruption of ART without clinic/clinician being aware

It is known that in some instances people on ART have such poor adherence that they have in fact interrupted or stopped ART entirely but, in the same way that the clinician is not always aware of the true adherence level, they are also not always aware when the person has completely interrupted ART. This means that the clinician (in the absence of a resistance test) may think a patient is virologically failing, because viral load is high, when in fact this is due to interruption rather than resistance. This can be seen from studies on people with virologic failure in which a proportion have no identified resistance mutations (Hamers 2011;Hoffmann 2009;Wallis 2010). Thus, when a person interrupts ART (but remains under care) we introduce a variable that indicates whether the clinician is unaware. *clinic_not_aw_int_frac* (sampled at the start of each model run, see below for distribution from which sampled). This value of 0.6 was chosen to produce realistic model outputs for the

proportion of people with virological failure who have resistance. If a patient has interrupted ART with the clinician unaware then not only is the patient (wrongly) classified (by the clinician) as virologically failing (if viral load has been measured), but a switch to second line can occur. Figure S13 compares the proportion of people with resistance between the model and WHO survey data.

Re-initiation of ART after interrupting in patients still under clinic follow-up

For patients who have interrupted ART due to choice but are still under clinic follow-up, the probability of restarting ART per 3 months in the base model is *prob_restart* (sampled at the start of each model run, see below for distribution from which sampled). This probability is increased 3-fold if a new WHO 3 condition has occurred at t-1, and 5-fold if a new WHO 4 condition has occurred at t-1 since occurrence of clinical disease in a person seen at clinic is likely to prompt ART re-initiation. This will vary by setting but is informed by studies showing that of people who have initiated ART who are still seen at clinic a very high proportion are on ART at 12 months from start of ART (McMahon 2013). Kranzer et al found a rate of restarting ART amongst those that interrupted or discontinued of 21 per 100 person-years but this figure is an overall figure which includes in the denominator those who are not attending the clinic (loss to follow-up and return to care are described below). The equivalent figure, produced as an output from the model is 19 per 100 person-years.

Interruption due to drug stock-outs

The basic rate of interruption due to interruption of the drug supply is *prob_supply_interrupted* per 3 months (base value: 0.01). The rate of resupply (*prob_supply_resumed*) has a base value 0.8 per 3 months. This will vary by setting. For patients who have interrupted ART due to interruption of supply the probability of restarting ART per 3 months is *prob_supply_resumed* (base value 0.8).

Loss to follow-up while off ART (for reasons apart from drug stock-outs)

The probability per 3 months of interrupting/stopping clinic visits (i.e. being lost to follow-up) is *rate_lost* (sampled at the start of each model run, see below for distribution from which sampled) if adherence average *adhav* $\geq$ 80%. This is increased by 1.5 fold if 50% $\leq$ *adhav* < 80% and by 2-fold if *adhav* < 50%. This high rate is informed by the fact that low numbers of people attending clinics after having been initiated on ART are not still on ART (e.g. WHO 2012 Resistance report). Interruption of ART and loss to follow-up are assumed correlated with the underlying tendency to adhere when on ART because we assume that the same underlying social, practical and economic factors will be an underlying cause of these behaviours.

For people lost to follow-up who are asymptomatic, the probability of returning to clinic per 3 months is *rate_return* (sampled at the start of each model run, see below for distribution from which sampled) if adherence average *adhav* $\geq$ 80%. This is decreased by 2-fold if 50% $\leq$ *adhav* < 80% and by 3-fold if *adhav* < 50%. If a person develops a new WHO 3 or 4 event then they are assumed to return to the clinic with probability 1. As mentioned above, this leads to an overall rate of restarting of ART after interruption (including having been loss to follow-up in many cases) consistent with the estimates from South Africa from Kranzer et al, although these will vary by setting (Charurat 2010;Fox 2012;Kranzer 2010).

As output from the model, the retention on ART at 1 year is 94% amongst those still alive. This is difficult to compare with estimates from the literature because few studies are able to know the outcome status of all people initiated on ART, and a high proportion of those lost from a given clinic in fact remain on ART at another clinic or have died. However, taking this into account, this modelled output value of 94% seems consistent with data from the WHO Drug Resistance Surveillance Report (2012) (see Figure S13).

Effect of ART on viral load, CD4 count, resistance development and drug toxicity

This section describes the determination of updated viral load, CD4 count, and acquisition of new resistance mutations in a given time period for people on ART. The updated viral load, CD4 count and risk of new resistance mutations appearing all depend on the effective adherence in the previous and current period, the number of active drugs (*nactive(t-1)*) and the current viral load, as well as the time period from the last time ART was started or restarted. The values of viral load, CD4 count, and resistance mutation risk for any combination of these factors are given in Table S8 below. The rationale behind this approach and how the specific values in the table were chosen is explained below. The choice of values is directly informed by studies in this area and by comparison of model outputs with data. For the new resistance mutation risk, the number in the table is multiplied by the viral load (mean of values at t-1 and t) to give a value for the variable *newmut*, which is used when assessing whether a new mutation or mutations have arisen (see below).

Number of active drugs

We use the concept of the number of drugs that are active, based on presence of resistance mutations to the drugs being used. The level of resistance is determined by the presence of drug resistance mutations, with a given set of mutations being translated into a level of resistance to a given drug on a scale of 0 to 1 in the same way as is done for common resistance interpretation systems. The activity level of a drug is then calculated as 1 minus the level of resistance to the drug. The ability of the number of active drugs, or the genotypic sensitivity score, to predict the viral load outcome is well established (DeGruttola 2000), and the concept of using a genotypic score to define "optimised background therapy" has been common to the design of several trials in treatment experienced patients (e.g. (Grinsztejn 2007)).

Classification of adherence levels

While we model the adherence level for each individual at each three month time period as a value between 0 and 100%, to determine the viral load, CD4 count and resistance risk, we classify adherence into three levels. This is the simplest approach that allows inclusion of the fact that the relationship between adherence and resistance risk is not linear, since the risk of resistance tends to be lower when the adherence is either low or high, and the risk of resistance is highest when adherence is moderate, allowing enough replication for mutations to be selected for and enough drug present to allow selection of virus with resistance mutations (Bangsberg 2004;Gardner 2009;Rosenbloom 2012).

The cut-offs used to define the three adherence levels are 50% and 80%. Adherence-resistance and adherence-viral load relationships differ by regimen type and even specific regimen within a class and any overall breakdown into groups is necessarily a simplification. A cut off of 80% is chosen as the upper level as (unlike for unboosted PI regimens) at adherence levels of at least 80%, NNRTI and boosted PI regimens are likely to have maximal or close to maximal effects on viral load and minimal risk of resistance selection (Parienti 2007). Actual risk of resistance probably depends on the pattern of adherence, not just the average over a three month period, so that a treatment interruption of over 1 week during the three month period, while maintaining an overall average adherence of 80%, could lead to a higher level of risk of resistance emergence than a situation in which the adherence was more uniform over the period (Genberg 2012), although in people who have ongoing viral suppression NNRTI regimens seem to be generally robust to even relatively low levels of adherence (Cambiano 2010b;Gross 2008;Meresse 2014;Parienti 2007). A level below 50% is one that that has been associated with raised risk of detectable viral load (Arnsten 2001;Genberg 2012)

Determination of viral load, CD4 count and risk of resistance in people on ART

Viral load, CD4 count and risk of resistance in the first 3 months after (re-)starting ART

Table S8a shows how the viral load, CD4 count and risk of resistance is determined for people in the first 3 months after starting ART or re-starting ART after an interruption of at least 3 months. Since in this early period on ART, the viral load will depend on the initial value the updated viral load is given as a reduction from the pre-

ART maximum viral load. If the number of active drugs is three or more then at a high adherence level (above 0.8) the mean viral load change from the pre-ART maximum is 3 log copies/mL. To reflect the fact that there is variability in the response (Montaner 1998), the value for a given person is sampled from a Normal distribution with standard deviation 0.2. This viral load response diminishes both with decreasing number of active drugs in the regimen being started (which is informed by data from studies relating GSS to virologic outcome, as well as by studies of mono and dual therapy regimens (DeGruttola 2000;Eron 1995;Havlir 1995;Kuritzkes 1996;Larder 1995;Phillips 1997;Wittkop 2011;Wittkop 2013). The viral load response also diminishes with decreasing level of adherence (see Figure S15 and for example Genberg et al). As is well established, the CD4 count response generally mirrors the viral load response, although with very low numbers of active drugs and low adherence there is a mean decrease in CD4 count and still a small decrease in viral load from the maximum.

Figure S15. Model output: of people on ART, percent with current VL >500 according to current adherence. Comparison with data from Genberg at el on electronic monitoring-based adherence measures.

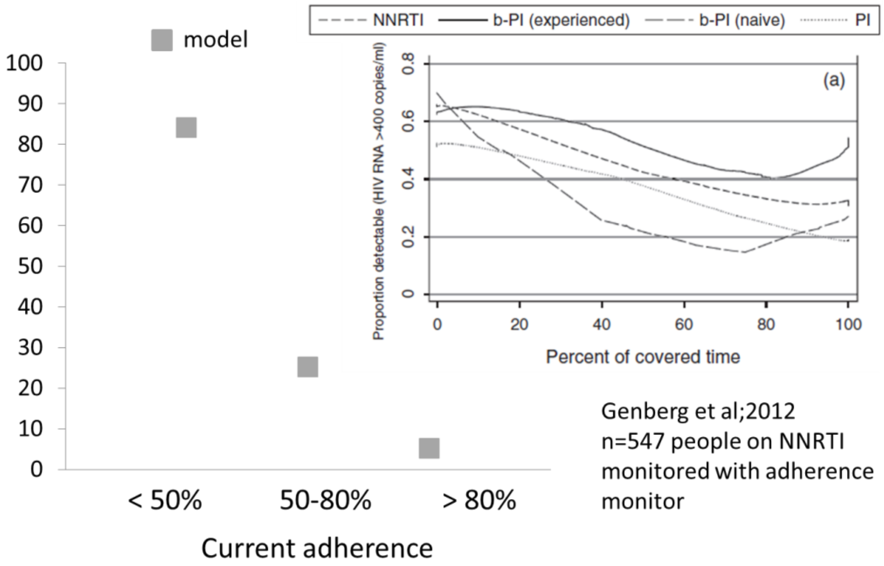

Regarding the risk of new drug resistant mutations arising, Tables S8a-S8c provide a number for "new mutation risk" that is multiplied by the viral load (mean of values at t-1 and t) to give a probability used when assessing whether a new mutation(s) has/have arisen. Values of the new mutations risk have been chosen in conjunction with the translation of presence of mutations into reduced drug activity to provide estimates of resistance accumulation consistent with those observed in clinical practice (Gallant 2004;Harrigan 2005;Johannessen 2009;Ledergerber 1999;Phillips 2001;Phillips 2005;Staszewski 1999a;Staszewski 1999b;van Leth 2004). Risk of new resistance mutations arising increases with decreasing number of active drugs, reflecting the known greater risk of resistance with regimens less able to suppress viral replication, most clearly seen in the fact that mono and dual therapy regimens are highly susceptible to resistance development (Havlir 1995;Kuritzkes 1996;Larder 1995). At low adherence levels, the risk of resistance development is generally low regardless of the number of active drugs, as drug selection pressure is low. However, for those on NNRTI regimens the new resistance mutation risk is assumed to be that for the effective adherence category of 50 – 80% (i.e. maximal) even if the effective adherence is below 50%, reflecting the fact that NNRTI resistance develops easily, even when drug exposure is very low (Bangsberg 2004;Bangsberg 2006b).

Viral load, CD4 count and risk of resistance between 3-6 months from (re-)starting ART

For the period 3-6 months from (re-)start of ART (Table S8b; to reduce the table content we do not provide the matrices of values for the resistance risk or CD4 count, only for the viral load – available in Cambiano et al 2014) we consider the adherence in both the current and previous 3 month period, since the likelihood of reaching viral suppression by 6 months will depend on adherence throughout the whole 6 month period from start of

ART, although the adherence in the current period is assumed to be the stronger factor. By 6 months after starting ART, those on 3 or more active drugs with consistently high adherence generally reach a relatively high level of viral suppression, regardless of pre-ART maximal viral load, so a person's viral load is no longer given by the change from baseline but the absolute level of viral load which it is likely they have reached. In these optimal conditions of high adherence and maximal active drugs we assume the viral load has a mean value of 0.5 log, again with variability between individuals. Since most viral load assays have a lower limit of quantification of 40 or 50 copies per mL, it is not actually known what the actual viral load level is, although highly sensitive assays suggest that a proportion of patients reach below 5 copies/mL (0.7 log copies/mL) (Doyle 2012). At lower numbers of active drugs and lower adherence, the viral load is still related to the maximal pre-ART viral load rather than being an absolute value, as the person's viral load has not become so low that the initial value loses relevance. The viral load response decreases with a lower number of active drugs, lower current adherence, and lower adherence in the previous 3 month period. Values for the viral load response between those known from studies (high level of suppression for 3 active drugs and maximal adherence, and only around 0.5 log viral suppression when adherence is < 0.5 even with three active drugs (Gross 2001;Wittkop 2011) are imputed assuming a monotonic relationship. CD4 count responses again mirror the viral load response, as has been extensively studied in patients with ongoing viraemia on ART (Ledergerber 2004). Risk of new resistance mutations again increases with decreasing number of active drugs, if current adherence is in the middle or highest group. The only situation in which risk of new mutations is extremely low is when the number of active drugs is 3 or close to 3 and the current adherence is in the high category.

Viral load, CD4 count and risk of resistance after 6 months of (re-)starting ART

Table S8c shows how the viral load, CD4 count and risk of resistance is determined for the situation where a person has been on ART for more than 6 months and the viral load is suppressed or partially suppressed (< 4 log copies/mL). These values are similar to those used for the period 3-6 months from start of ART except that there is assumed to dependence on the adherence in the current 3 month period only.

The situation where the viral load is above 4 log copies /mL, 10,000 copies/mL is treated the same as that in the period 3-6 months from start of ART (described above), with adherence in the current and previous period having some influence.

Table S8a. Viral load (mean change from viral load max), CD4 count change (mean change between t-1 and t), and new mutation risk in first 3 months. For 0 active drugs, these are the changes regardless of time from start of ART. For viral load this is the mean of a Normal distribution with standard deviation 0.2, from which the patient's value/change is sampled. For the CD4 count patients vary in their underlying propensity for CD4 rise on ART (given by sampling from lognormal($1,0.5^2$) and the CD4 count change given here is multiplied by this factor. For the new mutation risk, this is a number that is multiplied by the viral load (mean of values at t-1 and t). The resulting probability is used when assessing whether a new mutation or mutations have arisen.

	Effective adherence between t-1 & t	Number of active drugs												
		3	2.75	2.5	2.25	2.0	1.75	1.5	1.25	1	0.75	0.5	0.25	0
Viral load	≥ 80%	-3.0	-2.6	-2.2	-1.8	-1.5	-1.25	-0.9	-0.8	-0.7	-0.55	-0.4	-0.3	-0.3
(log change	≥ 50%, < 80%	-2.0	-1.6	-1.2	-1.1	-0.9	-0.8	-0.6	-0.5	-0.4	-0.25	-0.1	-0.05	-0.1
from vmax)	< 50%	-0.5	-0.4	-0.3	-0.25	-0.2	-0.15	-0.0	+0.05	+0.1	+0.1	+0.1	+0.1	-0.0
CD4 count	≥ 80%	+50	+45	+40	+35	+30	+25	+20	+17	+13	+10	+5	-2	-15
change	≥ 50%, < 80%	+30	+30	+23	+20	+15	+13	+10	+8	+5	+3	+0	-7	-17
(t-1 to t)	< 50%	+5	+4	+3	+2	+1	-1	-3	-6	-10	-11	-12	-13	-18
new mutation	≥ 80%	0.002	0.01	0.03	0.05	0.1	0.15	0.2	0.3	0.4	0.45	0.5	0.5	0.5
risk	≥ 50%, < 80%	0.15	0.15	0.2	0.25	0.3	0.3	0.3	0.35	0.4	0.45	0.5	0.5	0.5
(x log viral load)	< 50%*	0.15	0.15	0.2	0.25	0.3	0.3	0.3	0.35	0.4	0.45	0.5	0.5	0.5
	< 50%**	0.05	0.05	0.05	0.05	0.05	0.05	0.05	0.05	0.05	0.05	0.05	0.05	0.05

* for NNRTI containing regimen, ** for boosted PI containing regimen.

Table S8b. Summary of viral load (mean absolute value or mean change from viral load max) between 3-6 months, and after 6 months if viral load at t-1 > 4 logs. This is the mean of a Normal distribution with standard deviation 0.2, from which the patient's value/change is sampled.

Effective adherence between t-2 & t-1	Effective adherence between t-1 & t	Number of active drugs: 3	2.75	2.5	2.25	2.0	1.75	1.5	1.25	1	0.75	0.5	0.25
≥ 80%	≥ 80%	0.5	0.8	1.2	1.4	2.0	2.7	-1.7	-1.15	-0.9	-0.75	-0.6	-0.4
≥ 50%, < 80%	≥ 80%	1.2	1.2	1.2	1.4	-2.0	-1.6	-1.2	-1.05	-0.9	-0.7	-0.5	-0.35
< 50%	≥ 80%	1.2	1.2	1.2	1.4	-2.0	-1.6	-1.2	-1.0	-0.9	-0.7	-0.5	-0.2
≥ 80%	≥ 50%, < 80%	1.2	1.6	1.8	2.2	2.4	-2.4	-1.5	-0.9	-0.7	-0.55	-0.4	-0.3
≥ 50%, < 80%	≥ 50%, < 80%	2.5	2.5	2.5	2.5	-1.2	-1.1	-0.8	-0.65	-0.5	-0.35	-0.2	-0.05
< 50%	≥ 50%, < 80%	-2.0	-1.8	-1.5	-1.35	-1.2	-1.1	-0.8	-0.65	-0.5	-0.2	-0.2	-0.05
≥ 80%	< 50%	-0.5	-0.4	-0.3	-0.25	-0.2	-0.15	-0.10	-0.05	+0.0	+0.0	+0.0	+0.0
≥ 50%, < 80%	< 50%	-0.5	-0.4	-0.3	-0.25	-0.2	-0.15	-0.10	-0.05	+0.0	+0.0	+0.0	+0.0
< 50%	< 50%	-0.5	-0.4	-0.3	-0.25	-0.2	-0.15	-0.10	-0.05	+0.0	+0.0	+0.0	+0.0

Table S8c. Summary of viral load (mean change from viral load max), CD4 count change (mean change between t-1 and t), and new mutation risk after 6 months, where viral load at t-1 < 4 logs. For viral load this is the mean of a Normal distribution with standard deviation 0.2, from which the patient's value/change is sampled. For the CD4 count patients vary in their underlying propensity for CD4 rise on ART (given by sampling from lognormal(1,0.5^2) and the CD4 count change given here is multiplied by this factor. For the new mutation number, this is a number that is multiplied by the viral load (mean of values at t-1 and t). The resulting probability is used when assessing whether a new mutation or mutations have arisen.

	Effective adherence between t-1 & t	Number of active drugs											
		3	2.75	2.5	2.25	2.0	1.75	1.5	1.25	1	0.75	0.5	0.25
Viral load (absolute value or log change from vmax)	≥ 80%	0.5	0.9	1.2	1.6	-2.5	-2.0	-1.4	-1.15	-0.9	-0.75	-0.6	-0.3
	≥ 50%, < 80%	1.2	1.2	1.2	1.4	-1.2	-1.0	-0.7	-0.6	-0.5	-0.4	-0.3	-0.1
	< 50%	-0.5	-0.4	-0.3	-0.25	-0.2	-0.2	-0.1	-0.1	-0.1	-0.1	-0.1	-0.0
CD4 count change (t-1 to t)	≥ 80%	+30	+28	+25	+23	+21	+19	+3	-5	-9	-10.5	-12	-12
	≥ 50%, < 80%	+15	+13	+10	+8	-4.5	-7.5	-10	-12	-13	-14	-15	-15
	< 50%	-13	-14	-15	-15.5	-16	-16.5	-17	-17	-18	-17	-17	-17
new mutation risk (x log viral load)	≥ 80%	0.002	0.01	0.03	0.08	0.10	0.15	0.2	0.3	0.4	0.45	0.5	0.5
	≥ 50%, < 80%	0.15	0.18	0.2	0.25	0.3	0.3	0.3	0.35	0.4	0.45	0.5	0.5
	< 50%*	0.15	0.18	0.2	0.25	0.3	0.3	0.3	0.35	0.4	0.45	0.5	0.5
	< 50% **	0.05	0.05	0.05	0.05	0.05	0.05	0.05	0.05	0.05	0.05	0.05	0.05

* for NNRTI containing regimen, ** for boosted PI containing regimen.

Table S9. Example of model outputs of status of people who started ART, according to time since initiation.

Status	Number of years since start ART 1	3	5	10
On ART VL < 500	76%	65%	56%	39%
On ART VL > 500 no resistance	3%	3%	2%	1%
On ART VL > 500 with 1 or more resistance mutation	7%	8%	6%	5%
Off ART but under care	3%	3%	3%	2%
Off ART not under care	2%	4%	4%	4%
Dead AIDS	5%	10%	16%	27%
Dead non-AIDS	3%	8%	12%	21%

Table S10. Example of model-derived Kaplan-Meier estimates of percentage experiencing various outcomes by years from initiation of ART.

	Years from start of ART 1	3	5	10
Viral load failure*	9	17	25	38
Resistance mutation (with virologic failure)	8	14	18	25
CD4 count rise of > 100/mm^3	59	84	90	95
CD4 count rise of > 200/mm^3	17	63	77	88
Interruption	14	31	44	68
Loss to follow-up	4	10	15	28
Death	8	17	25	44

* considering viral load annually only, for consistency with countries where virologic failure is defined according to the WHO monitoring guidelines.

Note that these outputs will vary by model run because relevant parameters, such as those relating to ART adherence and ART interruption, are sampled.

Variable patient-specific tendency for CD4 count rise on ART

There is variability in the tendency for the CD4 count to rise on ART, for a given level of viral load suppression. For scenarios in Table S8 in which the CD4 count change is positive the CD4 count change is modified by this patient-specific factor (i.e. it is fixed for each patient), which is given by sampling for each patient from

Exp (N(0, (*sd_patient_cd4_rise_art*)^2)

sd_patient_cd4_rise_art = 0.2

To reflect the fact that the rate of CD4 count increase on ART tends to diminish with time, for those with patient-specific factor determining the CD4 count rise on ART > 1, this factor is modified by a factor 0.25 after 2 years of continuous treatment.

Accelerated rate of CD4 count loss if PI not present in regimen

The rate of change in CD4 count in people on failing regimens is largely based on data from the PLATO collaboration, for which patients were mainly on regimens containing a PI. If the regimen does not contain a PI the change in CD4 count per 3 months is modified (in the base model) by *poorer_cd4_rise_on_failing_nnrti* (= -6 /mm^3). This applies regardless of viral load level, so PIs are assumed to lead to a more beneficial CD4 count change than NNRTIs (Ledergerber 2004).

Variability in individual (underlying) CD4 counts for people on ART

Once the mean of the underlying CD4 count is obtained as described above for people on ART, to obtain the CD4 count, variability (*sd_cd4* = 1.2) is added on the square root scale. The estimate was based on unpublished analyses.

Viral load and CD4 count changes during ART interruption

Viral load returns to previous maximum viral load (vmax) in 3 months and adopts natural history changes thereafter.

CD4 rate of decline returns to natural history changes (i.e. those in ART naïve patients) after 9 months, unless the count remains > 200 above the CD4 nadir

Rate of CD4 count decline depends on current viral load. c(t) is the CD4 count at time t, cmin(t) is the CD4 count nadir measured by time t and cc(t-1) is the change in CD4 count from t-1 to t. v

if time off ART = 3 or if time off ART > 3 months and CD4 in previous period is > 300 above the minimum CD4 count to date

v(t) = vmax(t-1)

if v(t) $\geq$ 5	then cc(t-1) = Normal (-200,10^2)
if 4.5 <= v(t) < 5	then cc(t-1) = Normal (-160,10^2)
if v(t) < 4.5	then cc(t-1) = Normal (-120,10^2)

If this leads to c(t) < cmin(t) (CD4 nadir) then c(t) is set to cmin(t)
if time off ART = 6 months:-

if v(t) $\geq$ 5	then cc(t-1) = Normal (-100,10^2)
if 4.5 <= v(t) < 5	then cc(t-1) = Normal (-90,10^2)
if v(t) < 4.5	then cc(t-1) = Normal (-80,10^2)

if time off ART = 9 months:-

if v(t) $\geq$ 5	then cc(t-1) =	Normal (-80,10^2)
if 4.5 <= v(t) < 5	then cc(t-1) =	Normal (-70,10^2)
if v(t) < 4.5	then cc(t-1) =	Normal (-60,10^2)

This is broadly based on evidence from a number of analyses of the effects of ART interruption (e.g. d'Arminio Monforte 2005, Li X 2005, Mocroft 2001, Wit 2005)

Incidence of new current toxicity and continuation of existing toxicity

Toxicities including gastrointestinal symptoms, rash, hepatoxicity, CNS toxicity, lipodystrophy, hypersensitivity reaction, peripheral neuropathy and nephrolithiasis can occur with certain probability on certain specific drugs. These probabilities are based broadly on evidence from trials and cohort studies, although there are no common definitions for some conditions which complicates this. Details of toxicities of each drug are provided in Appendix II of Cambiano V PhD thesis (2014) and also Nakagawa F. PhD thesis (2015).

Table S11. Risk of development of specific drug toxicities.

**Toxicity**	**Drug**	**Risk of development per 3 months**	**Probability of continuation if pre-existing**
Nausea	atazanavir	1% (5-fold higher in 1st year)	50%
	Zidovudine, ddI, lopinavir	3% (5-fold higher in 1st year)	50%
	ddI	5% (2.5-fold higher in 1st year)	50%
	lopinavir	2% (2.5-fold higher in 1st year)	50%
	atazanavir	1% (2.5-fold higher in 1st year)	50%
Rash	efavirenz	3% (in first 6 months on efavirenz)	
	nevirapine	10% (in first 6 months on nevirapine)	
CNS toxicity	efavirenz	10% (if been on efavirenz <1 year)	80% if been on efavirenz <1 year. 90% if been on efavirenz ≥1 year
Lipodystrophy	d4T	5%	100%
	Zidovudine	1.5%	100%
Peripheral neuropathy	d4T	2% (1.5-fold higher in 1st year)	100% (if remain on d4T)
	ddI	1% (1.5-fold higher in 1st year)	100% (if remain on ddI)
Acute hepatitis	nevirapine	2% (one off risk in 1st and 2nd 3 month periods)	

Anaemia	zidovudine	3% (1.5-fold higher in 1st year)	20%
Headache	ZDV	10% (1.5-fold higher in 1st year)	40%
Pancreatitis	D4T, DDI	0.5% (1.5-fold higher in 1st year)	100%
Lactic acidosis	d4T, ddI	1%	
	Zidovudine, tenofovir	0.01%	
Renal dysfunction	tenofovir	0.35%	

Switching of drugs due to toxicity

If toxicity is present then individual drugs may be switched due to toxicity (nevirapine for efavirenz, zidovudine for tenofovir). ddI is only used if neither zidovudine and tenofovir are available due to toxicity.

2.7. Emergence of specific resistance mutations and their effect on drug activity

Accumulation of resistance mutations
newmut is a probability used to indicate the level of risk of new mutations arising in a given 3 month period (see section Effect of ART on viral load, CD4 count, resistance development and drug toxicity**)**. If this chance comes up in a given 3 month period (determined by sampling from the binomial distribution) then the following criteria operate.

Table S12. Risk of acquiring new resistance mutations.

**Resistance mutation**	**Probability of arising**	**Conditions**
M184	50%	if (on 3TC)
# TAMS increases by 1	20%	if (on ZDV or d4T) and (not on 3TC nor FTC)
	12%	if (on ZDV or d4T) and (on 3TC or FTC)
# TAMS increases by 2	1%	if (on ZDV or d4T) and (not on 3TC nor FTC)
	1%	if (on ZDV or d4T) and (on 3TC or FTC)

K65	2%	if (on tenofovir or ddI) and (on zidovudine or d4T)
	10%	If (on tenofovir or ddI) and (not on zidovudine nor d4T)
L74	1%	if (on ddI)
Q151	2%	if (on ddI or d4T or zidovudine)
K103	20%	If on nevirapine
	60%	If on efavirenz
Y181	40%	If on nevirapine
	10%	If on efavirenz
G190	20%	If on nevirapine
	10%	If on efavirenz
V32	4%	if on lopinavir
I47	4%	If on lopinavir
I50L	3%	If on atazanavir
L76	4%	If on lopinavir
I84	3%	If on atazanavir
N88	3%	If on atazanavir

These values are chosen, in conjunction with values of *newmut{t}*, to provide estimates of accumulation of specific classes of mutation consistent with those observed in clinical practice (UK Drug Resistance Database 2005, Harrigan 2005, Sigaloff 2012). They reflect a greater propensity for some mutations to arise than others. This probably relates to the ability of the virus to replicate without the mutations (e.g. probably very low in the presence of 3TC for virus without M184V) as well as the replicative capacity of virus with the mutations. Over time as more data accumulate it may be possible improve these estimates of rates of accumulation of specific mutations. Further details can be found in Cambiano V PhD thesis (2014) and also Nakagawa F. PhD thesis (2015).

New resistance to NNRTI arising as a result of ART interruption

It is assumed that due to the long half-life of NNRTIs nevirapine and efavirenz, stopping of a regimen containing one of these drugs is associated with a probability (= 0.05) of an NNRTI resistance mutation arising (see, for example, Fox et al, 2008).

Loss of acquired mutations from majority virus

It is assumed that mutations tend to be lost from majority virus with a certain probability from 3 months after stopping to take a drug that selects for that mutation. The probability of losing mutations per 3 months (from 3 months after stopping) is as follows (Devereux 1999, Devereux 2001, Deeks 2003, Birk 2001, Walter 2002, Hance 2001, Tarwater PM 2003)

Table S13. Probability of loss of acquired mutations from majority virus per 3 months after stopping drugs selecting for mutation. This is based on evidence from studies in people interrupting ART (Devereux HL, et al, Birk M, et al, Deeks SG, et al, Walter H, et al., Hance AJ, et al, Tarwater PM, et al).

M184V	0.8
L74V	0.6
Q151M	0.6
K65R	0.6
TAMS (lose all)	0.4
NNRTI mutations	0.05
Protease mutations	0.2

Mutations are regained in majority virus if a drug selecting for the mutation is again started.

Determination of level of resistance to each drug

Table S14 shows the level of resistance to each drug according to presence of specific resistance mutations. These rules approximately follow the interpretation systems for conversion of mutations present on genotypic resistance test into a predicted level of drug activity (or, equivalently, of resistance; http://www.rega.kuleuven.be, http://hivdb.stanford.edu, http://www.hivfrenchresistance.org/

Table S14. Level of resistance to each drug according to presence of specific resistance mutations.

Resistance mutation	Drug	Level of resistance (1=full resistance)	Condition
M184	3TC or FTC	0.75	
1-2 TAMS	zidovudine or d4T	0.5	No 3TC or FTC in regimen
	zidovudine or d4T	0.25	3TC or FTC in regimen and ever had M184V
	zidovudine or d4T	0.5	3TC or FTC in regimen and never had M184V
2-3 TAMS	tenofovir	0.5	No 3TC or FTC in regimen
	tenofovir	0.25	3TC or FTC in regimen and ever had M184V
	tenofovir	0.5	3TC or FTC in regimen and never had M184V
3-4 TAMS	zidovudine or d4T	0.75	No 3TC or FTC in regimen

	zidovudine or d4T	0.5	3TC or FTC in regimen and ever had M184V
	zidovudine or d4T	0.75	3TC or FTC in regimen and never had M184V
3 or more TAMS	ddI	0.5	
4 or more TAMS	tenofovir	0.75	No 3TC or FTC in regimen
	tenofovir	0.5	3TC or FTC in regimen and ever had M184V
	tenofovir	0.75	3TC or FTC in regimen and never had M184V
5 or more TAMS	zidovudine or d4T	1.0	No 3TC or FTC in regimen
	zidovudine or d4T	0.75	3TC or FTC in regimen and ever had M184V
	zidovudine or d4T	0.75	3TC or FTC in regimen and never had M184V
Q151	zidovudine or d4T ddI	0.75	
K65	d4T	0.5	
	tenofovir or ddI	0.75	
	ddI	0.75	
K103	nevirapine or efavirenz	1.0	
Y181	nevirapine	1.0	
	efavirenz	0.75	
G190	nevirapine	1.0	
	efavirenz	0.75	
I47	lopinavir	0.75	
I50L	atazanavir	1.0	
	atazanavir	1.0	
N88	atazanavir	1.0	
1 of (G48, I84)	atazanavir	0.5	
1 of (G48, I84)	atazanavir	1.0	Ever had at least 2 of (V32, M46, I54, V82, L90)
Both of (G48, I84)	atazanavir	1.0	
1 or 2 or 3 of (V32, M46, I54, V82, L90)	atazanavir	0.5	
At least 4 of (V32, M46, I54, V82, L90)	atazanavir	1.0	
1 of (V32, L76, V82)	lopinavir	0.25	Never had I47
2 of (V32, L76, V82)	lopinavir	0.5	Never had I47
3 of (V32, L76, V82)	lopinavir	0.75	Never had I47
All of (V32, I47, L76, V82)	lopinavir	1.0	
4 of (M46, V82, I84, L90)	Lopinavir	Max(level of resistance as above in this table, 0.5)	
2 or 3 of (M46, V82, I84, L90)	lopinavir	Max(level of resistance as above in this table, 0.25)	

Calculation of activity level of each drug

This is given by 1-level of resistance. For ritonavir boosted PIs it is given by 2 – (2 x level of resistance); i.e. assumed higher potency due to ability to induce sustained viral suppression alone. Activity levels of each drug in the regimen are summed to give the total number of active drugs.

2.8. Risk of clinical disease and death in HIV infected people

Occurrence of WHO 4 diseases

The rate of WHO 4 diseases according to CD4 count per 3 months is given below.

Table S15. Rate of WHO stage 4 disease according to CD4 count and viral load.

If cd4 > 650	rate=0.002		
if 500 ≤ cd4 < 650	rate=0.010	if 450 ≤ cd4 < 500	rate=0.013
if 400 ≤ cd4 < 450	rate=0.016	if 375 ≤ cd4 < 400	rate=0.020
if 350 ≤ cd4 < 375	rate=0.022	if 325 ≤ cd4 < 350	rate=0.025
if 300 ≤ cd4 < 325	rate=0.030	if 275 ≤ cd4 < 300	rate=0.037
if 250 ≤ cd4 < 275	rate=0.045	if 225 ≤ cd4 < 250	rate=0.055
if 200 ≤ cd4 < 225	rate=0.065	if 175 ≤ cd4 < 200	rate=0.080
if 150 ≤ cd4 < 175	rate=0.10	if 125 ≤ cd4 < 150	rate=0.13
if 100 ≤ cd4 < 125	rate=0.17	if 90 ≤ cd4 < 100	rate=0.20
if 80 ≤ cd4 < 90	rate=0.23	if 70 ≤ cd4 < 80	rate=0.28
if 60 ≤ cd4 < 70	rate=0.32	if 50 ≤ cd4 < 60	rate=0.40
if 40 ≤ cd4 < 50	rate=0.50	if 30 ≤ cd4 < 40	rate=0.80
if 20 ≤ cd4 < 30	rate=1.10	if 10 ≤ cd4 < 20	rate=1.80
if 0 ≤ cd4 < 10	rate=2.50		

Independent effect of viral load

if v < 3	rate = rate x 0.2
if 3 <= v < 4	rate = rate x 0.3
if 4 <= v < 4.5	rate = rate x 0.6
if 4.5 <= v < 5	rate = rate x 0.9
if 5 <= v < 5.5	rate = rate x 1.2
if 5.5 <= v	rate = rate x 1.6

This is informed by Phillips AIDS 2004.

Independent effect of age

rate = rate x $(\text{age} / 38)^{1.2}$

Independent effect of PJP prophylaxis

If patient on PJP prophylaxis then this rate is multiplied by 0.8.

If CD4 count is measured and current value < 350 /mm3 then patient assumed to have 80% chance of starting PJP prophylaxis after 1996

If patient has current WHO stage 3 or 4 condition they are assumed to have an 80% chance of starting PJP prophylaxis
If CD4 count is measured then PJP prophylaxis assumed to stop if current value > 350/mm3.

If the patient has been continuously on ART for 2 years with no WHO 3 or 4 condition in previous 6 months then it is assumed that PJP prophylaxis is stopped.

Independent effect of being on ART

For patients on a single drug regimen this risk is multiplied by 0.9, for patients on a two drug regimen it is multiplied by 0.85 and for patients on a 3 drug regimen it is multiplied by 0.6, to reflect that being on ART has a positive effect on risk of AIDS and death independent of latest CD4 count and viral load.

Occurrence of WHO 3 diseases
As for WHO 4 except risk is fold_incr_who3 (= 5) higher.

Risk of HIV-related death

As for WHO 4 except risk *fold_decr_hivdeath* - fold lower (= 0.25).

CD4-, viral load- age-specific death rate raised *incr_death_rate_tb*-fold (= 10) if current TB and *incr_death_rate_adc*-fold (= 10) if current WHO 4 disease. We assume 15% of HIV-related deaths (i.e. not including deaths that arise due to background mortality rates) are classified as non-HIV-related.

2.9. Parameter distributions sampled for each model run (each model run creates one setting scenario)

For this project we based the demographics of the population studied and HIV epidemic features around those for Malawi and then sampled widely from parameter distributions in order to generate diverse setting scenarios in respect of many epidemic aspects, such as sexual behaviour, HIV prevalence, ART uptake and HIV incidence. The comparison of these setting scenarios with observed data is shown in Table 1 of the main paper. The parameter distributions are described in Table 16.

Table S16. Parameter distributions sampled for each model run (base case).

Parameter name	Description	Distribution (value; % with value)	Motivation for distribution
Parameters relating to sexual behaviour See section 2.3.			
swn	Value of multiplicative factor determining numbers of partners for those in highest new partner group (i.e. female sex workers)	4 13.22<br>8 19.18<br>12 21.34<br>16 23.52<br>20 22.74	This parameter helps to determine the extent to which the epidemic is driven by transactional sex, which is likely to vary in specific setting scenarios. The proportion of women wo are sex workers varies from < 0.5% to over 4% (Vandepitte et al, 2006)
highsa	Value of and fold change in multiplicative factor determining numbers of partners for those in second highest new partner group	4 20.62<br>5 21.34<br>6 18.54<br>7 19.06<br>8 20.44	Range of values that was found, in certain (randomly selected) combination with other sexual behaviour parameter values to re-produce epidemics within the observed prevalence range. Note also that sexual behaviour tends to be under-reported, particularly in women, and higher levels of behaviour have to be assumed both to be consistent with levels of risk behaviour reported in men, and to generate an epidemic of the proportions observed (e.g. Gregson 20022, Johnson 2009).
sex_beh_trans_matrix	Matrix determining rate of transition between four levels of sexual behaviour. There are two versions (see section 2.3)	A 8.46<br>B 91.54	Range of values that was found, in combination with other sexual behaviour parameter values to re-produce epidemics within the observed prevalence range. We considered that version B

			is more likely to be closer to reality mainly because it leads to a lower proportion of men and women with high numbers of condomless partners, more in keeping with data from the DHS (Malawi Demographic and Health Survey), albeit that the DHS data are not restricted to condomless sex.
p_rred_p	Indicates the proportion of the population in whom the sexual risk behaviour is very low	0.1 17.16<br>0.2 19.34<br>0.3 19.40<br>0.4 20.40<br>0.5 23.70	In order to include a person-level effect on sexual behaviour this and the parameter below allow the population to be divided into three according to the lifelong tendency to have condomless sex. This range of values that was found, in some combination with other sexual behaviour parameter values, to be able to re-produce epidemics within the observed prevalence range.
p_hsb_p	Indicates the proportion of the population in whom the sexual risk behaviour has a tendency to be higher than average	0.02 4.04<br>0.05 17.96<br>0.1 25.72<br>0.15 26.62<br>0.2 25.66	As above
newp_factor	Overall average level of sexual risk behaviour. The correlation with the above parameters induced by the sampling of this parameter is to provide a focus on parameter space most likely to give low values of the overall fit. For example, if the sampling of *swn* and *highsa* give values at the high end of the distribution and sampling of *p_rred_p* produces a value at the low end then the model simulation run will produce an epidemic which is too large, unless there is some compensation when selecting the value of this parameter.	3 x (4/highsa)x(7/swn) x (p_rred_p/0.2) x (0.1/p_hsb_p) x exp(Normal(0, 0.5^2))	See description of parameter
conc_ep	Parameter indicating the degree to which those with a long term condomless sex partner have a	Lognormal(0,0.6)	This is likely to vary across setting scenarios and we wished to consider across the range. Again,

	lower of higher probability of short term condomless sex partners than those without a long term condomless sex partner.			this distribution of values was found, in certain (randomly selected) combination with other sexual behaviour parameter values to re-produce epidemics within the observed prevalence range.
ych_risk_beh_newp	Degree of reduction in condomless sex with short term partners per year from 1995 – 2000	0.12<br>0.14<br>0.16<br>0.18<br>0.2	25.00<br>24.58<br>22.48<br>18.18<br>9.76	In order to explain the decrease in incidence and prevalence of HIV in southern Africa in the late 1990s it is necessary to assume there was a reduction in condomless sex, which is supported by data in Zimbabwe, for example (Gregson 2010, Halperin 2011).
ych_risk_beh_ep	Degree of reduction in condomless sex per year with long term partners from 1995-2000	0<br>0.02<br>0.04<br>0.06<br>0.08	20.52<br>21.08<br>20.24<br>19.88<br>18.28	As above
ch_risk_diag_newp	Degree of reduction (fold change) in condomless sex with short term partners in a person diagnosed with HIV	0.7<br>0.8<br>0.83<br>0.9<br>1	25.14<br>25.20<br>5.50<br>24.78<br>19.38	Informed by Fonner et al 2012
ch_risk_diag	Degree of reduction in condomless sex with long term partner in a person diagnosed with HIV	0.7<br>0.8<br>0.9<br>1	30.26<br>25.50<br>24.70<br>19.54	Informed by Fonner et al 2012
ych2_risk_beh_newp	Degree of change in condomless sex with short term partners per year from 2010 – 2015	-0.04<br>-0.02<br>0<br>0.02<br>0.04	6.56<br>6.62<br>73.38<br>6.78<br>6.66	It is uncertain whether there have been recent changes in condomless sex, hence a neutral distribution was used.

**Parameters relating to transmission** See section 2.3.				
fold_change_w	The fold difference in female to males transmission rate compared with male to female, for a given viral load.	1<br>1.25<br>1.5	8.62<br>8.64<br>82.74	Informed by the higher incidence and prevalence in women in younger age groups and some direct evidence.
res_trans_factor	parameter determining the probability that if NNRTI resistance mutation present in source partner that this is not present/detectable in virus new host	0.67<br>0.8<br>1<br>1.5<br>2<br>3<br>4	4.58<br>4.86<br>19.64<br>20.06<br>26.32<br>19.70<br>4.84	Informed by the values needed to lead to the range of transmitted NNRTI resistance observed (Alfonso et al 2012, Rowley et al 2016, National Institute for Communicable Diseases 2016)
**Parameters relating to HIV testing** See section 2.5.				
an_lin_incr_test	Parameter determining the rate of increase in HIV testing (any testing outside ANC)	0.0005<br>0.002<br>0.005<br>0.01	24.72<br>24.60<br>26.20<br>24.48	Range and pattern required to re-produce the observed range in proportion of HIV positive people diagnosed (see Table 1 of main paper).
*date_test_rate_plateau_*	Year in which the rate of HIV testing plateaus.	2011.5<br>2013.5<br>2015.5	34.16<br>32.58<br>33.26	Countries have increased testing rates markedly and these have plateaued at different levels in different settings (e.g Government of Malawi Ministry of Health Quarterly Reports).
rate_testanc_inc	Rate of increase in testing in ANC clinics	0.005<br>0.01<br>0.015<br>0.02	24.72<br>25.12<br>25.34<br>24.82	Government of Malawi Ministry of Health Quarterly Reports. Again distribution is intended to reflect variation across setting scenarios.
*incr_test_rate_sympt_*	The rate of increase over time in the probability of a person with a WHO stage 3 or 4 disease is tested for HIV.	1.00<br>1.05<br>1.10<br>1.15<br>1.20	19.44<br>20.00<br>19.82<br>19.24<br>21.50	Little direct data on this parameter and wide range taken to reflect uncertainty and variation across settings.

**Parameters relating to pre-ART care and progression of HIV**			
fx	Multiplicative factor to alter the average rate of CD4 count decline in natural HIV progression (which thus alters the incubation period distribution).	0.7 21.50<br>0.8 19.92<br>0.9 19.90<br>1 19.62<br>1.1 19.06	Derived based on consideration of evidence from natural history studies (Pantazis 2005, Sabin JAIDS 2000, Hubert J-B 2000, O'Brien 1998, Henrard 1995, Lyles 2000, Touloumi 2004, Mellors 1997, Koot 1993). See section 2.4.
prob_loss_at_diag	Probability that a person is immediately lost after initial HIV diagnosis.	0.1 43.20<br>0.25 33.28<br>0.4 11.34<br>0.55 12.18	Rosen at al (2011)
rate_lost	For people under care yet to start ART or previously have taken ART, the rate of being lost to care per 3 mths.	0.05 20.64<br>0.1 19.68<br>0.15 20.22<br>0.3 19.86<br>0.5 19.60	Uncertain and will vary by setting. Distribution chosen to reflect this. This is one of the parameters influencing the proportion of diagnosed people on ART.
rate_return	Probability of return to care for a person who has been diagnosed with HIV (and may have started ART) but is now lost and not on ART, without current WHO stage 3 or 4 disease, per 3 months.	0.01 19.66<br>0.1 19.78<br>0.15 20.18<br>0.2 20.00<br>5 20.38	As above
prob_return_adc	Probability of return to care for a person who has been diagnosed with HIV (and may have started ART) but is now lost and not on ART and has a WHO stage 4 condition. This is a probability that operates just for the 3 month period that the events occurs.	0.2 25.10<br>0.4 24.64<br>0.6 23.74<br>0.8 26.52	As above

rate_loss_persistence	Rate of loss from majority virus of transmitted resistance mutations (per 3 months)	0<br>0.005<br>0.01<br>0.015<br>0.02<br>0.04	8.28<br>10.36<br>9.26<br>40.06<br>30.62<br>1.42	Jain et al JID 2011; Castro et al JID 2013; Yang et al PLOS Pathogens 2015.
**Parameters relating to people on ART** (see section 2.6)				
adh_pattern	Population adherence profile; described in terms of the proportion having a given average adherence and period-to-period variability in adherence.	B<br>C<br>D<br>E<br>F<br>G<br>H<br>I	35.70<br>23.06<br>2.94<br>2.10<br>21.90<br>8.92<br>3.58<br>1.80	Reflection of wide range of adherence profiles in different settings, informed by differences in proportions of people on ART with viral load suppression.
pr_art_init	Probability of ART initiation per 3 months in a person in care who is eligible according to current criteria.	0.1<br>0.2<br>0.3<br>0.4	23.86<br>25.60<br>24.46<br>26.08	These parameters contribute to determine the proportion of HIV diagnosed people who are on ART. The distributions are chosen such that combinations of these parameters lead to observed proportions of HIV diagnosed people on ART (e.g. Justman et al, Huerga et al, Maman et al).
prob_lost_art	For a person who interrupts / stops ART the probability that they are simultaneously lost from care.	0.5<br>0.6<br>0.7<br>0.8<br>0.9	19.98<br>20.22<br>20.38<br>19.14<br>20.28	Kranzer at al 2011. McMahon et al 2016. See also Fig S14 and rationale described in Section 2.6.
rate_restart	Rate of restart of ART for people who previously have been on ART and have returned to care, per 3 months.	0.2<br>0.4<br>0.6<br>0.8	24.84<br>25.06<br>25.48<br>24.62	Kranzer at al 2010. Assumed to be high, given the person has returned to care. Most people who are regularly seen in clinics who have previously started ART are on ART.

rate_int_choice	Rate of interruption / stopping of ART per 3 months. Also influenced by current drug toxicity and underlying tendency to adhere.	0.005 20.44<br>0.01 20.74<br>0.015 36.90<br>0.03 11.10<br>0.05 10.82	Kranzer at al 2011. McMahon et al 2016. See Fig S14 and rationale described in Section 2.6.
incr_rate_int_low_adh	Parameter indicating the extent to which people with a long term average adherence in the lowest group have a multiplicatively increased risk of ART interruption.	1 50.52<br>2 49.48	Agbaji OO et al 2015.
pr_switch_line	Probability of switch to second line per 3 months in a person who has fulfilled the failure criteria for first line failure.	0.005 25.96<br>0.02 24.52<br>0.05 24.90<br>0.2 24.62	Fox 2012; Johnston 2012. In several settings, including Zimbabwe, the proportion of people who have started second line ART is consistent with a value for *pr_switch*_line of below 0.1 (e.g. Lesotho, Malawi) (Government of Malawi Ministry of Health Quarterly Reports).
clinic_not_aw_int_frac	If a person interrupts ART, the probability that this is not disclosed to the clinic and they are classified as being on ART	0.1 19.72<br>0.3 20.16<br>0.5 19.98<br>0.7 20.24<br>0.9 19.90	Uncertain and will vary by setting, hence a broad distribution.
poorer_cd4_failing_nnrti	Extent to which CD4 decline is greater in those virologically failing an NNRTI regimen.	< 10 / mm^3 / 3 months 6.64<br>8 – 10 13.26<br>6 – 8 23.08<br>4 – 6 26.56<br>2 - 4 18.64<br>0 – 2 8.60<br>< 0 3.22	PIs are assumed to lead to a more beneficial CD4 count change than NNRTIs (Ledergerber 2004).
*fold_change_mut_risk_*	Fold difference in rate of accumulation of mutations (for all drugs) compared with base case.	1 95.38<br>2 4.62	To consider that the rate of resistance mutation acquisition is two fold higher than that assumed, but this is thought to be unlikely given the data to inform this parameter (hence the low

			proportion with value 2). See Fig S11, S12 Cozzi Lepri et al 2010.
*rate_res_ten_*	Parameter reflecting the rate of acquisition of tenofovir resistance. The value of 0.1 was derived based on European cohort data and the value of 0.3 reflects the potentially higher value for subtype C in southern Africa.	0.1 9.88<br>0.3 90.12	The TenoRes Study Group. Global epidemiology of drug resistance after failure of WHO recommended first-line regimens for adult HIV-1 infection: a multicentre retrospective cohort study. Lancet Infect Dis 2016
*dol_extra_failure_*	raised risk of viral load rebound beyond that for efavirenz (not necessarily with resistance, e.g. due to lowering of drug levels due to co-use with rifampicin)	0 90.60<br>1 9.40	See explanation in main text under “Assumptions on properties of dolutegravir compared with efavirenz**”.**<br><br>Walmsley 2013, 2015, Clotet 2014, van Lunzen 2012, Stellbrink 2013, Sax 2017, Rutherford (2016), Patel 2014, de Boer 2016, Hoffmann 2017, Menard 2017, Taha 2015, Libre 2015, Marcellin 2016, Nicolè 2016, Katlama 2016, Wainberg 2015, Blanco 2017, Wijting 2017, Maggiolo 2017, Gubavu 2016, Borghetti 2016.
*dol_higher_potency_*	Potency relative to efavirenz	1 fold 11.20<br>1.5 fold 78.50<br>2 fold 10.30	
*rel_dol_tox_*	Relative rate of neurologic toxicity (sleep disturbance for dolutegravir and dizziness and vivid dreams for efavirenz)	1.5 fold higher for efavirenz 90.40<br>Equal to efavirenz 9.60	

2.10. Unit Costs and disability weights for DALYs

Table S17.

Item	Unit Cost	Source / explanation
Drug costs per year (including supply chain):<br><br>First-line: tenofovir/3TC/efavirenz<br><br>Tenofovir/3TC/dolutegravir<br><br>Second-line: zidovudine/3TC/atazanavir	 <br>$120 ($100 without supply chain costs<br><br>$127 ($106 without supply chain costs)<br><br>$343 ($286 without supply chain costs)	Untangling the web of antiretroviral price reductions. 18th Edition – July 2016. www.msfaccess.org.<br><br>http://www.unaids.org/en/resources/presscentre/pressreleaseandstatementarchive/2015/november/20151130_PR_CHAI_UNITAID
Cost of treatment of a WHO stage 4 condition over 3 months (cost is incurred for 3 months)<br><br>Cost of treatment of a WHO stage 3 condition over 3 months (cost is incurred for 3 months)<br><br>Cost of treatment of TB per 3 months (cost is incurred for 6 months)<br><br>Cotrimoxazole annual cost	$200<br><br>$20<br><br>$50<br><br>$5	Specific data not available on average unit costs of treating WHO stage 3 and 4 conditions and per clinic visit costs - costs used are informed by evidence synthesis from studies that cost according to current CD4 count of those in pre-ART care, cost of ART initiation, which also include costs of CD4 tests (Eaton J et al. Health benefits, costs, and cost-effectiveness of earlier eligibility for adult antiretroviral therapy and expanded treatment coverage: a combined analysis of 12 mathematical models. Lancet Global Health 2014: E23-E34)
CD4 count measurement	$10	Hyle, E. P., Jani, I. V, Lehe, J., Su, A. E., Wood, R., Quevedo, J., … Walensky, R. P. (2014). The Clinical and Economic Impact of Point-of-Care CD4 Testing in Mozambique and Other Resource-Limited Settings: A Cost-Effectiveness Analysis. PLoS Med, 11(9), e1001725. doi:10.1371/journal.pmed.1001725. Keebler D, Revill P, et al. How Should HIV Programmes Monitor Adults on ART? A Combined Analysis of Three Mathematical Models. Lancet Global Health 2014. E35-E43.

Viral load measurement:	$22	Human resource costs $3, sample collection consumables $2, relaying of results $2 (this costing information was provided by Medecin Sans Frontiers (MSF) (K Bonner), update February 2014), running the test (including equipment and other costs such as consumables, maintenance and shipping) $15 (http://www.theglobalfund.org/en/mediacenter/newsreleases/2015-06-10_New_Approach_on_HIV_Viral_Load_Testing/ http://www.theglobalfund.org/en/procurement/viral-load-early-infant-diagnostics/)
Genotypic resistance test cost	$100	Consensus from World Health Organisation HIV Resistance Network (HIVResNet).
Non-ART programme costs per year, $40 per year if on tiered care due to viral load < 1000	$80	Tagar E, Sundaram M, Condliffe K, Matatiyo B, Chimbwandira F, et al. (2014) Multi-Country Analysis of Treatment Costs for HIV/AIDS (MATCH): Facility-Level ART Unit Cost Analysis in Ethiopia, Malawi, Rwanda, South Africa and Zambia. PLoS ONE 9(11): e108304. doi:10.1371/journal.pone.0108304; Bill and Melinda Gates Foundation tiered care meeting report (the per client cost of running the Khayelitsha adherence clubs was $58 per client per year compared to standard clinic care of $108 per client per year. At the Infectious Disease Institute in Kampala, the annual costs per client for physician, nurse, and pharmacy only visits were $60, $45, and $19, respectively); Siapka M, Remme M, Dayo Obure C, Maier C, Dehne KL, Vassall A. Is there scope for cost savings and efficiency gains in HIV services? A systematic review of the evidence from low- and middle-income countries. Bull World Health Organ 2014;92:499–511AD | doi: http://dx.doi.org/10.2471/BLT.13.127639; Menzies NA, Berruti AA, Blandford JM (2012) The Determinants of HIV Treatment Costs in Resource Limited Settings. PLoS ONE 7(11): e48726. doi:10.1371/journal.pone.0048726
Cost of the targeted adherence counselling intervention triggered by a viral load > 1000 copies/mL	$10	Assumption
HIV test (including personnel costs)	$9	Eaton J et al. Health benefits, costs, and cost-effectiveness of earlier eligibility for adult antiretroviral therapy and expanded treatment coverage: a combined analysis of 12 mathematical models. Lancet Global Health 2014: E23-E34

Table S18. Disability weights (informed by Salomon et al*)

Values are 1 except for the following:

**Condition in current 3 month period**	**Disability weight for current 3 month period**	**Source**
Any drug toxicity in current 3-month period	0.95	Salomon et al 2010
Any WHO stage 3 condition (except TB) in current 3-month period	0.78	Salomon et al 2010
TB in current 3-month period	0.60	Salomon et al 2010
Any WHO stage 4 condition in current 3-month period	0.46	Salomon et al 2010

* Salomon JA, Vos T, Hogan DR, et al. Common values in assessing health outcomes from disease and injury: disability weights measurement study for the Global Burden of Disease Study 2010. Lancet 2012; 380: 2129–43.

References

Afonso JM, Bello G, Guimarães ML, Sojka M, Morgado MG. HIV-1 genetic diversity and transmitted drug resistance mutations among patients from the North, Central and South regions of Angola. PLoS ONE. 2012;7(8):e42996.

Agbaji OO, Abah IO, Falang KD, et al. Treatment Discontinuation in Adult HIV-Infected Patients on First-Line Antiretroviral Therapy in Nigeria. Curr HIV Research Volume: 13   Issue: 3   Pages: 184-192 (2015)

Arnsten, J. H. et al. Antiretroviral therapy adherence and viral suppression in HIV-infected drug users: comparison of self-report and electronic monitoring. Clinical infectious diseases 33, 1417-1423 (2001).

Bangsberg, D. R. et al. Adherence–resistance relationships for protease and non-nucleoside reverse transcriptase inhibitors explained by virological fitness. Aids 20, 223-231 (2006).

Bangsberg, D. R. Less than 95% adherence to nonnucleoside reverse-transcriptase inhibitor therapy can lead to viral suppression. Clinical Infectious Diseases 43, 939-941 (2006).

Bangsberg, D. R., Moss, A. R. & Deeks, S. G. Paradoxes of adherence and drug resistance to HIV antiretroviral therapy. Journal of Antimicrobial Chemotherapy 53, 696-699 (2004).

Bärnighausen, T. et al. Interventions to increase antiretroviral adherence in sub-Saharan Africa: a systematic review of evaluation studies. The Lancet infectious diseases 11, 942-951 (2011).

Bassett, I. V. et al. Routine voluntary HIV testing in Durban, South Africa: the experience from an outpatient department. Journal of acquired immune deficiency syndromes (1999) 46, 181 (2007)

Bellan SE, Dushoff J, Galvani AP, Meyers LA (2015) Reassessment of HIV-1 Acute Phase Infectivity: Accounting for Heterogeneity and Study Design with Simulated Cohorts. PLoS Med 12(3): e1001801. doi:10.1371/journal.pmed.1001801

Birk, M., Svedhem, V. & Sönnerborg, A. Kinetics of HIV-1 RNA and resistance-associated mutations after cessation of antiretroviral combination therapy. Aids 15, 1359-1368 (2001).

Blanco J et al. Pathways of resistance in subjects failing dolutegravir monotherapy. CROI 2017, 13-16 February 2017, Seattle. Oral abstract 42. http://www.croiconference.org/sessions/pathways-resistance-subjects-failing-dolutegravir-monotherapy (abstract)
http://www.croiwebcasts.org/console/player/33379 (webcast)

Bonner, K., Mezochow, A., Roberts, T., Ford, N. & Cohn, J. Viral load monitoring as a tool to reinforce adherence: a systematic review. JAIDS Journal of Acquired Immune Deficiency Syndromes 64, 74-78 (2013).

Borghetti A, Baldin G, Ciccullo A, Gagliardini R, Davino A, Mondi A, et al.   Virological control and metabolic improvement in HIV-infected, virologically suppressed patients switching to lamivudine/dolutegravir dual therapy. J Antimicrob Chem 2016; 71: 2359-2361. DOI: 10.1093/jac/dkw147

Brambilla, D. et al. The contribution of assay variation and biological variation to the total variability of plasma HIV-1 RNA measurements. Aids 13, 2269-2279 (1999).

Cambiano, V. et al. Assessment of the potential impact and cost-effectiveness of self-testing for HIV in low-income countries. Journal of Infectious Diseases, jiv040 (2015).

Cambiano, V. et al. Long-term trends in adherence to antiretroviral therapy from start of HAART. Aids 24, 1153-1162 (2010).

Cambiano, V. et al. Predicted levels of HIV drug resistance: potential impact of expanding diagnosis, retention, and eligibility criteria for antiretroviral therapy initiation. Aids 28, S15-S23 (2014).

Cambiano, V. et al. Use of a prescription-based measure of antiretroviral therapy adherence to predict viral rebound in HIV-infected individuals with viral suppression. HIV medicine 11, 216-224 (2010).

Cambiano, V., Bertagnolio, S., Jordan, M. R., Lundgren, J. D. & Phillips, A. Transmission of drug resistant HIV and its potential impact on mortality and treatment outcomes in resource-limited settings. Journal of Infectious Diseases 207, S57-S62 (2013).

Cambiano V. PhD thesis. http://discovery.ucl.ac.uk/1458628/1/PhD_CambianoValentina_25Sept_minor_corrections_clean_doubleside%20-%20without%20copyright%20figures.pdf

Carrieri, P. et al. The dynamic of adherence to highly active antiretroviral therapy: results from the French National APROCO cohort. JAIDS Journal of Acquired Immune Deficiency Syndromes 28, 232-239 (2001).

Castro, H. et al. Persistence of HIV-1 transmitted drug resistance mutations. Journal of Infectious Diseases 208, 1459-1463 (2013).

Charurat, M. et al. Patient retention and adherence to antiretrovirals in a large antiretroviral therapy program in Nigeria: a longitudinal analysis for risk factors. PLoS One 5, e10584 (2010).

Cheeseman, S. H. et al. Pharmacokinetics of nevirapine: initial single-rising-dose study in humans. Antimicrobial agents and chemotherapy 37, 178-182 (1993).

Chi, B. H. et al. Adherence to first-line antiretroviral therapy affects non-virologic outcomes among patients on treatment for more than 12 months in Lusaka, Zambia. International Journal of Epidemiology 38, 746-756 (2009).

CIA world factbook (2014) https://www.cia.gov/library/publications/the-world-factbook/geos/zi.html

Clotet B, Feinberg J, van Lunzen J, Khuong-Josses MA, Antinori A, Dumitru I, et al. Once-daily dolutegravir versus darunavir plus ritonavir in antiretroviral-naive adults with HIV-1 infection (FLAMINGO): 48 week results from the randomised open-label phase 3b study. Lancet. 2014; 383(9936):2222–31. doi: 10.1016/S0140-6736(14)60084-2 PMID: 24698485

Cohen, M. S. Sexually transmitted diseases enhance HIV transmission: no longer a hypothesis. The Lancet 351, S5-S7 (1998).

Corvasce, S. et al. Evidence of differential selection of HIV-1 variants carrying drug-resistant mutations in seroconverters. Antiviral therapy 11, 329 (2006).

de Boer M, van den Berk GEL, van Holten N, Oryszcyn JE, Dorama W, ait Mohab D, et al. Intolerance of dolutegravir-containing combination antiretroviral therapy regimens in real-life clinical practice. AIDS 2016, 30:2831–2834.

Deeks, S. G. et al. Persistence of drug-resistant HIV-1 after a structured treatment interruption and its impact on treatment response. Aids 17, 361-370 (2003).

DeGruttola, V. et al. The relation between baseline HIV drug resistance and response to antiretroviral therapy: re-analysis of retrospective and prospective studies using a standardized data analysis plan. Antiviral therapy 5, 41-48 (2000).

Devereux, H. L., Emery, V. C., Johnson, M. A. & Loveday, C. Replicative fitness in vivo of HIV-1 variants with multiple drug resistance-associated mutations. Journal of medical virology 65, 218-224 (2001).

Devereux, H. L., Youle, M., Johnson, M. A. & Loveday, C. Rapid decline in detectability of HIV-1 drug resistance mutations after stopping therapy. AIDS 13, 123-127 (1999).

Directors' Organisation, U. H. C. et al. Importance of age at infection with HIV-1 for survival and development of AIDS in UK haemophilia population. The Lancet 347, 1573-1579 (1996).

Doyle, T. & Geretti, A. M. Low-level viraemia on HAART: significance and management. Current opinion in infectious diseases 25, 17-25 (2012).

Drummond, M. F. Methods for the economic evaluation of health care programmes. (Oxford university press, 2005).

Dunkle, K. L. et al. New heterosexually transmitted HIV infections in married or cohabiting couples in urban Zambia and Rwanda: an analysis of survey and clinical data. The Lancet 371, 2183-2191 (2008).

Dunkle, K. L. et al. New heterosexually transmitted HIV infections in married or cohabiting couples in urban Zambia and Rwanda: an analysis of survey and clinical data. The Lancet 371, 2183-2191 (2008).

El-Khatib, Z. et al. Adherence and virologic suppression during the first 24 weeks on antiretroviral therapy among women in Johannesburg, South Africa-a prospective cohort study. BMC Public Health 11, 88 (2011).

Eron, J. J. et al. Treatment with lamivudine, zidovudine, or both in HIV-positive patients with 200 to 500 CD4+ cells per cubic millimeter. New England Journal of Medicine 333, 1662-1669 (1995).

Fonner, V. A., Denison, J., Kennedy, C. E., O'Reilly, K. & Sweat, M. Voluntary counseling and testing (VCT) for changing HIV-related risk behavior in developing countries. The Cochrane Library (2012).

Fox, M. P. et al. Rates and predictors of failure of first-line antiretroviral therapy and switch to second-line ART in South Africa. Journal of acquired immune deficiency syndromes (1999) 60, 428 (2012).

Fox, Z. et al. Viral re-suppression and detection of drug resistance following interruption of a suppressive NNRTI-based regimen. AIDS (London, England) 22, 2279 (2008).

Gallant, J. E. et al. Efficacy and safety of tenofovir DF vs stavudine in combination therapy in antiretroviral-naive patients: a 3-year randomized trial. JAMA 292, 191-201 (2004).

Gardner, E. M., Burman, W. J., Steiner, J. F., Anderson, P. L. & Bangsberg, D. R. Antiretroviral medication adherence and the development of class-specific antiretroviral resistance. AIDS (London, England) 23, 1035 (2009).

Genberg, B. L. et al. Patterns of antiretroviral therapy adherence and impact on HIV RNA among patients in North America. AIDS (London, England) 26, 1415 (2012).

Glass, T. R. et al. Longitudinal analysis of patterns and predictors of changes in self-reported adherence to antiretroviral therapy: Swiss HIV Cohort Study. JAIDS Journal of Acquired Immune Deficiency Syndromes 54, 197-203 (2010).

Government of Malawi Ministry of Health. Integrated HIV Program Reports. Quarterly.

Gregson, S. et al. HIV decline in Zimbabwe due to reductions in risky sex? Evidence from a comprehensive epidemiological review. International Journal of Epidemiology, dyq055 (2010).

Gregson, S., Zhuwau, T., Ndlovu, J. & Nyamukapa, C. A. Methods to reduce social desirability bias in sex surveys in low-development settings: experience in Zimbabwe. Sexually transmitted diseases 29, 568-575 (2002).

Grinsztejn, B. et al. Safety and efficacy of the HIV-1 integrase inhibitor raltegravir (MK-0518) in treatment-experienced patients with multidrug-resistant virus: a phase II randomised controlled trial. The Lancet 369, 1261-1269 (2007).

Gross, R., Bilker, W. B., Friedman, H. M. & Strom, B. L. Effect of adherence to newly initiated antiretroviral therapy on plasma viral load. Aids 15, 2109-2117 (2001).

Gross, R., Bilker, W. B., Friedman, H. M. & Strom, B. L. Effect of adherence to newly initiated antiretroviral therapy on plasma viral load. Aids 15, 2109-2117 (2001).

Gross, R., Bilker, W. B., Wang, H. & Chapman, J. How long is the window of opportunity between adherence failure and virologic failure on efavirenz-based HAART? HIV clinical trials 9, 202-206 (2008).

Gubavu C, Prazuck T, Niang M, Buret J, Mille C, Guinard J, Avettand-Fenoel V, Hocqueloux L et al. Dolutegravir-based monotherapy or dual therapy maintains a high proportion of viral suppression

even in highly experienced HIV-1-infected patients. J Antimicr Chem 2016; 71: 1046-1050. DOI: 10.1093/jac/dkv430

Halperin, D. T. et al. A surprising prevention success: why did the HIV epidemic decline in Zimbabwe. PLoS medicine 8, e1000414 (2011).

Hamers, R. L. et al. HIV-1 drug resistance in antiretroviral-naive individuals in sub-Saharan Africa after rollout of antiretroviral therapy: a multicentre observational study. The Lancet infectious diseases 11, 750-759 (2011).

Hance, A. J. et al. Changes in human immunodeficiency virus type 1 populations after treatment interruption in patients failing antiretroviral therapy. Journal of virology 75, 6410-6417 (2001).

Hance, A. J. et al. Changes in human immunodeficiency virus type 1 populations after treatment interruption in patients failing antiretroviral therapy. Journal of virology 75, 6410-6417 (2001).

Hance, A. J. et al. Changes in human immunodeficiency virus type 1 populations after treatment interruption in patients failing antiretroviral therapy. Journal of virology 75, 6410-6417 (2001).

Harrigan, P. R. et al. Predictors of HIV drug-resistance mutations in a large antiretroviral-naive cohort initiating triple antiretroviral therapy. Journal of Infectious Diseases 191, 339-347 (2005).

Hassan, A. S. et al. HIV-1 virologic failure and acquired drug resistance among first-line antiretroviral experienced adults at a rural HIV clinic in coastal Kenya: a cross-sectional study. AIDS Res Ther 11, 9 (2014).

Havlir, D., McLaughlin, M. M. & Richman, D. D. A pilot study to evaluate the development of resistance to nevirapine in asymptomatic human immunodeficiency virus-infected patients with CD4 cell counts of> 500/mm3: AIDS Clinical Trials Group Protocol 208. Journal of Infectious Diseases 172, 1379-1383 (1995).

Henrard, D. R. et al. Natural history of HIV-1 cell-free viremia. Jama 274, 554-558 (1995).

Hensen, B. et al. Universal voluntary HIV testing in antenatal care settings: a review of the contribution of provider-initiated testing & counselling. Tropical Medicine & International Health 17, 59-70 (2012).

Hill, A., McBride, A., Sawyer, A. W., Clumeck, N. & Gupta, R. K. Resistance at virological failure using boosted protease inhibitors versus nonnucleoside reverse transcriptase inhibitors as first-line antiretroviral therapy—implications for sustained efficacy of ART in resource-limited settings. Journal of Infectious Diseases 207, S78-S84 (2013).

Hoffmann, C. J. et al. Durable HIV RNA resuppression after virologic failure while remaining on a first-line regimen: a cohort study. Tropical Medicine & International Health 19, 236-239 (2014).

Hoffmann, C. J. et al. Viremia, resuppression, and time to resistance in human immunodeficiency virus (HIV) subtype C during first-line antiretroviral therapy. Clinical infectious diseases 49, 1928-1935 (2009).

Hoffmann C, Welz T, Sabranski M, Kolb M, Wolf E, Stellbrink1 H-J et al. Higher rates of neuropsychiatric adverse events leading to dolutegravir discontinuation in women and older patients. HIV Medicine 2017; 18, 56—63.

Hollingsworth, T. D., Anderson, R. M. & Fraser, C. HIV-1 transmission, by stage of infection. Journal of Infectious Diseases 198, 687-693 (2008).

Hubert, J.-B. et al. Natural history of serum HIV-1 RNA levels in 330 patients with a known date of infection. Aids 14, 123-131 (2000).

Huerga H. MSF. Mbongolwane and Eshowe HIV Impact in Population Survey http://www.epicentre.msf.org/en/mbongolwane-and-eshowe-hiv-impact-population-survey-0

Hyle, E. P. et al. The clinical and economic impact of point-of-care CD4 testing in Mozambique and other resource-limited settings: a cost-effectiveness analysis. (2014).

Jain V, Sucupira MC, Bacchetti P, Hartogensis W, Diaz RS, Kallas EG et al. Differential Persistence of Transmitted HIV-1 Drug Resistance Mutation Classes.   Journal of Infectious Diseases 203:1174–81 (2011)

Johannessen, A. et al. Virological efficacy and emergence of drug resistance in adults on antiretroviral treatment in rural Tanzania. BMC infectious diseases 9, 108 (2009).

Johnson, L. F., Dorrington, R. E., Bradshaw, D., Pillay-Van Wyk, V. & Rehle, T. M. Sexual behaviour patterns in South Africa and their association with the spread of HIV: insights from a mathematical model. Demographic Research 21, 289 (2009).

Johnston, V. et al. Outcomes following virological failure and predictors of switching to second-line antiretroviral therapy in a South African treatment programme. Journal of acquired immune deficiency syndromes (1999) 61 (2012).

Justman JE, Hoos D, Kalton G, Nyirenda R, Moyo C, Mugurungi O, Voetsch A, Patel H, Porter L. Real progress in the HIV epidemic: PHIA findings from Zimbabwe, Malawi and Zambia. Conference on Retroviruses and Opportunistic Infections. Feb 2017. Abstract Number: 114LB. http://www.croiwebcasts.org/console/player/33590?mediaType=audio&

Katlama C, Soulie C, Caby F, Denis A, Blanc C, Schneider L, et al. Dolutegravir as monotherapy in HIV-1-infected individuals with suppressed HIV viraemia. JOURNAL OF ANTIMICROBIAL CHEMOTHERAPY 2016; 71: 2646-2650 DOI: 10.1093/jac/dkw186.

Keebler, D. et al. How should HIV programmes monitor adults on ART? A combined analysis of three mathematical models. (2013).

Kleeberger, C. A. et al. Changes in adherence to highly active antiretroviral therapy medications in the Multicenter AIDS Cohort Study*. Aids 18, 683-688 (2004).

Kobin, A. B. & Sheth, N. U. Levels of adherence required for virologic suppression among newer antiretroviral medications. Annals of Pharmacotherapy 45, 372-379 (2011).

Kobin, A. B. & Sheth, N. U. Levels of adherence required for virologic suppression among newer antiretroviral medications. Annals of Pharmacotherapy 45, 372-379 (2011).

Koot, M. et al. Prognostic value of HIV-1 syncytium-inducing phenotype for rate of CD4+ cell depletion and progression to AIDS. Annals of internal medicine 118, 681-688 (1993).

Kranzer, K. & Ford, N. Unstructured treatment interruption of antiretroviral therapy in clinical practice: a systematic review. Tropical Medicine & International Health 16, 1297-1313 (2011).

Kranzer, K. et al. Treatment interruption in a primary care antiretroviral therapy programme in South Africa: cohort analysis of trends and risk factors. Journal of acquired immune deficiency syndromes 55, e17 (2010).

Kuritzkes, D. R. et al. Drug resistance and virologic response in NUCA 3001, a randomized trial of lamivudine (3TC) versus zidovudine (ZDV) versus ZDV plus 3TC in previously untreated patients. Aids 10, 975-981 (1996).

Larder, B. A. Viral resistance and the selection of antiretroviral combinations. JAIDS Journal of Acquired Immune Deficiency Syndromes 10, S34 (1995).

Lazo, M. et al. Patterns and predictors of changes in adherence to highly active antiretroviral therapy: longitudinal study of men and women. Clinical Infectious Diseases 45, 1377-1385 (2007).

Ledergerber, B. et al. Clinical progression and virological failure on highly active antiretroviral therapy in HIV-1 patients: a prospective cohort study. The Lancet 353, 863-868 (1999).

Levine, A. J. et al. Variations in patterns of highly active antiretroviral therapy (HAART) adherence. AIDS and Behavior 9, 355-362 (2005).

Llibre JM, Pulido F, Garcia F, Garcia Deltoro M, Blanco JL, Delgado R. Genetic barrier to resistance for dolutegravir. AIDS Rev. 2015; 17(1):56–64. PMID: 25472016 7.

Li, J. Z. et al. Incomplete adherence to antiretroviral therapy is associated with higher levels of residual HIV-1 viremia. AIDS (London, England) 28, 181 (2014).

Lyles, R. H. et al. Natural history of human immunodeficiency virus type 1 viremia after seroconversion and proximal to AIDS in a large cohort of homosexual men. Journal of Infectious Diseases 181, 872-880 (2000).

Maggiolo F, Gulminetti R, Pagnucco L, Digaetano M, Benatti S, Valenti D, et al. lamivudine/dolutegravir dual therapy in HIV-infected, virologically suppressed patients. BMC Inf Dis 2017; 17 DOI: 10.1186/s12879-017-2311-2

Malawi Demographic and Health Survey. 2010. https://dhsprogram.com/pubs/pdf/FR247/FR247.pdf

Maman D, Chilima B, Masiku C, Ayouba A, Masson S, Szumilin E, et al. Closer to 90:90:90. The cascade of care after 10 years of ART scale-up in rural Malawi: a population study. JIAS 2016 19:20673 http://dx.doi.org/10.7448/IAS.19.1.20673

Mannheimer, S., Friedland, G., Matts, J., Child, C. & Chesney, M. The consistency of adherence to antiretroviral therapy predicts biologic outcomes for human immunodeficiency virus—infected persons in clinical trials. Clinical infectious diseases 34, 1115-1121 (2002).

Marcellin A, Grude G, Charpentier C, Bellecave P, Rodallec A, Pallier C, et al. French survey, resistance to integrs inhib shows differences of resistance selection in virological failure in care. Abst 332. HIV Glasgow Octover 2016.

McMahon, J. H., Elliott, J. H., Bertagnolio, S., Kubiak, R. & Jordan, M. R. Viral suppression after 12 months of antiretroviral therapy in low-and middle-income countries: a systematic review. Bulletin of the World Health Organization 91, 377-385 (2013).

McMahon JH, Spelman T, Ford N, Greig J, Mesic A, Ssonko C, et al. Risk factors for unstructured treatment interruptions and association with survival in low to middle income countries. AIDS Res Ther (2016) 13:25. DOI 10.1186/s12981-016-0109-8

Mellors, J. W. et al. Plasma viral load and CD4+ lymphocytes as prognostic markers of HIV-1 infection. Annals of internal medicine 126, 946-954 (1997).

Menard A, Montagnac C, Solas C, Meddeb L, Dhiver D, Tomeia C, et al. Neuropsychiatric adverse effects on dolutegravir: an emerging concern in Europe AIDS 2017, Vol 31 No 8

Menzies NA, Berruti AA, Blandford JM (2012) The Determinants of HIV Treatment Costs in Resource Limited Settings. PLoS ONE 7(11): e48726. doi:10.1371/journal.pone.0048726

Meresse, M. et al. Patterns of adherence to antiretroviral therapy and HIV drug resistance over time in the Stratall ANRS 12110/ESTHER trial in Cameroon. HIV medicine 15, 478-487 (2014).

Mocroft, A. et al. Reasons for modification and discontinuation of antiretrovirals: results from a single treatment centre. Aids 15, 185-194 (2001).

Montaner, J. S. et al. A randomized, double-blind trial comparing combinations of nevirapine, didanosine, and zidovudine for HIV-infected patients: the INCAS Trial. Jama 279, 930-937 (1998).

Muyingo, S. K. et al. Patterns of individual and population-level adherence to antiretroviral therapy and risk factors for poor adherence in the first year of the DART trial in Uganda and Zimbabwe. JAIDS Journal of Acquired Immune Deficiency Syndromes 48, 468-475 (2008).

Nakagawa, F. et al. Projected life expectancy of people with HIV according to timing of diagnosis. Aids 26, 335-343 (2012).

Nakagawa, F. et al. Projected lifetime healthcare costs associated with HIV infection. (2015).

Nakagawa, F. PhD thesis.
http://discovery.ucl.ac.uk/1471810/13/PhD%20Thesis%20FN%20final_copyright_material_removed.pdf

National Institute for Communicable Diseases, Division of the National Health Laboratory Service. Prospective sentinel surveillance of human immunodeficiency virus related drug resistance.

Communicable Disease Communiqué. 2016 March; 15:10-11.
http://nicd.ac.za/assets/files/NICD%20Communicable%20Diseases%20Communique_Mar2016_final.pdf

Nicolè S, Lanzafame M, Lattuada E, Mazzi R, Rigo F, Cucchetto G, et al. Dolutegravir montherapy in HIV-infected naïve patients with <100,000 copies/ML HIV RNA load, an update of a little cohort in Verona. Infect Dis Trop Med 2016; 2 (2): e295.

Nicolosi, A. et al. The Efficiency of Male-to Female and Female-to-Male Sexual Transmission of the Human Immunodeficiency Virus: A Study of 730 Stable Couples. Epidemiology 5, 570-575 (1994).

O'Brien, T. R., Rosenberg, P. S., Yellin, F. & Goedert, J. J. Longitudinal HIV-1 RNA levels in a cohort of homosexual men. JAIDS Journal of Acquired Immune Deficiency Syndromes 18, 155-161 (1998).

Orrell, C. et al. Conservation of first-line antiretroviral treatment regimen where therapeutic options are limited. Antiviral therapy 12, 83 (2007).

Osterberg, L. & Blaschke, T. Adherence to medication. New England Journal of Medicine 353, 487-497 (2005).

Pantazis, N., Touloumi, G., Walker, A. & Babiker, A. Bivariate modelling of longitudinal measurements of two human immunodeficiency type 1 disease progression markers in the presence of informative drop-outs. Journal of the Royal Statistical Society: Series C (Applied Statistics) 54, 405-423 (2005).

Parienti, J.-J. et al. Effect of twice-daily nevirapine on adherence in HIV-1-infected patients: a randomized controlled study. Aids 21, 2217-2222 (2007).

Patel DA, Snedecor SJ, Tang WY, Sudharshan L, Lim JW, et al. 48-Week Efficacy and Safety of Dolutegravir Relative to Commonly Used Third Agents in Treatment-Naive HIV-1–Infected Patients: A Systematic Review and Network Meta-Analysis. PLoS ONE 2014 9(9): e105653. doi:10.1371/journal.pone.0105653.

Phillips, A. N. et al. Correspondence between the effect of zidovudine plus lamivudine on plasma HIV level/CD4 lymphocyte count and the incidence of clinical disease in infected individuals. AIDS 11, 169-175 (1997).

Phillips, A. N. et al. Effect on transmission of HIV-1 resistance of timing of implementation of viral load monitoring to determine switches from first to second-line antiretroviral regimens in resource-limited settings. Aids 25, 843-850 (2011).

Phillips, A. N. et al. HIV viral load response to antiretroviral therapy according to the baseline CD4 cell count and viral load. Jama 286, 2560-2567 (2001).

Phillips, A. N. et al. Increased HIV incidence in men who have sex with men despite high levels of ART-induced viral suppression: analysis of an extensively documented epidemic. PLoS One 8, e55312 (2013).

Phillips, A. N. et al. Outcomes from monitoring of patients on antiretroviral therapy in resource-limited settings with viral load, CD4 cell count, or clinical observation alone: a computer simulation model. The Lancet 371, 1443-1451 (2008).

Raboud, J. et al. Variation in plasma RNA levels, CD4 cell counts, and p24 antigen levels in clinically stable men with human immunodeficiency virus infection. Journal of Infectious Diseases 174, 191-194 (1996).

Resistance, U. C. G. o. H. D. & Group, U. C. S. Long term probability of detection of HIV-1 drug resistance after starting antiretroviral therapy in routine clinical practice. Aids 19, 487-494 (2005).

Resistance, U. C. G. o. H. D. & group, U. C. s. Long-term probability of detecting drug-resistant HIV in treatment-naive patients initiating combination antiretroviral therapy. Clinical Infectious Diseases 50, 1275-1285 (2010).

Rosen S, Fox MP. Retention in HIV care between testing and treatment in sub-Saharan Africa: a systematic review. *PLoS Med* 2011; **8(7)**:e1001056.

Rosenbloom, D. I., Hill, A. L., Rabi, S. A., Siliciano, R. F. & Nowak, M. A. Antiretroviral dynamics determines HIV evolution and predicts therapy outcome. Nature medicine 18, 1378-1385 (2012).

Rosenblum, M., Deeks, S. G., van der Laan, M. & Bangsberg, D. R. The risk of virologic failure decreases with duration of HIV suppression, at greater than 50% adherence to antiretroviral therapy. PLoS One 4, e7196 (2009).

Rowley CF, MacLeod IJ, Maruapula D, Lekoko B, Gaseitsiwe S, Mine M et al. Sharp increase in rates of HIV transmitted drug resistance at antenatal clinics in Botswana demonstrates the need for routine surveillance. J Antimicrob Chemother. 2016;71(5):1361–6.

Rutherford GW, Horvath H (2016) Dolutegravir Plus Two Nucleoside Reverse Transcriptase Inhibitors versus Efavirenz Plus Two Nucleoside Reverse Transcriptase Inhibitors As Initial Antiretroviral Therapy for People with HIV: A Systematic Review. PLoS ONE 11(10): e0162775. doi:10.1371/journal.pone.0162775

Sabapathy, K., Van den Bergh, R., Fidler, S., Hayes, R. & Ford, N. Uptake of home-based voluntary HIV testing in sub-Saharan Africa: a systematic review and meta-analysis. (2012).

Sabin, C. A. et al. Course of viral load throughout HIV-1 infection. JAIDS Journal of Acquired Immune Deficiency Syndromes 23, 172-177 (2000).

Salomon, J. A. et al. Common values in assessing health outcomes from disease and injury: disability weights measurement study for the Global Burden of Disease Study 2010. The Lancet 380, 2129-2143 (2013).

Sax PE, DeJesus E, Crofoot G, Ward D, Benson P, Dretler R. Bictegravir versus dolutegravir, each with emtricitabine and TDF alafenamide, for initial treatment of HIV-1 infection: a randomised, double-blind, phase 2 trial. Lancet HIV 2017; 4: e154–60

Siapka M, Remme M, Dayo Obure C, Maier C, Dehne KL, Vassall A. Is there scope for cost savings and efficiency gains in HIV services? A systematic review of the evidence from low- and middle-income countries. Bull World Health Organ 2014;92:499–511AD | doi: http://dx.doi.org/10.2471/BLT.13.127639

Sigaloff, K. C. et al. Accumulation of HIV drug resistance mutations in patients failing first-line antiretroviral treatment in South Africa. AIDS research and human retroviruses 28, 171-175 (2012).

Smith, D. M. et al. HIV drug resistance acquired through superinfection. Aids 19, 1251-1256 (2005).

Stadeli KM, Richman DD. Rates of emergence of HIV drug resistance in resource-limited settings: a systematic review. Antivir Ther. 18(1):115-23. doi: 10.3851/IMP2437 (2013).

Staszewski, S. et al. Determinants of sustainable CD4 lymphocyte count increases in response to antiretroviral therapy. Aids 13, 951-956 (1999).

Staszewski, S. et al. Virological response to protease inhibitor therapy in an HIV clinic cohort. Aids 13, 367-373 (1999).

Stellbrink H, Reynes J, Lazzarin A, Voronin E, Pulido F, Felizarta F, et al. Dolutegravir in antiretroviral-naive adults with HIV-1: 96-week results from a randomized dose-ranging study. AIDS 2013; 27:1771–1778

Stinnett, A. A. & Mullahy, J. Net health benefits a new framework for the analysis of uncertainty in cost-effectiveness analysis. Medical Decision Making 18, S68-S80 (1998).

Tagar E, Sundaram M, Condliffe K, Matatiyo B, Chimbwandira F, et al. (2014) Multi-Country Analysis of Treatment Costs for HIV/AIDS (MATCH): Facility-Level ART Unit Cost Analysis in Ethiopia, Malawi, Rwanda, South Africa and Zambia. PLoS ONE 9(11): e108304. doi:10.1371/journal.pone.0108304;

Taha H, Das A, Das S. Clinical effectiveness of dolutegravir in the treatment of HIV/AIDS. Infect Drug Resist. 2015;8:339–52. doi: 10.2147/IDR.S68396. pmid:26491363

Tarwater, P. M., Parish, M. & Gallant, J. E. Prolonged treatment interruption after immunologic response to highly active antiretroviral therapy. Clinical infectious diseases 37, 1541-1548 (2003)

Tassie, J.-M. et al. Trends in retention on antiretroviral therapy in national programs in low-income and middle-income countries. JAIDS Journal of Acquired Immune Deficiency Syndromes 54, 437-441 (2010).

The TenoRes Study Group. Global epidemiology of drug resistance after failure of WHO recommended fi rst-line regimens for adult HIV-1 infection: a multicentre retrospective cohort study. Lancet Infect Dis 2016

Touloumi, G. et al. Differences in HIV RNA levels before the initiation of antiretroviral therapy among 1864 individuals with known HIV-1 seroconversion dates. Aids 18, 1697-1705 (2004).

Tran, D. A. et al. Determinants of virological failure after 1 year's antiretroviral therapy in Vietnamese people with HIV: findings from a retrospective cohort of 13 outpatient clinics in six provinces. Sexually transmitted infections, sextrans-2013-051353 (2014).

Turner, D. et al. Diminished representation of HIV-1 variants containing select drug resistance-conferring mutations in primary HIV-1 infection. JAIDS Journal of Acquired Immune Deficiency Syndromes 37, 1627-1631 (2004).

Usitalo, A. et al. Relationship between viral load and self-report measures of medication adherence among youth with perinatal HIV infection. AIDS care 26, 107-115 (2014).

Vandepitte J, Lyerla R, Dallabetta G, Crabbe F, Alary M, Buve M. Estimates of the number of female sex workers in different regions of the world. Sex Transm Infect 2006;82(Suppl III):iii18–iii25. doi: 10.1136/sti.2006.020081

Van Leth, F. et al. Comparison of first-line antiretroviral therapy with regimens including nevirapine, efavirenz, or both drugs, plus stavudine and lamivudine: a randomised open-label trial, the 2NN Study. The Lancet 363, 1253-1263 (2004).

van Lunzen J, Maggiolo F, Arribas JR, Rakhmanova A, Yeni P, Young B, et al. Once daily dolutegravir (S/GSK1349572) in combination therapy in antiretroviral-naive adults with HIV: planned interim 48 week results from SPRING-1, a dose-ranging, randomised, phase 2b trial. Lancet Infect Dis 2012; 12: 111–18

von Wyl, V. et al. Adherence as a predictor of the development of class-specific resistance mutations: the Swiss HIV Cohort Study. PLoS One 8, e77691 (2013).

Wadonda-Kabondo N, Bennett D, van Oosterhout JJ et al. Prevalence of HIV Drug Resistance Before and 1 Year After Treatment Initiation in 4 Sites in the Malawi Antiretroviral Treatment Program. Clinical Infectious Diseases 54(S4):S362–8 (2012)

Wainberg MA, Han YS. Will drug resistance against dolutegravir in initial therapy ever occur? Front Pharmacol. 2015; 6:90. doi: 10.3389/fphar.2015.00090 PMID: 25972810

Wallis, C. L., Mellors, J. W., Venter, W. D., Sanne, I. & Stevens, W. Varied patterns of HIV-1 drug resistance on failing first-line antiretroviral therapy in South Africa. JAIDS Journal of Acquired Immune Deficiency Syndromes 53, 480-484 (2010).

Walmsley SL, Antela A, Clumeck N, Duiculescu D, Eberhard A, Gutierrez F, et al. Dolutegravir plus abacavir-lamivudine for the treatment of HIV-1 infection. N Engl J Med. 2013; 369(19):1807–18. doi: 10.1056/NEJMoa1215541 PMID: 24195548

Walmsley S, Baumgarten A, Berenguer J, Felizarta F, Florence E, Khuong-Josses MA, et al. Brief Report: Dolutegravir Plus Abacavir/lamivudine for the Treatment of HIV-1 Infection in Antiretroviral Therapy-Naive Patients: Week 96 and Week 144 Results From the SINGLE Randomized Clinical Trial. J Acquir Immune Defic Syndr. 2015;70(5):515–9. doi: 10.1097/QAI.0000000000000790. pmid:26262777

Walter, H. et al. No evidence for persistence of multidrug-resistant viral strains after a 7-month treatment interruption in an HIV-1-infected individual. Journal of acquired immune deficiency syndromes (1999) 31, 137-146 (2002).

Wandeler, G. et al. Outcomes of antiretroviral treatment programs in rural Southern Africa. Journal of acquired immune deficiency syndromes (1999) 59, e9 (2012).

Wanyenze, R. K. et al. Linkage to HIV care and survival following inpatient HIV counseling and testing. AIDS and Behavior 15, 751-760 (2011).

Wittkop, L. et al. Effect of Cytomegalovirus-Induced Immune Response, Self Antigen–Induced Immune Response, and Microbial Translocation on Chronic Immune Activation in Successfully Treated HIV Type 1–Infected Patients: The ANRS CO3 Aquitaine Cohort. Journal of Infectious Diseases 207, 622-627 (2013).

Wittkop, L. et al. Effect of transmitted drug resistance on virological and immunological response to initial combination antiretroviral therapy for HIV (EuroCoord-CHAIN joint project): a European multicohort study. The Lancet infectious diseases 11, 363-371 (2011).

Wijting I et al. Dolutegravir as maintenance monotherapy for HIV-1: a randomized clinical trial . (NCT02401828). CROI 2017, 13-16 February 2017, Seattle. Late breaker poster abstract 451LB.http://www.croiconference.org/sessions/dolutegravir-maintenance-monotherapy-hiv-1-randomized-clinical-trial (abstract and poster)

Woods, B., Revill, P., Sculpher, M. & Claxton, K. Country-level cost-effectiveness thresholds: initial estimates and the need for further research. (2015).

Yang W-L, Kouyos RD, Böni J, Yerly S, Klimkait T, Aubert V, et al. (2015) Persistence of Transmitted HIV-1 Drug Resistance Mutations Associated with Fitness Costs and Viral Genetic Backgrounds. PLoS Pathog 11(3): e1004722. doi:10.1371/journal.ppat.1004722
